# Silicon and Gibberellins: Synergistic Function in Harnessing ABA Signaling and Heat Stress Tolerance in Date Palm (*Phoenix dactylifera* L.)

**DOI:** 10.3390/plants9050620

**Published:** 2020-05-13

**Authors:** Adil Khan, Saqib Bilal, Abdul Latif Khan, Muhammad Imran, Raheem Shahzad, Ahmed Al-Harrasi, Ahmed Al-Rawahi, Masood Al-Azhri, Tapan Kumar Mohanta, In-Jung Lee

**Affiliations:** 1 Natural & Medical Sciences Research Center, University of Nizwa, Nizwa 616, Oman; adilsafi122333@gmail.com (A.K.); saqib043@yahoo.com (S.B.); ahmedalrawahi2@unizwa.edu.om (A.A.-R.); tapanmohanta@unizwa.edu.om (T.K.M.); 2 School of Applied Biosciences, Kyungpook National University, Daegu 41566, Korea; imran.khan2324@gmail.com; 3 Basic and Applied Scientific Research Center, Imam Abdulrahman Bin Faisal University, P.O. Box 1982, 31441 Dammam, Saudi Arabia; raheem.shehzad@ymail.com; 4 Department of Biology, College of Science, Imam Abdulrahman Bin Faisal University, P.O. Box 1982, 31441 Dammam, Saudi Arabia; 5 Agriculture Research Station, Jemaah, Nizwa 616, Oman; moharab@yahoo.com

**Keywords:** silicon, heat stress, gibberellins, date palm, oxidative stress

## Abstract

Date palm is one of the most economically vital fruit crops in North African and Middle East countries, including Oman. A controlled experiment was conducted to investigate the integrative effects of silicon (Si) and gibberellic acid (GA_3_) on date palm growth and heat stress. The exogenous application of Si and GA_3_ significantly promoted plant growth attributes under heat stress (44 ± 1 °C). The hormonal modulation (abscisic acid [ABA] and salicylic acid [SA]), antioxidant accumulation, and the expression of abiotic stress-related genes were evaluated. Interestingly, heat-induced oxidative stress was markedly reduced by the integrative effects of Si and GA_3_ when compared to their sole application, with significant reductions in superoxide anions and lipid peroxidation. The reduction of oxidative stress was attributed to the enhancement of polyphenol oxidase, catalase, peroxidase, and ascorbate peroxidase activities as well as the upregulation of their synthesis related genes expression viz. *GPX2*, *CAT*, *Cyt-Cu/Zn SOD*, and *glyceraldehyde3-phosphate dehydrogenase* gene (*GAPDH*). The results showed the activation of heat shock factor related genes (especially *HsfA3*) during exogenous Si and GA_3_ as compared to the control. Furthermore, the transcript accumulation of ABA signaling-related genes (*PYL4*, *PYL8*, and *PYR1*) were significantly reduced with the combined treatment of Si and GA_3_, leading to reduced production of ABA and, subsequently, SA antagonism via its increased accumulation. These findings suggest that the combined application of Si and GA_3_ facilitate plant growth and metabolic regulation, impart tolerance against stress, and offers novel stress alleviating strategies for a green revolution in sustainable food security.

## 1. Introduction

Numerous environmental stresses that adversely influence plant growth and productivity have raised serious concerns in the context of global climate change. In the wake of climate change, high temperature stress has emerged as a substantial limiting factor to plant productivity, ultimately leading to the weakening of food security worldwide. An average increase of 0.2 °C is predicted in global temperature per decade, which will result in approximately 224 °C higher temperature by the end of the 21st century [1,2]. This increase in temperature is deteriorating the growth and development of plants by inducing a series of morphological, physiological, and biochemical alterations and enabling conditions that favor oxidative damage. High temperature induce stress will become a further menace to plant growth and development by reducing soil moisture and subsequently leading to drought stress [3,4,5].

Heat stress-induced conditions often aggravate reactive oxygen species (ROS) production in plants, cause impairment to proteins, adversely influence plant synthesis, disrupt important enzymes activities, and induce lipid membrane damage as well as photosynthetic system dysfunction [3]. When confronted with heat stress, plants can alleviate stress-induced damages to some extent by initiating physical changes within their bodies and continuously creating signals to alter their metabolism, organize proteins, maintain cellular structures and cell turgor, and amend the antioxidant system in order to retain cellular redox balance [1]. Plants under heat stress activate heat-shock protein (HSP) genes in order to ensure the refolding of cellular proteins and conformational protein functions, thereby rescuing plants under heat stress [6]. Furthermore, the accumulation and signaling of abscisic acid (ABA) by plants is required for acquired thermotolerance via integration with other hormones and ROS regulatory systems [7].

Molecules that protect plants from the adverse effects of climate adversaries are attracting interest among plant researchers. Exogenous application of protectants, such as phytohormones, like gibberellic acids (GA), and trace elements, like silicon (Si), has been reported to be effective for ameliorating abiotic stress, including heat-induced damages in plants [1,8]. The exogenous application of GA_3_ was demonstrated to reverse the lethal effects of salt, heat, and oxidative stresses in Arabidopsis [9]. Likewise, the exogenous application of Si is considered to be crucial for the growth and development of plants under hostile environmental conditions. A previous study [10] also demonstrated the beneficial effects of exogenous application of Si on the physiological development of *Salvia splendens* under high temperature by improving the antioxidant system. Despite the abiotic stress ameliorative effects of Si and GA in plants, their interactive effects on plants with regard the mitigation of abiotic stresses, including heat stress through physiological and biochemical modulation, are relatively unknown. 

Date palm (*Phoenix dactylifera* L.) is a primary and vital fruit crop that significantly contributes to the economy of governments in arid and semi-arid regions of the world, including Oman. Oman is considered to be the eighth largest producer of date palm in the world, with an average annual production of 260,000 mt per annum [11]. Date palm comprises approximately 82% of all fruit crops cultivated in Oman, occupying 50% of the cultivated area [12]. In the context of climatic change, the cultivation of date palm has been drastically affected by environmental-induced abiotic stresses, including heat stress [12,13]. Date palm can withstand harsh climatic conditions, including various abiotic stresses, such as salinity, drought, and heat. However, excessive and prolonged levels of environmental stresses, including heat and drought, can lead to a significant reduction in the productivity and quality of fruits and the impairment of physiological and metabolic processes in plants seedlings [13,14].

Regardless of the environmental-induced stress alleviation effects of GA_3_ and Si, their exogenous application to date palm for boosting physiological and molecular responses to mitigate the adverse impacts of abiotic stresses, including heat stress, have rarely been explored. Therefore, the current study was aimed at elucidating the sole and integrative effects of exogenous application of GA_3_ and Si to date palm to alleviate heat stress. Several physiological and biochemical parameters, including endogenous phytohormonal changes and antioxidant defense modulation in date palm in response to heat stress, were studied to assess the effects of exogenous application of GA_3_ and Si. Moreover, the responses of HSP genes and ABA-related genes regulation due to physiological and biochemical modulation by GA_3_ and SA treatment under heat stress were also elucidated.

## 2. Methodology

### 2.1. Plant Growth and Treatment Conditions

The Agriculture Research Station, Bahla (Oman) provided date palm (*Phoenix dactylifera* L. cv. Khalas) seedlings (three months old). Before treatment, the seedlings were placed in a greenhouse for three months to equilibrate the seedling growth to a normal condition. At this stage, 50 mL distilled water was applied to each pot (10 × 9 cm) containing one seedling. Sphagnum peat moss was used (moisture content 38.5%, pH 4.5–5.5, electrical conductivity (EC) 2.0 dS m^−1^, bulk density 0.7–1.0 mg m^3^, organic matter 91.1% (*w*/*w*), nitrogen 800–2500 mg/kg, phosphorus 150–850 mg/kg, sodium (Na) 340 mg/kg, and sodium chloride (NaCl) 850 mg/kg). Thereafter, seedlings with uniform length and number of leaves were selected, and then arranged in the greenhouse in a completely randomized experiment with 20 seedlings. The seedlings were treated with Si (Na_2_SiO_3_; 1.0 mM) and GA_3_ (100 µM). These GA_3_ and Si concentrations were previously found to improve the plant growth and development of various crop plants during stress condition [15,16,17,18,19]; therefore, we adopted the same for this study. All of the solutions were prepared in distilled water. The pH value of all nutrient solutions was adjusted to 6.5. Si and GA_3_ (50 mL) were simultaneously applied to the plant’s root zone at concentrations determined during a previous experiment on rice and tomato seedlings. The seedlings were subjected to one of four treatments: group 1 (control), group 2 (GA_3_; 100 µM); group 3 (Si; 1.0 mM); and Group 4 (Si + GA_3_; 1.0 mM and 100 µm). After the treatment period (30 days), half of the plants from each group were subjected to high temperature (44 °C). The growth chamber conditions were adjusted to 8 h of light at 28–30 °C (08:00~16:00; relative humidity 60%), 6 h of light at 44 °C (16:00~22:00; gradient increase of 5 °C per hour; relative humidity 60%), and 10 h of darkness at 28 °C (22:00~08:00; relative humidity 70%). After treatment for six weeks, the growth attributes were recorded (shoot length, diameter, and number of leaves). The plants were harvested in liquid nitrogen and they were stored at −80 °C. The experiment was repeated three times, each with five replications.

### 2.2. Chlorophyll a and Chlorophyll b Quantification

Photosynthetic pigments, including Chl *a*, Chl *b*, and carotenoid, were extracted by grinding the leaves of the date palm seedlings (200 mg) in 80% acetone. The methodology described by Sumanta, et al. [20] was employed to estimate the Chl *a* and Chl *b* content. The absorbance values for Chl *a*, Chl *b*, and carotenoid were recorded at 663, 645, and 470 nm, respectively.

### 2.3. Leaf Relative Water Content (LRWC)

The protocol that was established by Cao, et al. [21] was used for the estimation of RWC. For each treatment, the second leaves of the plants were excised, and the fresh mass (FM) was immediately quantified. Thereafter, leaf discs were floated on 30 mL deionized water for 5 h in a petri dish and the saturated mass (SM) was determined. Subsequently, the leaves were dried at 80 °C to a constant weight and their dry mass (DM) was measured. The RWC was calculated with the following formula: RWC [%] = [(FM − DM)/(SM − DM)] × 100.

### 2.4. Quantification of Malondialdehyde (MDA)

The level of lipid peroxidation or formation of MDA was estimated using the methodology that was reported by Okaichi, et al. [22]. The tissue homogenates were extracted with 10 mM phosphate buffer (pH 7.0). For the quantification of MDA, 0.2 mL of tissue homogenate was combined with 0.2 mL of 8.1% sodium dodecyl sulfate (SDS), 1.5 mL of 20% acetic acid (pH 3.5), and 1.5 mL of 0.81% thiobarbituric aqueous acid (TBA) solution in a reaction tube. Thereafter, the mixture was heated in boiling water for 60 min. After cooling to room temperature, 5 mL butanol:pyridine (15:1 *v*/*v*) solution was added. The upper organic layer was separated and the optical density of the resulting pink solution was recorded at 532 nm while using a spectrophotometer. Tetramethoxypropane was used as an external standard. 

### 2.5. Determination of Superoxide (O_2_^•−^)

The level of O_2_^•−^ was estimated using the method that was described by Gajewska and Skłodowska [23]. The homogenate for the reaction was prepared by immersing 1 g of fresh plant sample in phosphate buffer (pH 7.0) containing sodium phosphate (10 mM), nitrobluetetrazolium (NBT) (0.05%; *w*/*v*), and sodium azide (NaN_3_) (10 mM). The mixture was placed at room temperature for 1 h. Afterwards, 5 mL of the mixture was taken in a new tube and heated for 15 min at 85 °C. Thereafter, the mixture was cooled and vacuum filtered. The absorbance of the sample was read at 580 nm with a spectrophotometer. The experiment was replicated three times.

### 2.6. Protein Quantification and Antioxidant Enzyme Assay

For protein quantification, the leaf samples from the date palm (100 mg) were ground using a chilled mortar pestle in 100 mM potassium phosphate buffer (pH 6.8) with 0.2 mM ethylenediaminetetraacetic acid (EDTA). The resulting mixture was centrifuged for 30 min at 12,000× *g* and the supernatant were used to determine the total protein. The total protein contents were estimated following the protocol that was reported by Bradford [24], with slight modifications. The assay was performed at 595 nm with a spectrophotometer.

The antioxidant enzymes catalase (CAT), ascorbate peroxidase (APX), and polyphenol peroxidase (PPO) were quantified with the methodology that was described by Manoranjan, et al. [25], with slight changes. In brief, date palm leaves (100 mg) were ground by using liquid nitrogen and Phosphate buffer (100 mM) was added to the sample to make a homogenous mixture of pH 7.0. The resulting homogenate were centrifuged for 30 min at 10,000 rpm and 4 °C.

For POD (Peroxidase) analysis, the reaction mixture consisted of 0.1 M potassium phosphate buffer (pH 6.8), 50 μL H_2_O_2_ (50 µM), 50 μL pyrogallol (50 µM), and 100 μL crude extract sample. This mixture was incubated at 25 °C for 5 min, then 5% H_2_SO_4_ (*v*/*v*) was added to stop the enzymatic reaction. The amount of purpurogallin produced was measured while using an optical density of 420 nm. The reaction mixture consisted of similar components as the POD assay, but without H_2_O_2_, and the final assay was calculated at 420 nm, in order to assess the polyphenol oxidase (PPO) activity. A single unit of PPO and POD was directly calculated using an increase of 0.1 units of absorbance. The CAT activity was assayed, as described by Aebi [26]. Briefly, the crude enzyme extract was added to 0.2 M H_2_O_2_ in 10 mM phosphate buffer (pH 7.0), after which the CAT activity was determined as a decrease in absorbance at 240 nm and expressed as units (one unit of CAT was defined as the ng of H_2_O_2_ released/mg protein/min).

For the quantification of APX (Ascorbate peroxidase), 1 mL phosphate buffer (50 mM; pH 7.0) containing 1 mM ascorbic acid and 1 mM EDTA was used for extraction, then homogenized at 50 Hz for 30 s, and the homogenate was centrifuged at 4,830× *g* at 4 °C for 15 min. Subsequently, the supernatant was mixed with a phosphate buffer solution (pH 7.0) containing 15 mM AsA and 0.3 mM H_2_O_2_. The reaction mixture was analyzed at 290 nm. One unit of APX was defined as a variable quantity of absorbance at 290 nm per min. All of the enzymatic assays were repeated three times and each time comprised of three replications.

### 2.7. RNA Extraction and cDNA Synthesis

RNA was extracted from date palm leaves while using an extraction buffer (0.25 M, NaCl; 0.05 M, Tris-HCl (pH = 7.5); 20 mM, EDTA; 1% (*w*/*v*) SDS; 4% polyvinylpyrrolidone (*w*/*v*)), as described by Liu, et al. [27]. Prior to the addition of the sample, 750 μL of the extraction buffer and chloroform: isoamyl alcohol (CI; 24:1 v/v) were added to a 2-mL RNase-free microcentrifuge tube followed by the addition of 40 μL β-mercaptoethanol. Thereafter, a fine powder (100 mg) of the sample was carefully transferred to a 2 mL tube containing the extraction buffer and CI. The mixture was vortexed and incubated at 20 °C for 15 min, followed by centrifugation at 4 °C for 10 min at 12,000× *g*. In the next step, 600 μL of the supernatant was transferred to a new 2 mL tube and the same volume of CI was added to the tube. The solutions were mixed gently and centrifuged at 4 °C for 10 min at 12,000× *g.* The upper layer was transferred to a new 1.5 mL micro centrifuge tube and 1/10 volume of 3 M sodium acetate (pH = 5.2) was added. For precipitation, two volumes of absolute ethanol were added, and after gently mixing, the tubes were incubated for 45 min at 4 °C. After incubation, the samples were centrifuged at 4 °C for 10 min at 12,000× *g* and. The pellet was dissolved in 200 μL water (diethyl pyrocarbonate-treated) and 10 M LiCl was added (500 μL) to the solution. The solutions were mixed gently and then placed on ice for 60 min. In the final step, the samples were centrifuged at 4 °C for 10 min at 12,000× *g*, and the pellet was washed with 70% ethanol. After removing the ethanol, the pellet was air dried and then dissolved in 50 μL of diethyl pyrocarbonate-treated water. The quality of RNA was checked with agarose gel electrophoresis and quantified while using the Qubit (3.0) RNA broad range kit. RNA was added to the Master Mix according to the concentration; for each 100 ng/µL RNA, 10 µL was taken for cDNA synthesis. Polymerase chain reaction was performed in a thermo-cycler at specific conditions (25 °C for 10 min, 37 °C for 2 h, and 85 °C for 5 min). The synthesized cDNA was refrigerated at −80 °C until further use.

### 2.8. Gene Expression Analysis

The synthesized cDNA was used for the amplification of genes (Table 1). Actin gene was used as a reference for all of the primers. Power up “SYBR” green Master Mix was used for the thermo-cycler (Quant studio 5 by Applied Bio Systems Life Technologies) PCR reaction. Primers (10 pM; forward and reverse) were used for all of the five genes. For each sample, the reaction was performed in triplicate to minimize errors and contamination. The reaction was performed at a specific condition such as 94 °C for 10 min in stage 1, 35 cycles of PCR reaction at 94 °C for 45 s, 60 °C for 45 s, 72 °C for 1 min, and finally, 72 °C for 10 min. A threshold level of 0.1 was set for gene amplifications. The experiment was repeated three times and each time comprised of three replications.

### 2.9. Abscisic Acid Extraction and Quantification

For the extraction and quantification of endogenous ABA levels in date palm leaves, the protocol that was reported by Qi, et al. [28] was used with slight modification, as described by Bilal, et al. [29]. Briefly, the extracted samples from the ground and freeze-dried plants were supplemented with [(±)−3,5,5,7,7,7-d6]-ABA as an internal standard and then further analyzed with GCMS (6890N network GC system) and a 5973 Network Mass Selective Detector (Agilent Technologies, Palo Alto, CA, USA). The spectra were recorded at selected ionization values of m/z 162 and 190 for Me-ABA and at m/z 166 and 194 for Me-[2H6]-ABA to expand the affectability of the method. The ABA was calculated from the value of the endo peak in comparison with their respective standards.

### 2.10. Salicylic Acid Extraction and Quantification

Salicylic acid (SA) was extracted and quantified from freeze dried samples of the date palm leaves according to the protocol that was described by Seskar, et al. [30] and Bilal, et al. [31]. The extracted samples were subjected to High Performance Liquid Chromatography (HPLC), which was performed while using a Shimadzu device outfitted with a fluorescence indicator (Shimadzu RF-10AxL) with excitation at 305 nm and emission at 365 nm and with a C18 reverse-phase HPLC column (HP Hypersil ODS, particle size 5 μm, pore size 120 Å, Waters). The flow rate was maintained at 1.0 mL/min. 

### 2.11. Statistical Analysis

All of the experiments were repeated three times and the data were collected from each repetition were pooled together. All of the data present the mean values with standard error (SE). The means were analyzed for finding the significant differences among treatments by using one-way analysis of variance (ANOVA), followed by Duncan’s multiple range test (DMRT) in SAS software (V9.1, Cary, NC, USA; Figure S1).

## 3. Results

### 3.1. Interactive Effects of GA and Si Promote Plant Growth Attributes under Heat Stress

**Figure 1:**
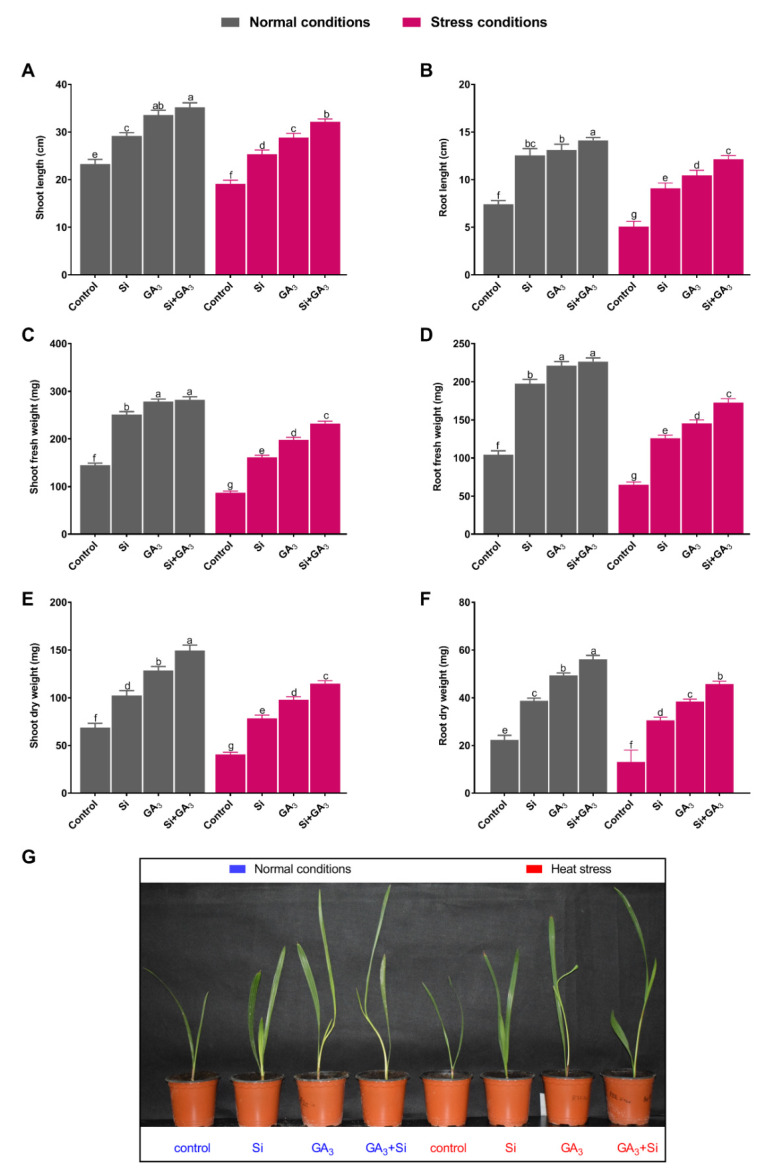
Application of sole silicon (Si) and gibberellic acid (GA_3_) and their integrative effects of effect on date palm growth under heat stress conditions. (**A**) Shoot length. **(B**) Root length. (**C**) Shoot fresh weight. (**D**) Root fresh weight. (**E**) Shoot dry weight. (**F**) Root dry weight. (**G**) Date palm seedling picture. Different letters indicate the values are significantly different (*p* < 0.05). Means were analyzed for finding the significant differences among treatments by using Duncan’s multiple range test (DMRT). Values represent means (of 10 replicates) ± standard error.

**Figure 2:**
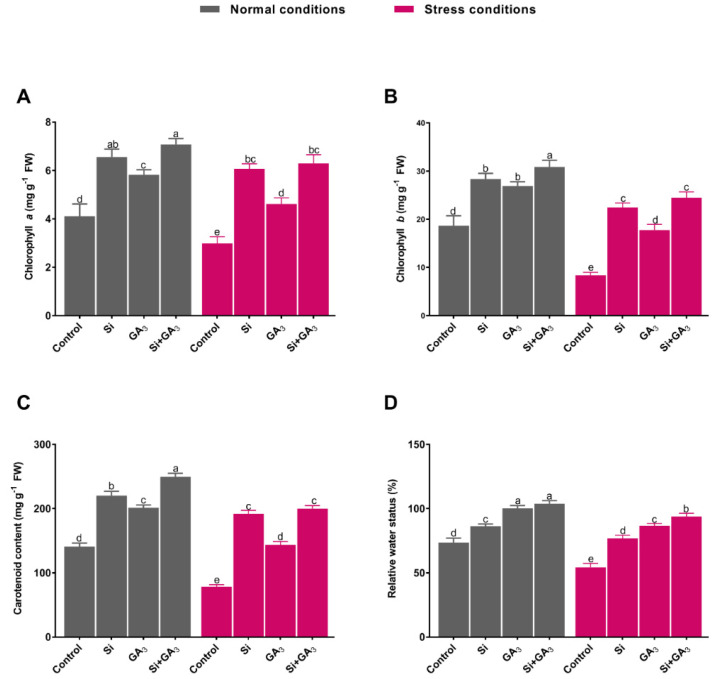
Influence of silicon (Si) and gibberellic acid (GA_3_) and their interaction on chlorophyll pigments and leaf relative water status in date palm under heat stress. (**A**) Chlorophyll *a*. (**B**) Chlorophyll *b*. (**C**) Carotenoid. (**D**) Relative water status. Different letters indicate the values are significantly different (*p* < 0.05). Means were analyzed for finding the significant differences among treatments by using one-way analysis of variance (ANOVA), followed by Duncan’s multiple range test (DMRT). Values represent means (of 6 replicates) ± standard error.

The combined exogenous application of GA_3_ and Si resulted in significant plant growth-promoting effects under the control conditions and rescued plant growth under heat stress compared to non-treated plants. The combined application of GA_3_ and Si resulted in the maximum shoot length (35.1 ± 0.94 cm) and root length (14.1 ± 0.29 cm) in the absence of stress conditions. Further, the interactive effects of GA_3_ and Si significantly mitigated the adverse impact of heat stress and resulted in the maximum shoot length (31.87 ± 0.59 cm) and root length (11.56 ± 0.38 cm), followed by the sole application of GA_3_ and Si (Figure 1A,B). Likewise, the fresh weight of shoot and root was detected to be maximum in both Sole GA_3_ and combined Si and GA_3_ treated plants under control conditions. However, heat stress markedly retarded the fresh weight of the shoot and root of all plants as compared to their respective treatments under control condition. Nevertheless, the combined application of GA and Si under heat stress enhanced the shoot fresh weight by 1.17, 1.44, and 2.66 times and the root fresh weight by 1.18, 1.37, and 2.68 times as compared to the sole GA and Si-treated plants and the non-treated plants, respectively (Figure 1C,D). The same trend was detected for the dry biomass of the shoot and the root of the combined GA3 and Si-treated plants under heat stress. The combined application of GA_3_ and Si induced the maximum enhancements in chlorophyll *a* and *b* when compared to sole Si or GA_3_-treated plants and non-treated plants under control conditions (Figure 1E–G). The exposure to heat stress substantially decreased the chlorophyll *a* and *b* content of non-treated plants, followed by that of sole GA_3_ and Si-treated plants. Under heat stress, the combined application of GA_3_ and Si resulted in a significantly higher level of the chlorophyll *a* and *b* content with the increases of 2.11 and 2.92 times when compared to the content in non-treated plants (Figure 2A,B). Under the control condition, the maximum carotenoids content was recorded with the combined application of GA_3_ and Si followed by sole Si or GA_3_ treatment and non-treatment. However, under stress conditions, combined GA_3_ and Si-treated plants and sole Si-treated plants equally demonstrated higher levels of carotenoids content, followed by sole GA_3_-treated plants and non-treated plants (Figure 2C). The heat stress-mitigating response of combined application of GA_3_ and Si was further assessed by measuring the relative water potential of the plants. The current findings indicated that combined Si and GA_3_ treatment as well as sole GA_3_ treatment resulted in the maximum RWC under the control condition. Heat stress drastically reduced the RWC of non-treated plants, with a reduction of 1.73, 1.51, and 1.42 times when compared to the combined GA_3_ and Si-treated plants and the sole GA_3_ and Si-treated plants, respectively (Figure 1D).

### 3.2. Interactive Effects of GA_3_ and Si Stimulate Plant Antioxidant System

**Figure 3:**
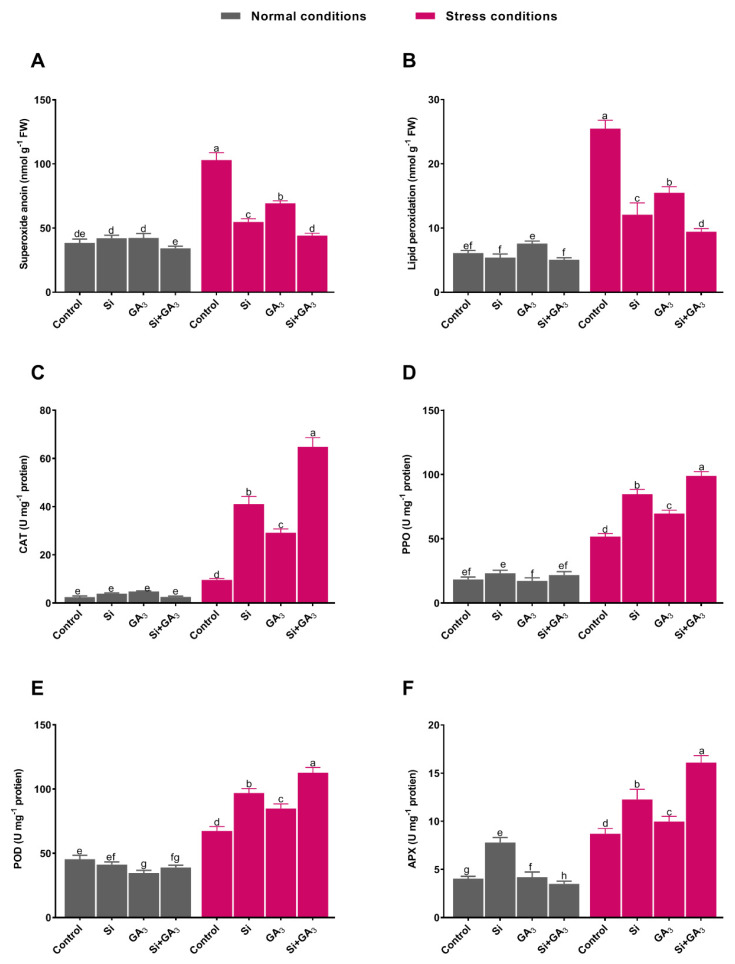
Exogenous application of silicon (Si) and gibberellic acid (GA_3_) and their interaction modulates lipid peroxidation (malondialdehyde, MDA) and antioxidants (peroxidase, POD; ascorbate peroxidase, APX; catalase, CAT and polyphenol oxidase; PPO) of date palm under heat stress. (**A**) Super oxide anion. **(B**) Lipid peroxidation. (**C**) Catalase. **(D**) Polyphenol oxidase. (**E**) Peroxidase. (**F**) Ascorbate peroxidase. Bars with different letters have significantly different (*p* > 0.05) means by using one-way analysis of variance (ANOVA), followed by Duncan’s multiple range test (DMRT). Values represent mean (of four replicates) ± standard error.

The extent of O_2_^•−^ was investigated in the date palm plants to assess the generation of heat-induced reactive oxygen species. Heat-induced stress is known to trigger O_2_^•−^ accumulation in plants. The current findings indicated that exposure to heat stress resulted in the significant production of O_2_^•−^ in plants by showing maximum superoxide anion activity (Figure 3A). However, the negative influence of heat stress was markedly mitigated after Si and GA_3_ treatment. The accumulation of O2^•−^ was significantly retarded by the combined application of Si and GA_3_, with 2.3, 1.5, and 1.2 times less superoxide anion activity when compared to non-treated plants and sole GA_3_ and Si-treated plants, respectively. Similarly, the extent of lipid membrane peroxidation due to heat stress damages was investigated by measuring the MDA content. Under control conditions, no treatment was significantly different from the non-treated control; however, GA_3_ treatment differed from sole Si and combined Si and GA_3_ treatments. Moreover, the exposure to heat stress substantially increased the level of MDA in non-treated plants, whereas the combined application of GA_3_ and Si significantly decreased the MDA level, followed by sole Si and GA_3_ treatment (Figure 3B). The antioxidant activities (CAT, POD, PPO, and APX) were measured to further characterize the combined effects of GA_3_ and Si on overcoming heat-induced oxidative stress (Figure 3C–F). The results showed that the combined application of GA_3_ and Si significantly triggered (*p* > 0.05) CAT activity under heat stress when compared to non-treatment. Similarly, PPO activities for all of the treatments were enhanced under heat stress; however, the combined application of GA_3_ and Si resulted in approximately 1.91, 1.43, and 1.16 times higher activity than non-treatment and sole GA_3_ and Si treatment, respectively. Furthermore, POD activity was significantly depressed in GA_3_ treatment, while non-treated plants and sole Si-treated plant exhibited insignificant levels of POD activity under control conditions. Whereas, the level of POD activity of combined Si and GA_3_ treatment was approximately comparable with sole Si treatment under control conditions. Whilst the maximum POD activity was recorded with combined application of GA_3_ and Si, followed by sole Si and GA_3_ treatment under heat stress. Under control conditions, the maximum APX activity was recorded in sole Si-treated plants, while non-treated plants, sole GA_3_-treated plants, and combined Si and GA-treated plants exhibited comparable APX activity. However, the combined application of Si and GA_3_ resulted in the maximum APX activity under heat stress, with approximately 1.91, 1.61, and 1.31 times higher activity than the non-treatment and sole GA_3_ and Si treatment (Figure 3C–F).

### 3.3. Interactive Effects of Si and GA_3_ Modulate Endogenous Hormonal Regulation

**Figure 4:**
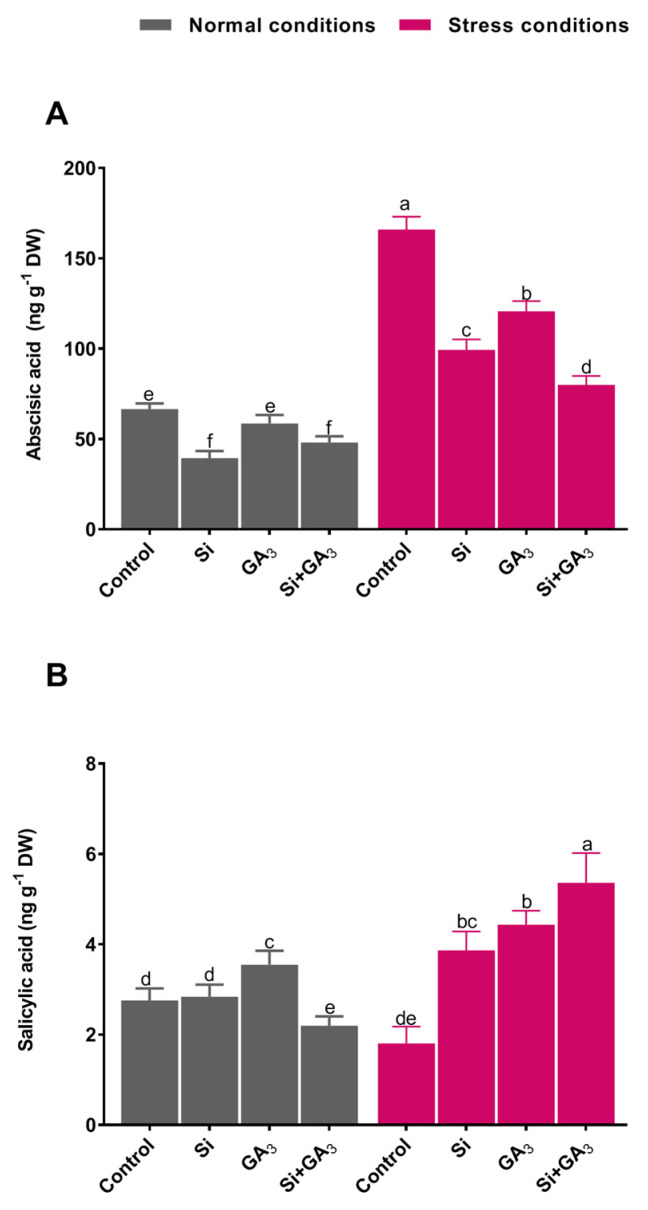
Regulation of endogenous hormones (abscisic acid and salicylic acid) of date palm under heat stress by the exogenous application of silicon (Si) and gibberellic acid (GA_3_) and their interaction. (**A**) Abscisic acid. (**B**) Salicylic acid. Different letters indicate the values are significantly different (*p* < 0.05). Means were analyzed for finding the significant differences among treatments by using one-way analysis of variance (ANOVA), followed by Duncan’s multiple range test (DMRT). Values represent mean (of four replicates) ± standard error.

Endogenous ABA analysis revealed that the sole application of Si and combined application of Si and GA_3_ considerably decreased free ABA content in date palm as compared to non-treatment and sole GA_3_ treatment under control conditions (Figure 4A). On the contrary, the level of ABA accumulation was significantly enhanced under heat stress with all of the treatments. However, a significantly low level of ABA was observed in the combined GA_3_ and Si-treated plants when compared to the non-treated plants and the sole GA_3_ or Si-treated plants. The combined GA_3_ and Si-treated plants had approximately 2.06, 1.24, and 1.50 times lower ABA content under heat stress when compared to the non-treated and Si and GA_3_-treated plants, respectively. Similarly, analysis showed that the level of SA accumulation under the control condition was almost comparable in all treatments, except for the sole GA_3_-treated plants, which had the maximum (3.5 ± 0.31 ng/g) accumulation. On the other hand, heat stress significantly downregulated SA accumulation in non-treated plants, whereas the interactive effects of GA_3_ and Si significantly upregulated SA content (5.3 ± 0.65 ng/g) in date palm, followed by sole GA_3_-treatment and Si treatment (Figure 4B).

### 3.4. Modulation of Different Stress-Responsive Genes by Interactive Effects of Si and GA_3_ Application

**Figure 5:**
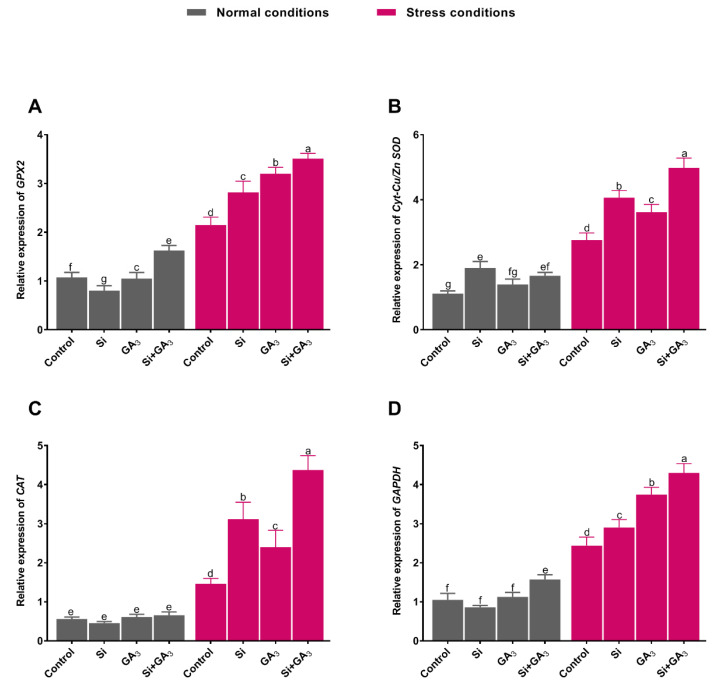
Effects of heat stress on the expression of antioxidant related genes in date palm. (**A**) Glutathione peroxidase. (**B**) Superoxide dismutase [Cu-Zn]-like. (**C**) Catalase. (**D**) NADP-Dependentglyceraldehyde-3-phosphatedehydrogenase. Total RNA was extracted from date palm seedlings grown under normal and heat stress conditions with/without exogenously applied silicon (Si) and gibberellic acid (GA_3_) and their combination. Transcript levels were measured by real-time qPCR. *Actin* was used as an internal control. Bars represent mean (of four replicates) ± standard error. Different letters indicate the values are significantly different (*p* < 0.05). Means were analyzed for finding the significant differences among treatments by using one-way analysis of variance (ANOVA) followed by Duncan’s multiple range test (DMRT).

The interactive effects of Si and GA_3_ application on the transcript levels of different abiotic stress-related genes were assessed. The expression level of the antioxidant-related gene glutathione peroxidase (*GPX2*) was significantly higher in sole GA_3_-treated plants under control conditions than in the combined GA_3_ and Si-treated plants, sole Si-treated plants, and non-treated plants (Figure 5A). The combined GA_3_ and Si-treated plants exhibited a drastic enhancement in the *GPX2* expression level under heat stress, followed by the sole GA_3_ and Si-treated plants, and non-treated plants. Similarly, *Cyt-Cu/Zn SOD* transcript accumulation was significantly enhanced in all treatments under heat stress when compared to the control condition (Figure 5B). However, the GA_3_ and Si-treated plants demonstrated significant (*p* < 0.005) enhancement in the expression level of *Cyt-Cu/Zn SOD* as compared to sole Si and GA_3_-treated and non-treated plants. Likewise, the CAT expression level under control conditions was comparable among all treatments; however, combined GA_3_ and Si-treated plants exhibited significantly enhanced levels under heat stress, followed by sole Si and GA3-treated plants and non-treated plants, respectively (Figure 5C). Likewise, the transcript accumulation level of *NADP-dependent glyceraldehyde3-phosphate dehydrogenase* gene (*GAPDH*) under the control condition was non-significant in all treatments except in the combined GA_3_ and Si-treated plants. However, *GAPDH* expression under heat stress was significantly up-regulated in all treatments when compared to the control condition (Figure 5D). The combined GA_3_ and Si-treated plants exhibited significant up-regulation, with 1.79, 1.48, and 1.16 times higher expression than the non-treated plants and the sole Si and GA_3_-treated plants, respectively.

**Figure 6:**
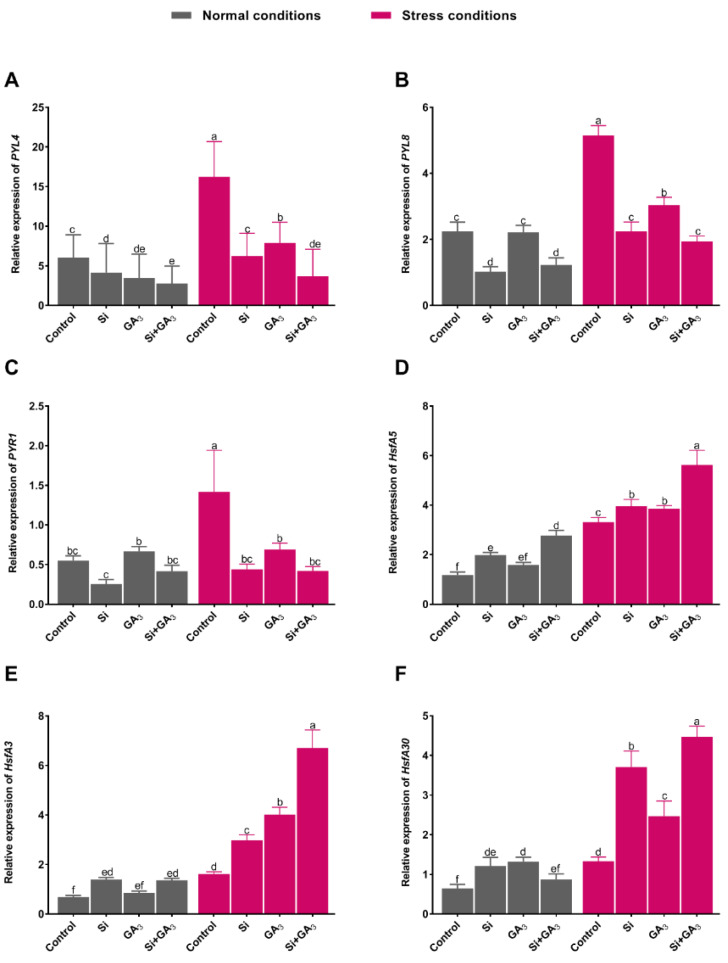
Effects of heat stress on the expression of antioxidant related genes in date palm. (**A**) Abscisic acid receptor PYL4-like. (**B**) Abscisic acid receptor PYL3-like. (**C**) Abscisic acid receptor PYR1. (**D**) Heat stress transcription factor A−5−like. **(E**) Heat stress transcription factor A−3. (**F**) Heat shock factor protein HSF30-like. Total RNA was extracted from date palm seedlings grown under normal and heat stress conditions with/without exogenously applied silicon (Si) and gibberellic acid (GA_3_) and their combination. Transcript levels were measured by real-time qPCR. Actin was used as an internal control. Bars represent mean (of four replicates) ± standard error. Different letters indicate the values are significantly different (*p* < 0.05). Means were analyzed for finding the significant differences among treatments by using one-way analysis of variance (ANOVA) followed by Duncan’s multiple range test (DMRT).

The transcript accumulations of *ABA receptor* genes (*PYL4*, *PYL8*, and *PYR1*), which are known as core regulators of the ABA signaling pathway, were determined. The results showed that, under control conditions, the expression level of *PYL4* for the combined GA_3_ and Si-treated plants was significantly down-regulated, followed by that of the sole GA_3_-treated, Si-treated, and non-treated plants (Figure 6A). Heat-induced stress significantly upregulated the expression level of PYL4 in the plants. However, the combined GA_3_-Si treated plants demonstrated the significantly reduced transcript accumulation of *PYL4* under heat stress at levels 4.5, 1.75, and 2.08 times less than those of the non-treated and the sole Si and GA_3_-treated plants, respectively. The relative expression of PYL8 and *PYR1* was significantly upregulated in non-treated plants under heat stress, whereas the combined GA_3_ and Si-treated plants exhibited significant down-regulation (Figure 6B,C).

The heat shock transcription factors are well known to be involved in the activation of stress-responsive genes for overcoming abiotic stresses, including heat-induced stress. In the current study, we analyzed the transcript accumulation of the *heat stress transcription factor A−5−like* gene (HSTF-A5), which exhibited the significant up-regulation in the combined GA_3_ and Si-treated plants and significantly lowered expression in non-treated plants under the control condition. The relative expression of *HSTF-A5* was significantly increased under heat stress with all treatments when compared to the control condition. However, the transcript accumulation of *HSTF-A5* in non-treated plants was significantly lowered by 1.7, 1.16, and 1.19 times as compared to those of the combined treatment plants, sole GA_3_-treated plant, and sole Si-treated plants, respectively (Figure 6D). Similarly, the *heat stress transcription factors A−3* (*HSTF*-*A3*) was significantly up-regulated under control conditions in the combined GA_3_ and Si-treated plants as well as in sole Si-treated plants, while the non-treated plants and sole GA_3_-treated plants equally exhibited the least expression (Figure 6E). Under heat stress, the *HSTF-A*3 expression level was enhanced in all treatments, while the combined GA_3_ and Si-treated plants demonstrated significantly higher expression, followed by the sole GA_3_-treated plants, sole Si-treated plants, and non-treated plants, respectively. In connection to this, the relative expression of *HSF30* was significantly higher in the sole GA_3_-treated plants under the control condition (Figure 6F). However, under stress condition, GA_3_ and Si-treated plants displayed significantly higher expression, followed by sole Si-treated, sole GA_3_-treated, and non-treated plants.

## 4. Discussion

Exposure to high temperature limits plant growth and productivity by hampering morphological, biochemical, and physiological processes, in addition to favoring oxidative damage. In the current study, we observed the detrimental impact of high temperature on date palm seedlings, caused by the degradation of plant growth attributes, such as height, biomass, and chlorophyll content. The impact of exogenous application of GA_3_ was more efficient in mitigating high temperature stress in date palm by significantly improving plant height and fresh, dry biomass weight when compared to Si application. However, the application of GA_3_ coupled with Si markedly improved plant growth attributes and significantly minimized the adverse effects of high temperature when compared to their individual application. Such enhancement in the growth attributes of date palm seedlings under high temperature may be correlated with the combined effect of GA_3_ and Si on positive regulation of chlorophyll content (*a* and *b*) and carotenoids augmentation under high temperature. The water status of the plant is considered to be vital under high temperature conditions, as the loss of water content in plant tissues is induced by high temperature stress and subsequently leads to reduced plant growth [32]. In the current study, GA_3_-treated plants exhibited higher relative water status when compared to Si-treated plants. However, their combined application significantly boosted the water status to nearly the same level as that of the control plants. This suggests that combined application of GA_3_ and Si mitigated the adverse effects of high temperature; therefore, the plant leaves were able to hold higher water content for better growth and development. Previously, Luyckx, et al. [33] and Doaigey, et al. [34] reported that the application of silicon and GA_3_ to date plam can lead to the to the development of a cuticle double layer under the leaf epidermis, which subsequently prevents water loss via transpiration under stress conditions. Moreover, the application of GA_3_ and Si to plants is also reported for improving relative water content to induce better growth under hostile conditions [33,34].

The disruption of chlorophyll and the inhibition of photosynthetic activity due to high temperature stress can lead to the generation of a variety of ROS in the chloroplast [35]. The current findings indicated that the sole application of Si markedly alleviated heat-induced oxidative stress as compared to sole application of GA_3_. However, the combined application of GA_3_ and Si significantly reduced the level of O_2_^•−^ and that of MDA, which is known as the end product of lipid peroxidation in date palm, suggesting a strong protective role for their combined application against oxidative stress. The significant mitigation of O_2_^•−^ and MDA content can be ascribed to the stress inhibitory effects of combined Si and GA_3_ application, resulting in the upregulation of antioxidant defense system to encounter oxidative stress [36,37]. Plants have evolved a system of enzymatic and non-enzymatic antioxidants for managing ROS and thereby preventing oxidative stress damage. However, longer exposure and greater severity of heat stress have devastating effects on the antioxidant defense system of plants. On the contrary, Si application triggered antioxidant activities in date palm, while the addition of GA_3_ in the presence of Si further elevated the antioxidative activities (CAT, PPO, POD, and APX). Such enhancement of enzymatic antioxidants can be linked to the scavenging of ROS and, therefore, a lower lipid peroxidation (MDA) and O_2_^•−^ level was observed in combined Si and GA_3_-treated plants under stress conditions. This suggests that the combined application of Si and GA_3_ to date palm is more efficient at imparting thermotolerance to date palm by augmenting their antioxidant defense system. Moreover, along with the up-regulation of APX activity, the transcription level of GPX2 was also significantly enhanced by the combined application of Si and GA_3_. This indicates that combined Si and GA_3_ treatment simultaneously activated APX and GPX2 in preparation for the ROS encounter by suppressing toxic H_2_O_2_ levels in plants under heat stress [38].

Heat stress can also lead to the disruption of *GAPDH* activity, which is crucial for carbon flux in the Calvin cycle and for regulating the carbon assimilation and photosynthesis rates. *GAPDH* aids in converting glycerate-3-phosphate to glyceraldehyde-3-phosphate through interactions with NADPH. This inhibits the ROS-induced breakdown of the photosystem II repair cycle by reducing ROS production, subsequently maintaining photosynthetic efficiency [39,40]. In the current study, the transcript accumulation of the *NADP-dependent GAPDH* gene was markedly reduced in only the heat stressed plants, whereas the combined application of Si and GA_3_ led to significant expression of the gene under heat stress. The significant transcript accumulation of *GAPDH* implies that GA_3_ and Si collectively provided ample energy to the date palm under heat stress for regulating ROS-induced metabolic responses for cellular adjustment by routing carbon away from glycerol and subsequently leading to glycolysis and ATP formation [41]. Moreover, the simultaneous co-expression of Cyt-Cu/Zn SOD and CAT indicate that the combined application of Si and GA3 efficiently enhanced the capability of date palm to cope with the heat-induced oxidative stress.

Plant hormone metabolism is closely interlinked with the plant abiotic stress coping potential. The findings from the current study illustrated that the endogenous ABA content of date palm is enhanced in response to heat stress. However, the combined application of Si and GA_3_ drastically lowered the level of ABA accumulation in date palm under heat stress. The lower regulation of ABA following the combined application of Si and GA_3_ might be correlated to the beneficial impact of GA_3_, which leads to better photosynthetic activity, stomatal regulation, and gas exchange, as well as the capability of Si to boost the antioxidant defense system. The synergist effects result in less ROS accumulation and a corresponding decrease in the level of ABA in the date palm. In line with this theory, the transcript levels of the ABA signaling-related genes (*PYL4*, *PYL8*, and *PYR1*) were down-regulated in the combined Si and GA_3_-treated plants under heat stress; therefore, a lower accumulation of ABA was recorded. These findings further highlight the effective role of the combined application of Si and GA_3_ in ameliorating heat-induced stress in date palm. SA is a signaling molecule that is known to mitigate the adverse effects of heat stress in plants by regulating various physiological and biochemical processes to provide both basal and acquired thermotolerance [42]. In the current study, the combined application of Si and GA_3_ resulted in higher accumulation of SA in date palm and successfully alleviated the adverse effects of heat stress. The higher accumulation of endogenous SA is reported to activate proline biosynthesis for the augmentation of osmotic potential, enabling plants to uptake water, and triggers antioxidant enzymes under heat stress to impart thermotolerance to plants [43]. The combined treatment of Si and GA_3_ resulted in an antagonistic interaction of SA with ABA, which suggests that ABA signaling alleviates heat stress-induced leaf senescence, chlorophyll degradation, and redox modulation [44]. Plant heat shock transcription factors are known to participate in heat stress-related hormonal signaling pathways, such as SA. The activation of heat shock transcription factor genes, such as *Hsf3*, can boost plant defense against hostile conditions via the modulation of endogenous accumulation and signaling [42,45]. Therefore, the higher accumulation of endogenous SA in date palm under heat stress could be ascribed to the significant transcript accumulation of heat shock transcription factors *Hsf3*, *HsfA5*, and *Hsf30* via the interactive effects of exogenous Si and GA_3_ application, providing tolerance against heat stress.

Heat shock transcription factors are known to regulate the expression of HSPs to maintain homeostasis in plants against different stresses, including heat and chemical stresses [46]. The expression of HSPs by *Hsfs* genes is regulated via their interactions with a palindromic binding motif in the promoter region of heat-responsive genes, such as heat shock elements to counteract heat stress-induced ROS [47]. We found that the combined application of Si and GA_3_ significantly triggered transcript accumulation of *HsfA3*, *HsfA5*, and *Hsf30*, suggesting that they play a heat stress ameliorative role in date palm by imparting protection from heat-induced ROS generation and boosting the antioxidative response. Therefore, the enhancement of the antioxidative activities (CAT, POD, PPO, and APX) and the expression of the corresponding genes can be correlated with the significant activation of *Hsfs* genes by the combined application of Si and GA3 in response to heat-induced stress. However, further transcriptomic-based studies are required in order to uncover the underlying mechanism behind the heat shock transcription factors of date palm by investigating the interactive effects of GA_3_ and Si under heat stress.

## 5. Conclusions

In conclusion, the combined application of Si and GA_3_ to the date palm successfully mitigated the adverse effects of heat stress in plants, directly improved plant growth and development, and induced physiological and biochemical modulation. Taken together, our data revealed that, when compared to sole application, combined application of GA_3_ and Si significantly protected date palm plants from heat-induced stress. The effects of exogenous GA and Si significantly activated the heat shock transcription factors genes, particularly *HsfA3*, and the anti-oxidative system of date palm by up-regulating the *GPX2*, *Cyt-Cu/Zn SOD*, and *CAT* expression levels. Moreover, the interactive effects of GA and Si influenced the cross-talk between stress-related endogenous hormones (ABA and SA) by reducing endogenous ABA accumulation and down-regulating ABA signaling-related genes (*PYL4*, *PYL8*, and *PYR1*), which subsequently induced the antagonistic effects of SA. Therefore, the combined application of Si and GA_3_ is efficient in enhancing date palm growth and development under heat stress condition.

## Figures and Tables

**Table 1:** The gene name, description, product size, reference number, and oligonucleotide sequences used for qRT-PCR.

Gene Name	Description	Primer Sequence (5′–3′)	Size (bp)	Accession
*GAPDH*	NADP-dependentglyceraldehyde-3-phosphatedehydrogenase	F: TTTGGACCAGTCTTGCCAGTAA R: TGCAGTGATGGATACCTTCTTCA	61	XM_008801419.1
*Cyt-Cu/Zn SOD*	superoxide dismutase [Cu-Zn]-like	F: AAGCCTCTCTGGCCTCGAA R: CACCGAGGGCATGAACATG	56	XM_008813474.1
*GPX2*	glutathione peroxidase	F: GGAAGAACGCTGCACCCCTAT R: GCTCCATGACCTTGCCATCTTT	120	XP_008790151.1
*CAT*	Catalase	F:TTCTTCTCACACCACCCAGAG R: GTTCACGCCAAAACCATCCA	102	XP_026656046.1
*PYL8*	abscisic acid receptor PYL8-like	F: CAGCACCGAAAGGTTGGAGTTT R: GATGGAGGGTAATGATGGAGGA	110	XM_008791563.3
*PYL4*	abscisic acid receptor PYL4-like	F: CGTCGAGTCCTACGTTGTCG R: GCCAGGTTCTCGGAGGTATG	120	XM_008801643.3
*PYR1*	abscisic acid receptor PYR1	F: ACGGTGGTGCTGGAATCGTA R: GAGGCGAGCTTCTGGAGGTT	110	NW_008246541.1
*HSTF-A5*	heat stress transcription factor A-5-like	F: CTCCTCCCCGCCTACTTCAA R: GCGAACTCCCATCTCTCTGGA	101	XP_017700691.1
*HSF30*	heat shock factor protein HSF30-like	F: CGACGAAACATCTCCCAGAGC R: GCAGTCCCTCCTCAATCTATCAAC	108	XP_008775152.1
*HSTF-A3*	heat stress transcription factor A-3	F: GCCGTCAAGGTGGAGCTTCTA R: CATCCGAAAACATCCTCTCTGG	112	XP_008807524.1
*Act*	Actin	F: TCAATGTGCCTGCCATGTATGT R: GCGGCCGCTAGCATAGAG	62	XM_008778129

## Supplementary Materials

The following are available online at https://www.mdpi.com/2223-7747/9/5/620/s1, details of statistical analysis for Figure 1, Figure 2, Figure 3, Figure 4, Figure 5 and Figure 6 are available in the Supplementary Information file.
