# Supplementary material for: Silicon and Gibberellins: Synergistic Function in Harnessing ABA Signaling and Heat Stress Tolerance in Date Palm (Phoenix dactylifera L.)
Source: Plants (Basel). 2020 May 13;9(5):620. doi: 10.3390/plants9050620 (PMC7285242; doi:10.3390/plants9050620)
Supplement: Supplementary file 1 [file plants-09-00620-s001.pdf]

onion day

The ANOVA Procedure

Class Level Information		
Class	Levels	Values
v	8	a b c d e f g h

Number of Observations Read	24
Number of Observations Used	24

Figure 1A

onion day

The ANOVA Procedure

Dependent Variable: y

Source	DF	Sum of Squares	Mean Square	F Value	Pr > F
Model	7	627.8033167	89.6861881	82.03	<.0001
Error	16	17.4940667	1.0933792		
Corrected Total	23	645.2973833			

R-Square	Coeff Var	Root MSE	y Mean
0.972890	3.691499	1.045648	28.32583

Source	DF	Anova SS	Mean Square	F Value	Pr > F
v	7	627.8033167	89.6861881	82.03	<.0001

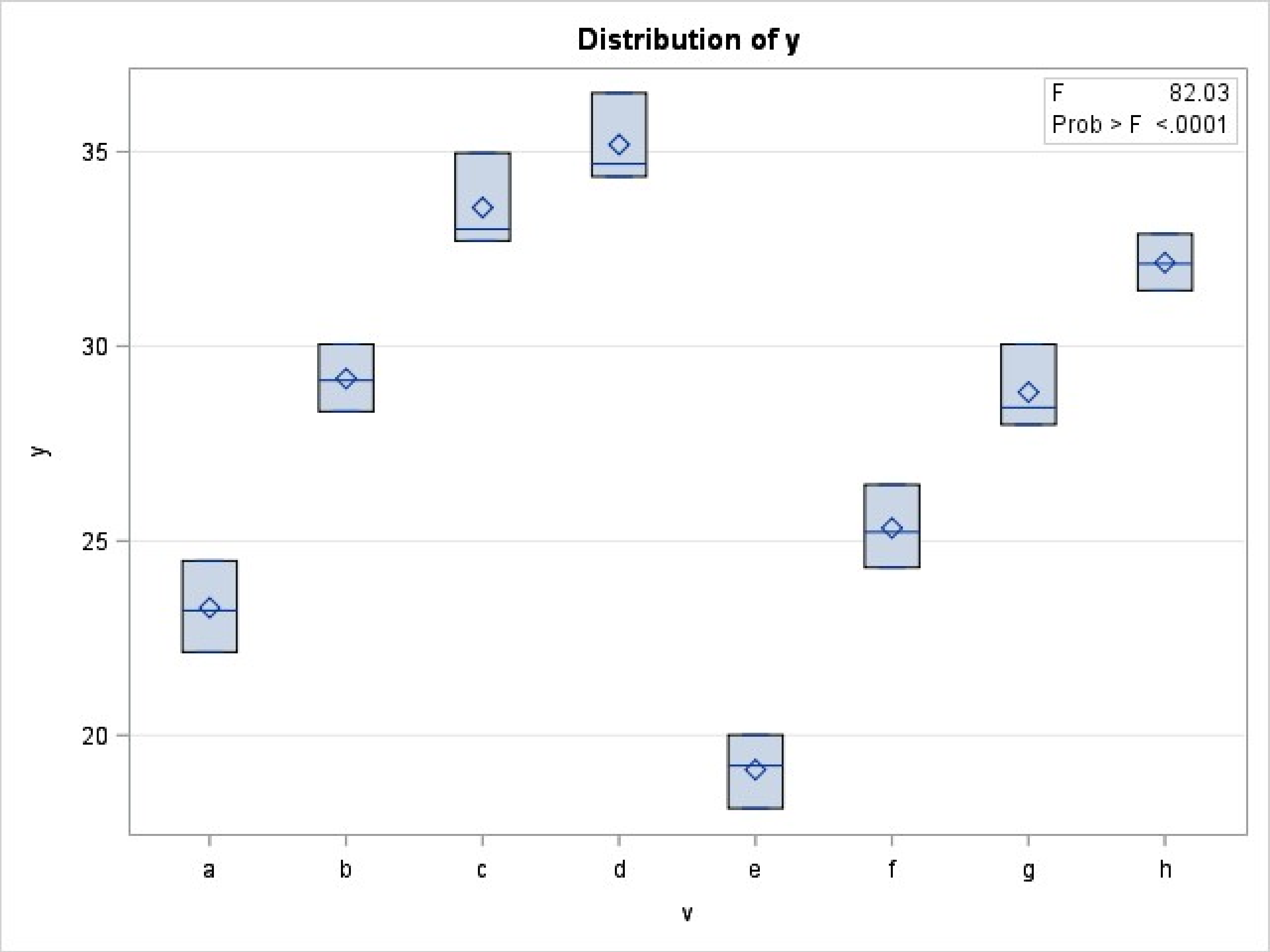

onion day

The ANOVA Procedure

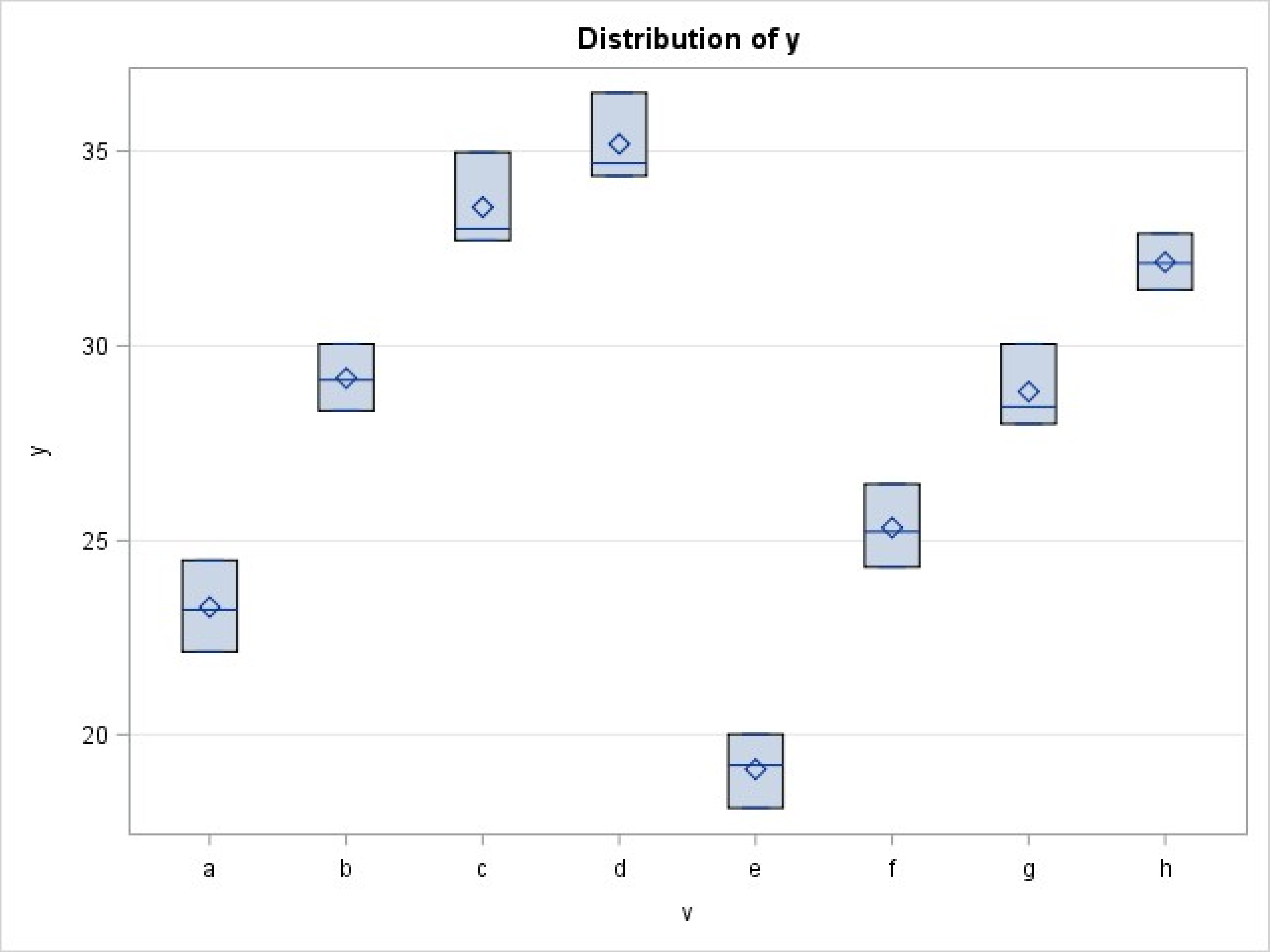

onion day

The ANOVA Procedure

Duncan's Multiple Range Test for y

Note: This test controls the Type I comparisonwise error rate, not the experimentwise error rate.

**Alpha**	0.05
**Error Degrees of Freedom**	16
**Error Mean Square**	1.093379

**Number of Means**	**2**	**3**	**4**	**5**	**6**	**7**	**8**
**Critical Range**	1.810	1.898	1.953	1.991	2.018	2.038	2.054

**Means with the same letter are not significantly different.**				
**Duncan Grouping**		**Mean**	**N**	**v**
	A	35.1867	10	d
	A			
B	A	33.5633	10	c
B				
B		32.1467	10	h
	C	29.1633	10	b
	C			
	C	28.8167	10	g
	D	25.3333	10	f
	E	23.2800	10	a
	F	19.1167	10	e

V describes the treatments.

a; Control b; Si c; GA3 d; Si+GA3 e; Heat f; Si+Heat g; GA3+Heat h; Si+GA3+Heat

Figure 1B

onion day

The ANOVA Procedure

Class Level Information		
Class	Levels	Values
v	8	a b c d e f g h

Number of Observations Read	24
Number of Observations Used	24

onion day

The ANOVA Procedure

Dependent Variable: y

Source	DF	Sum of Squares	Mean Square	F Value	Pr > F
Model	7	203.1836958	29.0262423	105.57	<.0001
Error	16	4.3992000	0.2749500		
Corrected Total	23	207.5828958			

R-Square	Coeff Var	Root MSE	y Mean
0.978808	5.000025	0.524357	10.48708

Source	DF	Anova SS	Mean Square	F Value	Pr > F
v	7	203.1836958	29.0262423	105.57	<.0001

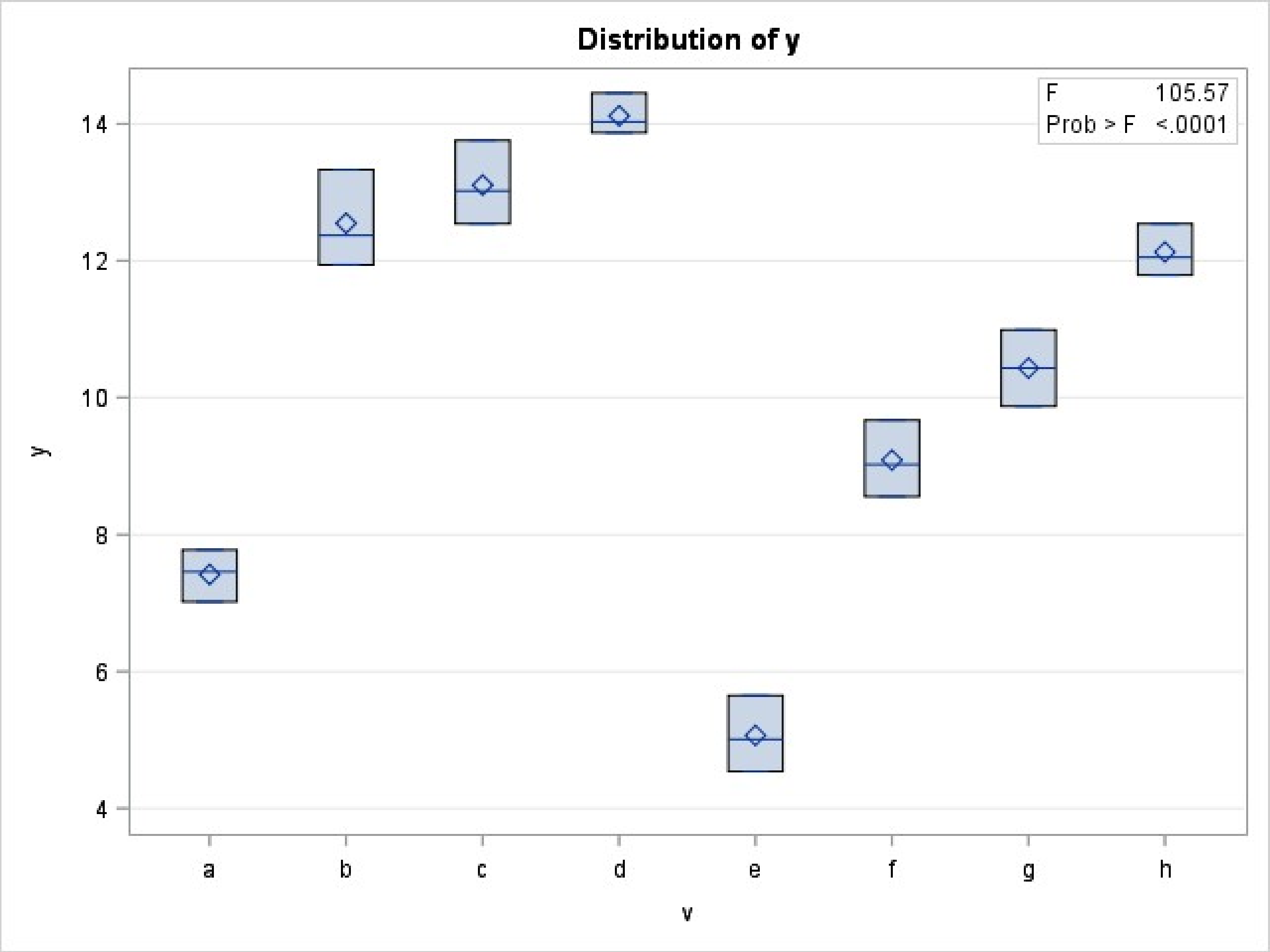

onion day

The ANOVA Procedure

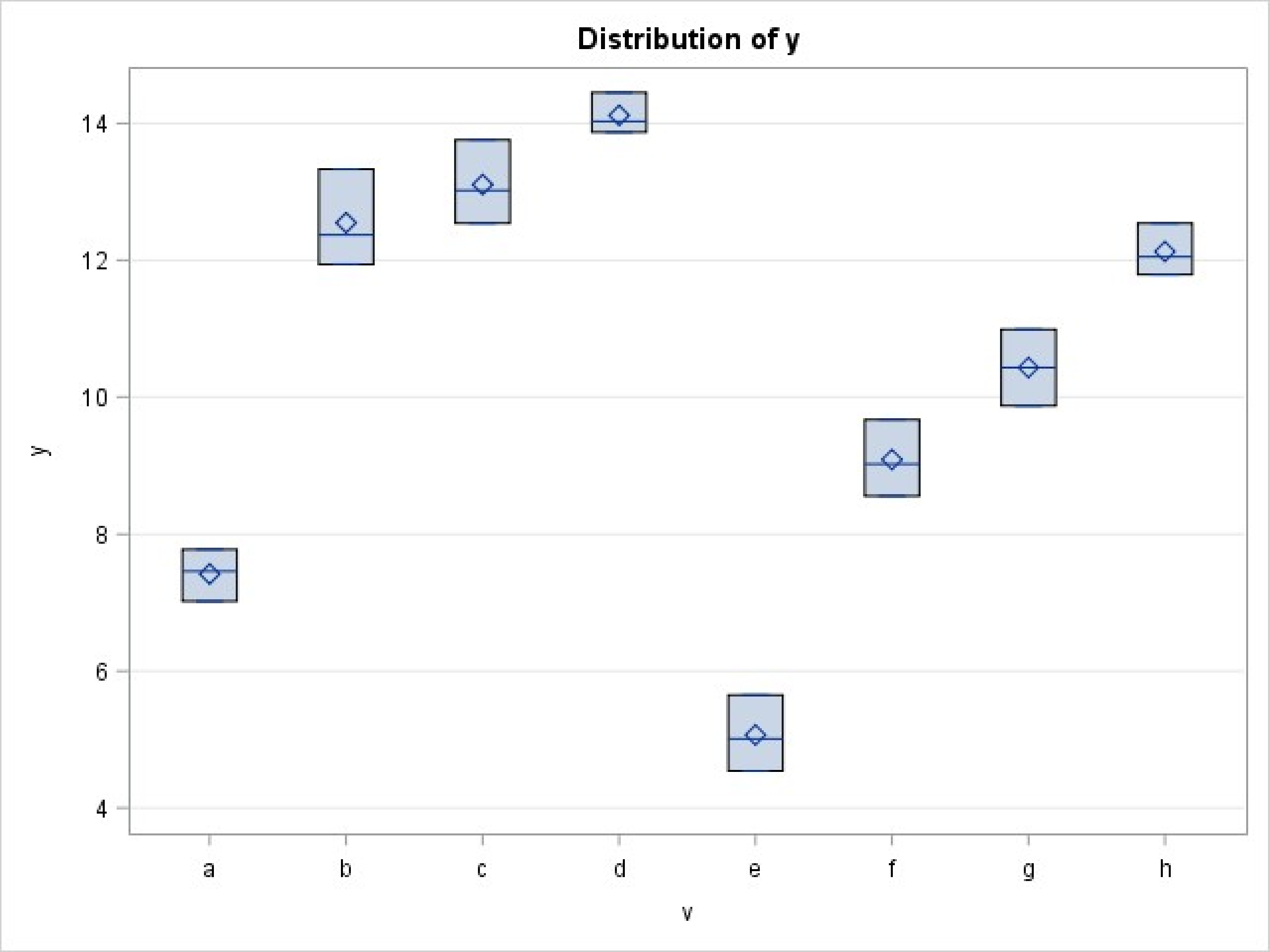

onion day

The ANOVA Procedure

Duncan's Multiple Range Test for y

Note: This test controls the Type I comparisonwise error rate, not the experimentwise error rate.

**Alpha**	0.05
**Error Degrees of Freedom**	16
**Error Mean Square**	0.27495

**Number of Means**	**2**	**3**	**4**	**5**	**6**	**7**	**8**
**Critical Range**	0.908	0.952	0.979	0.998	1.012	1.022	1.030

Means with the same letter are not significantly different.

**Duncan Grouping**		**Mean**	**N**	**v**
	A	14.1167	10	d
	B	13.1067	10	c
	B			
C	B	12.5467	10	b
C				
C		12.1267	10	h
	D	10.4300	10	g
	E	9.0833	10	f
	F	7.4200	10	a
	G	5.0667	10	e

V describes the treatments.

a; Control b; Si c; GA3 d; Si+GA3 e; Heat f; Si+Heat g; GA3+Heat h; Si+GA3+Heat

Figure 1C

onion day

The ANOVA Procedure

Class Level Information		
Class	Levels	Values
v	8	a b c d e f g h

Number of Observations Read	24
Number of Observations Used	24

onion day

The ANOVA Procedure

Dependent Variable: y

Source	DF	Sum of Squares	Mean Square	F Value	Pr > F
Model	7	101282.0079	14468.8583	364.37	<.0001
Error	16	635.3559	39.7097		
Corrected Total	23	101917.3638			

R-Square	Coeff Var	Root MSE	y Mean
0.993766	3.082129	6.301567	204.4550

Source	DF	Anova SS	Mean Square	F Value	Pr > F
v	7	101282.0079	14468.8583	364.37	<.0001

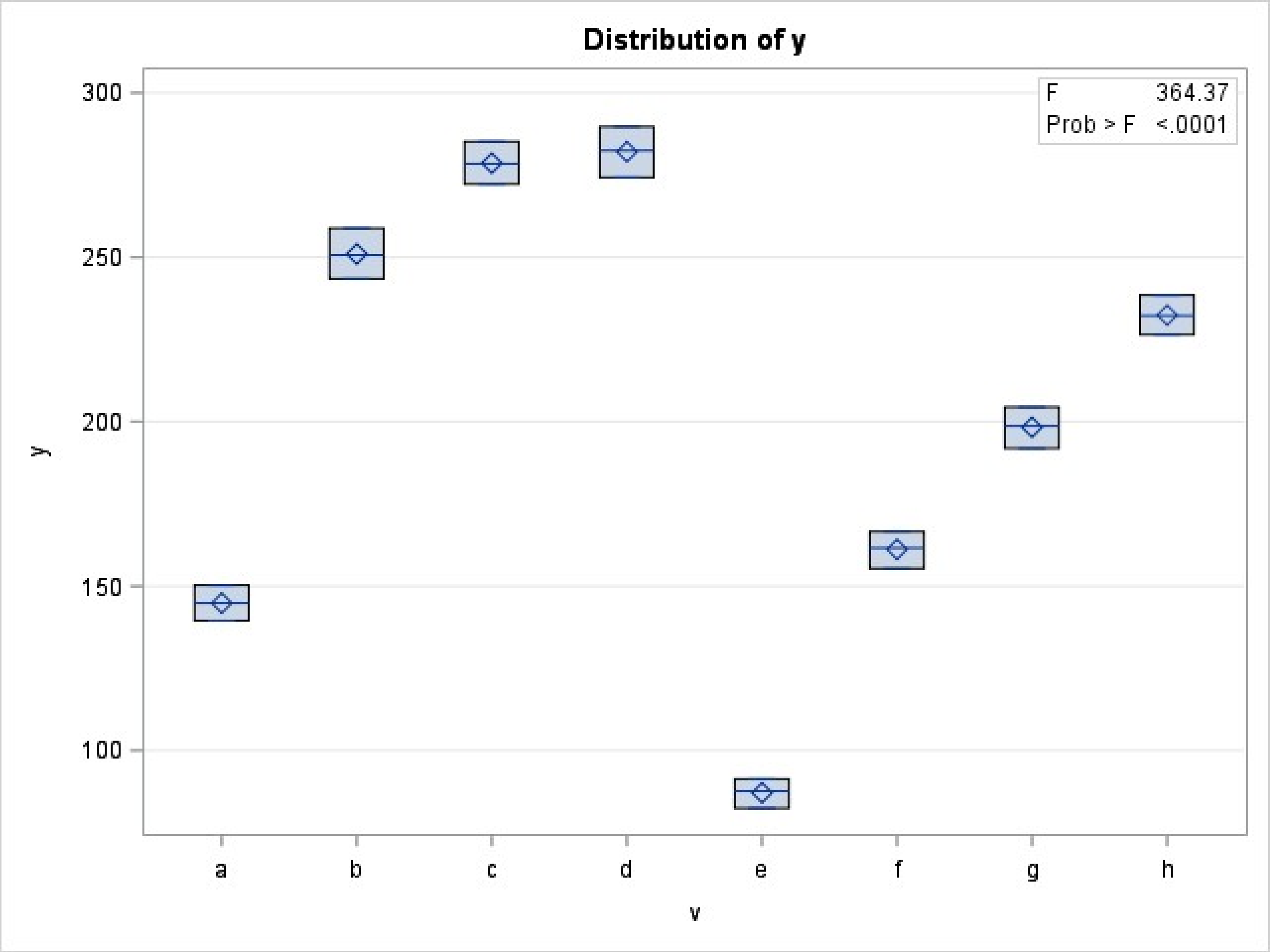

onion day

The ANOVA Procedure

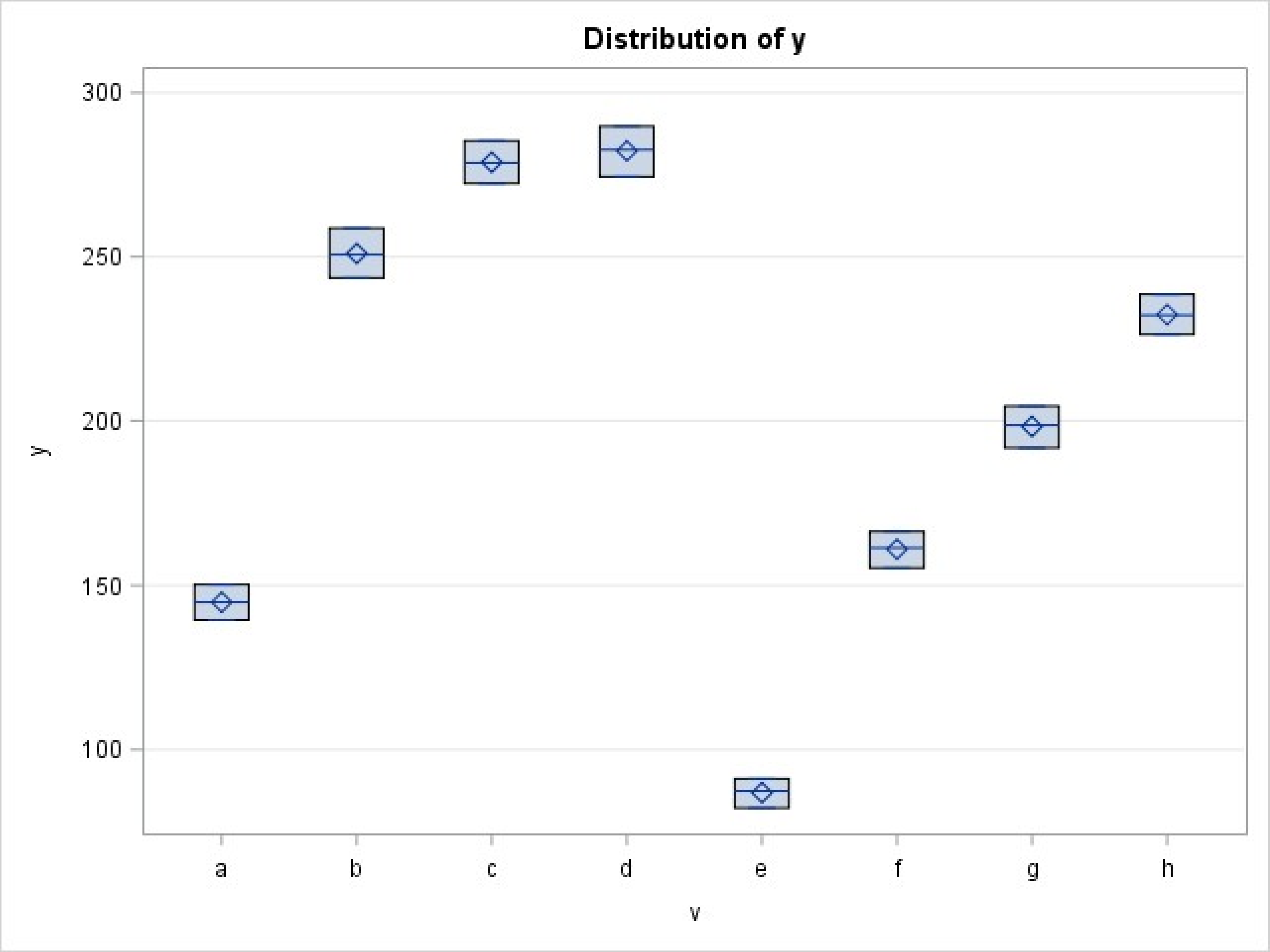

onion day

The ANOVA Procedure

Duncan's Multiple Range Test for y

Note: This test controls the Type I comparisonwise error rate, not the experimentwise error rate.

**Alpha**	0.05
**Error Degrees of Freedom**	16
**Error Mean Square**	39.70975

**Number of Means**	**2**	**3**	**4**	**5**	**6**	**7**	**8**
**Critical Range**	10.91	11.44	11.77	12.00	12.16	12.28	12.38

**Means with the same letter are not significantly different.**			
**Duncan Grouping**	**Mean**	**N**	**v**
A	282.220	10	d
A			
A	278.713	10	c
B	250.997	10	b
C	232.367	10	h
D	198.287	10	g
E	161.147	10	f
F	144.900	10	a
G	87.010	10	e

V describes the treatments.

a; Control b; Si c; GA3 d; Si+GA3 e; Heat f; Si+Heat g; GA3+Heat h; Si+GA3+Heat

Figure 1D

onion day

The ANOVA Procedure

Class Level Information		
Class	Levels	Values
v	8	a b c d e f g h

Number of Observations Read	24
Number of Observations Used	24

onion day

The ANOVA Procedure

Dependent Variable: y

Source	DF	Sum of Squares	Mean Square	F Value	Pr > F
Model	7	69460.89912	9922.98559	413.45	<.0001
Error	16	384.00407	24.00025		
Corrected Total	23	69844.90318			

R-Square	Coeff Var	Root MSE	y Mean
0.994502	3.115045	4.899005	157.2692

Source	DF	Anova SS	Mean Square	F Value	Pr > F
v	7	69460.89912	9922.98559	413.45	<.0001

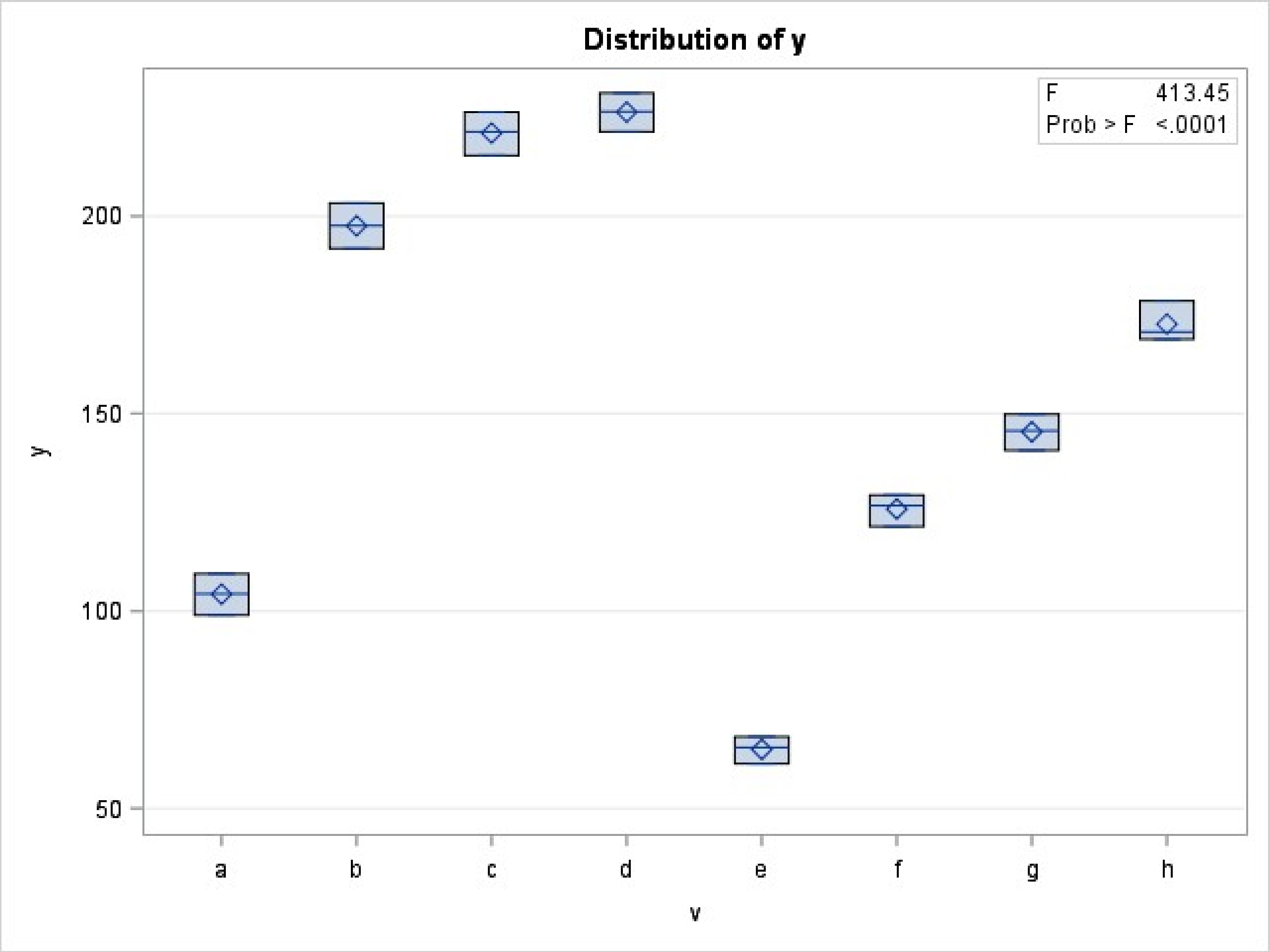

onion day

The ANOVA Procedure

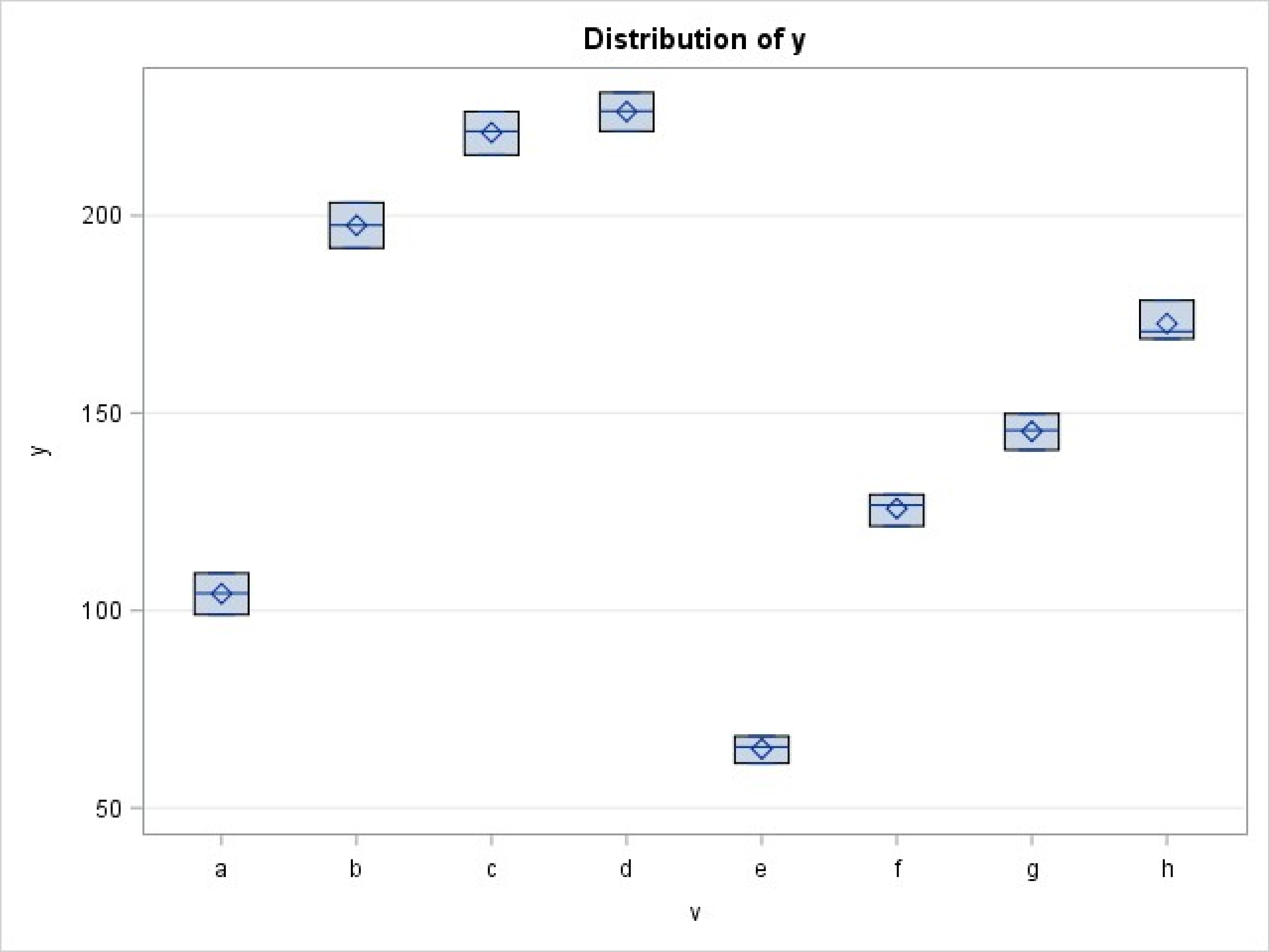

onion day

The ANOVA Procedure

Duncan's Multiple Range Test for y

Note: This test controls the Type I comparisonwise error rate, not the experimentwise error rate.

**Alpha**	0.05
**Error Degrees of Freedom**	16
**Error Mean Square**	24.00025

**Number of Means**	**2**	**3**	**4**	**5**	**6**	**7**	**8**
**Critical Range**	8.479	8.892	9.150	9.327	9.454	9.550	9.622

**Means with the same letter are not significantly different.**			
**Duncan Grouping**	**Mean**	**N**	**v**
A	226.367	10	d
A			
A	221.067	10	c
B	197.560	10	b
C	172.657	10	h
D	145.333	10	g
E	125.880	10	f
F	104.300	10	a
G	64.990	10	e

V describes the treatments.

a; Control b; Si c; GA3 d; Si+GA3 e; Heat f; Si+Heat g; GA3+Heat h; Si+GA3+Heat

Figure 1E

onion day

The ANOVA Procedure

Class Level Information		
**Class**	**Levels**	**Values**
v	8	a b c d e f g h

**Number of Observations Read**	24
**Number of Observations Used**	24

onion day

The ANOVA Procedure

Dependent Variable: y

Source	DF	Sum of Squares	Mean Square	F Value	Pr > F
Model	7	25218.86800	3602.69543	216.48	<.0001
Error	16	266.27533	16.64221		
Corrected Total	23	25485.14333			

R-Square	Coeff Var	Root MSE	y Mean
0.989552	4.180373	4.079486	97.58667

Source	DF	Anova SS	Mean Square	F Value	Pr > F
v	7	25218.86800	3602.69543	216.48	<.0001

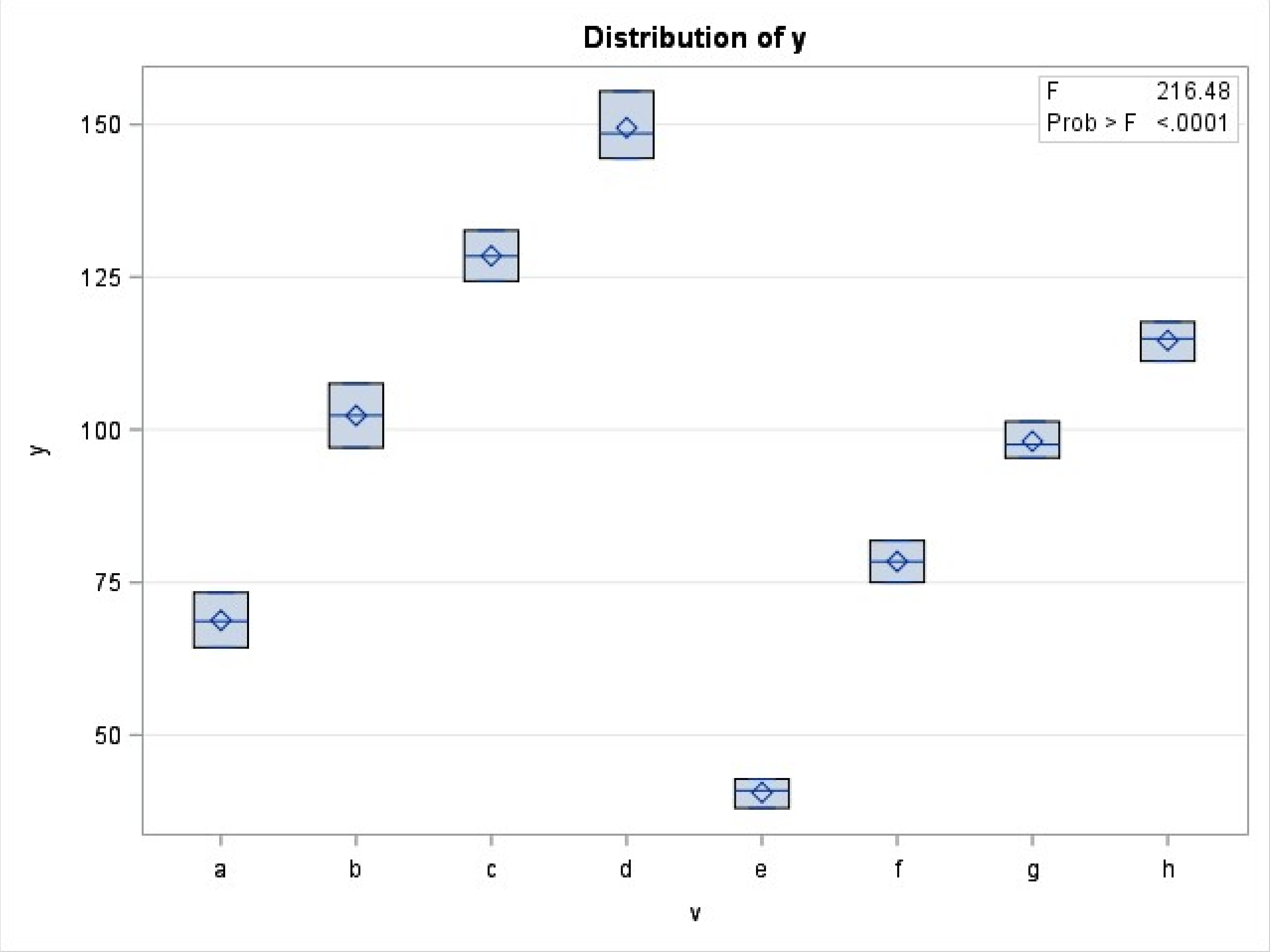

onion day

The ANOVA Procedure

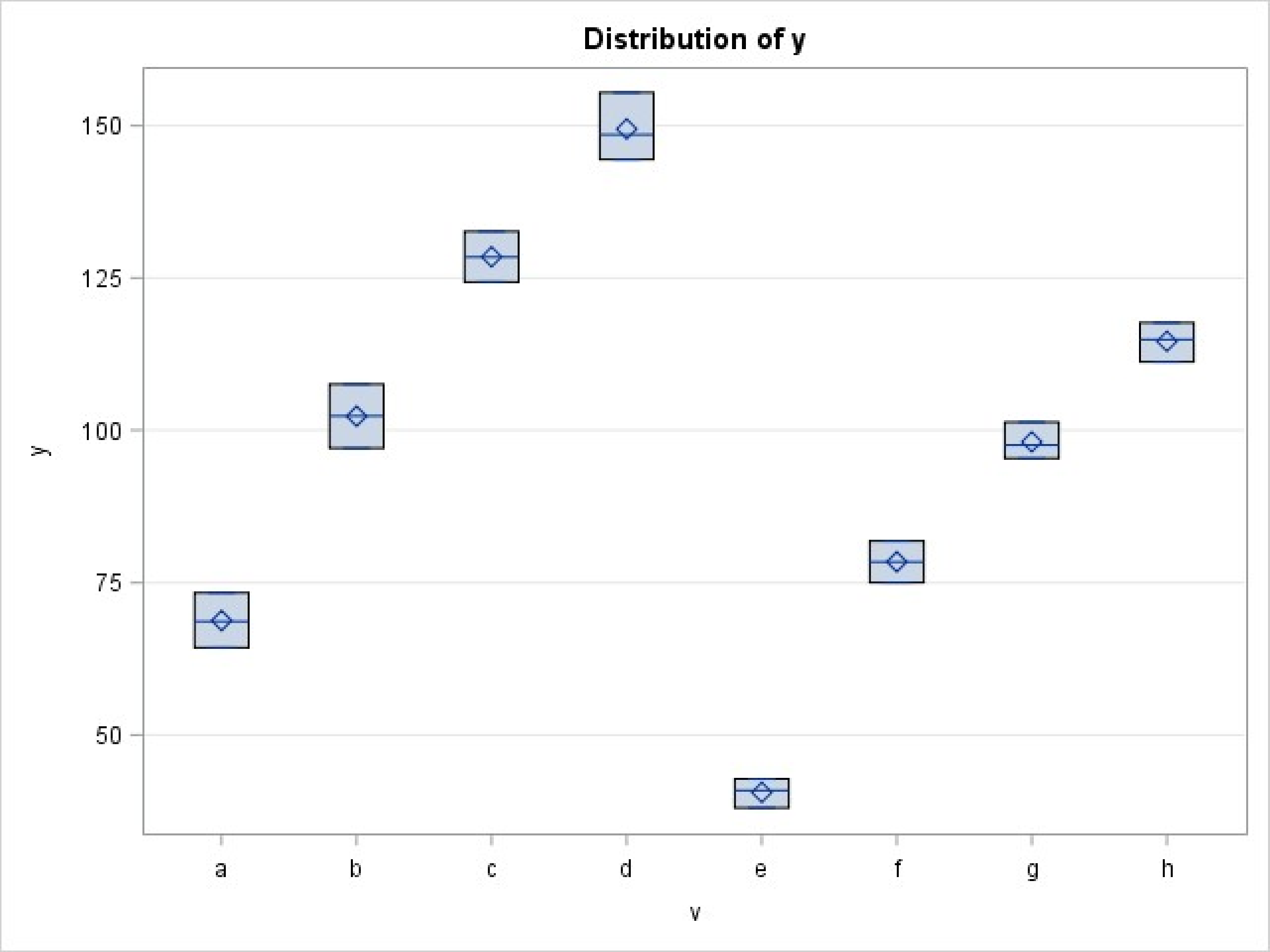

onion day

The ANOVA Procedure

Duncan's Multiple Range Test for y

Note: This test controls the Type I comparisonwise error rate, not the experimentwise error rate.

**Alpha**	0.05
**Error Degrees of Freedom**	16
**Error Mean Square**	16.64221

**Number of Means**	**2**	**3**	**4**	**5**	**6**	**7**	**8**
**Critical Range**	7.061	7.405	7.619	7.766	7.873	7.952	8.013

Means with the same letter are not significantly different.

**Duncan Grouping**	**Mean**	**N**	**v**
A	149.480	10	d
B	128.473	10	c
C	114.590	10	h
D	102.293	10	b
D			
D	98.067	10	g
E	78.450	10	f
F	68.777	10	a
G	40.563	10	e

V describes the treatments.

a; Control b; Si c; GA3 d; Si+GA3 e; Heat f; Si+Heat g; GA3+Heat h; Si+GA3+Heat

Figure 1F

onion day

The ANOVA Procedure

Class Level Information		
Class	Levels	Values
v	8	a b c d e f g h

Number of Observations Read	24
Number of Observations Used	24

onion day

The ANOVA Procedure

Dependent Variable: y

Source	DF	Sum of Squares	Mean Square	F Value	Pr > F
Model	7	4274.653333	610.664762	131.30	<.0001
Error	16	74.415400	4.650962		
Corrected Total	23	4349.068733			

R-Square	Coeff Var	Root MSE	y Mean
0.982889	5.860085	2.156609	36.80167

Source	DF	Anova SS	Mean Square	F Value	Pr > F
v	7	4274.653333	610.664762	131.30	<.0001

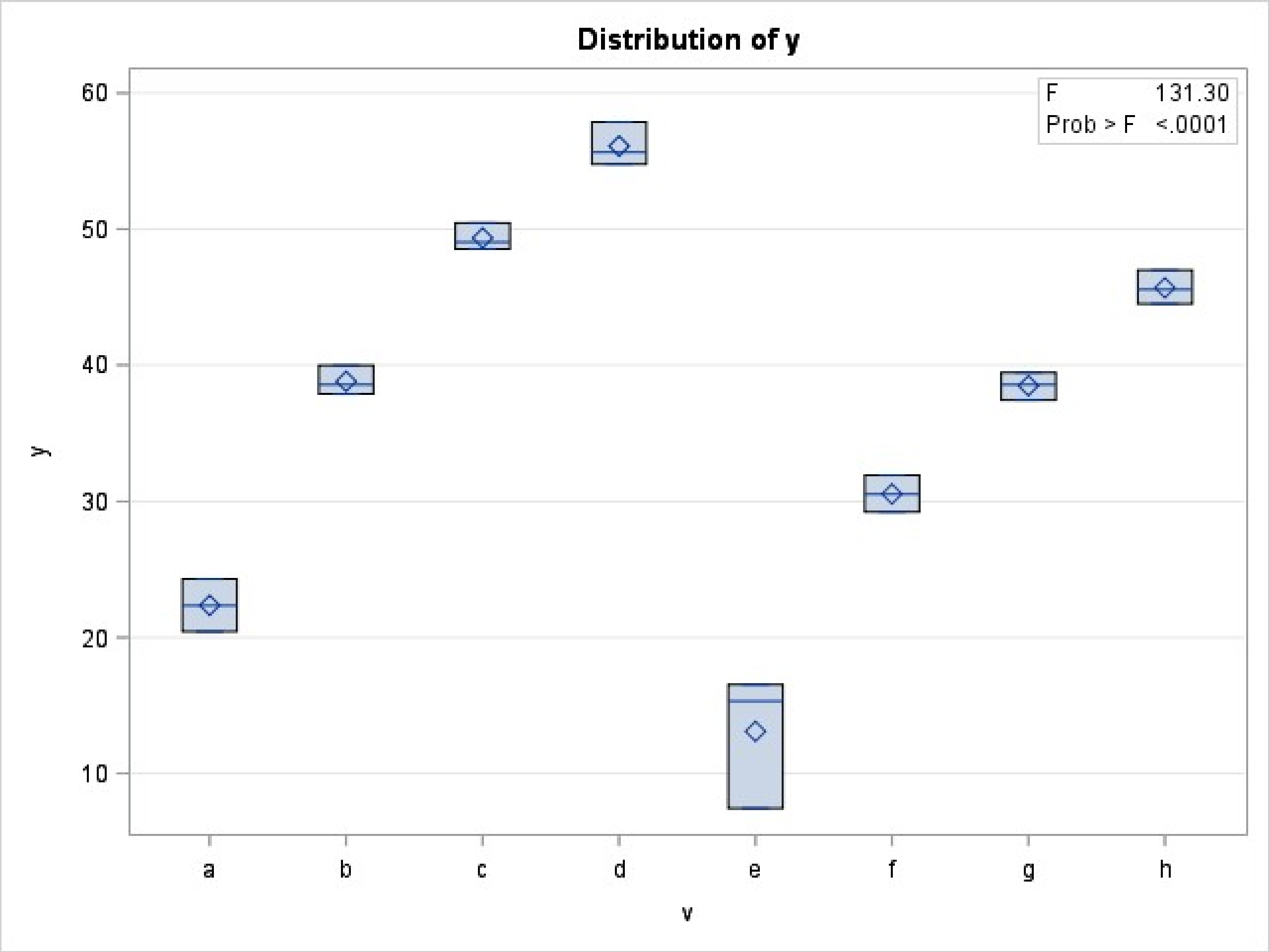

onion day

The ANOVA Procedure

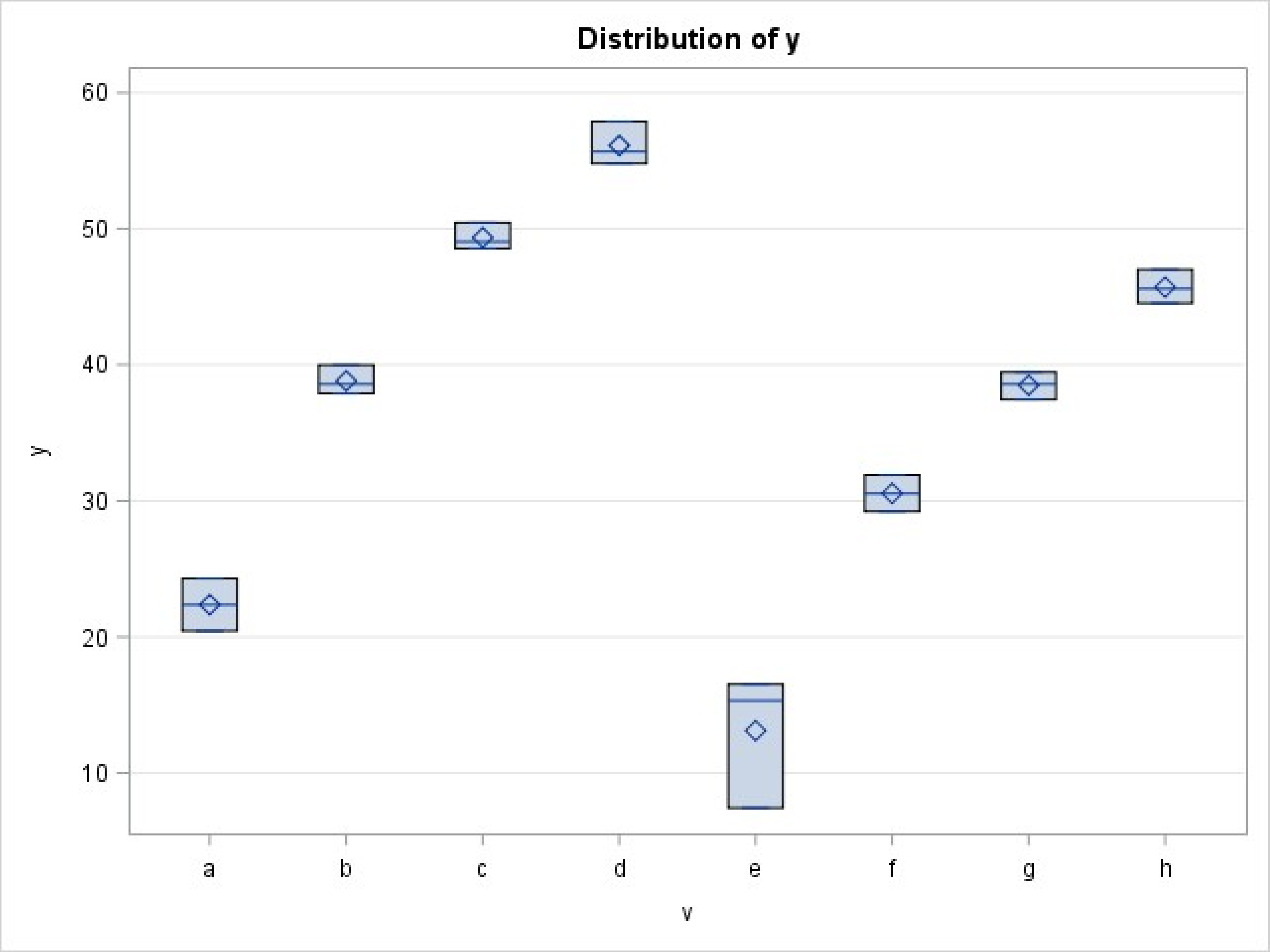

onion day

The ANOVA Procedure

Duncan's Multiple Range Test for y

Note: This test controls the Type I comparisonwise error rate, not the experimentwise error rate.

**Alpha**	0.05
**Error Degrees of Freedom**	16
**Error Mean Square**	4.650962

**Number of Means**	**2**	**3**	**4**	**5**	**6**	**7**	**8**
**Critical Range**	3.733	3.914	4.028	4.106	4.162	4.204	4.236

**Means with the same letter are not significantly different.**			
**Duncan Grouping**	**Mean**	**N**	**v**
A	56.100	10	d
B	49.343	10	c
B			
B	45.680	10	h
C	38.800	10	b
C			
C	38.480	10	g
D	30.553	10	f
E	22.360	10	a
F	13.097	10	e

V describes the treatments.

a; Control b; Si c; GA3 d; Si+GA3 e; Heat f; Si+Heat g; GA3+Heat h; Si+GA3+Heat

onion day

The ANOVA Procedure

Class Level Information		
Class	Levels	Values
v	8	a b c d e f g h

Number of Observations Read	24
Number of Observations Used	24

Figure 2A

onion day

The ANOVA Procedure

Dependent Variable: y

Source	DF	Sum of Squares	Mean Square	F Value	Pr > F
Model	7	40.98212917	5.85458988	59.64	<.0001
Error	16	1.57066667	0.09816667		
Corrected Total	23	42.55279583			

R-Square	Coeff Var	Root MSE	y Mean
0.963089	5.761685	0.313316	5.437917

Source	DF	Anova SS	Mean Square	F Value	Pr > F
v	7	40.98212917	5.85458988	59.64	<.0001

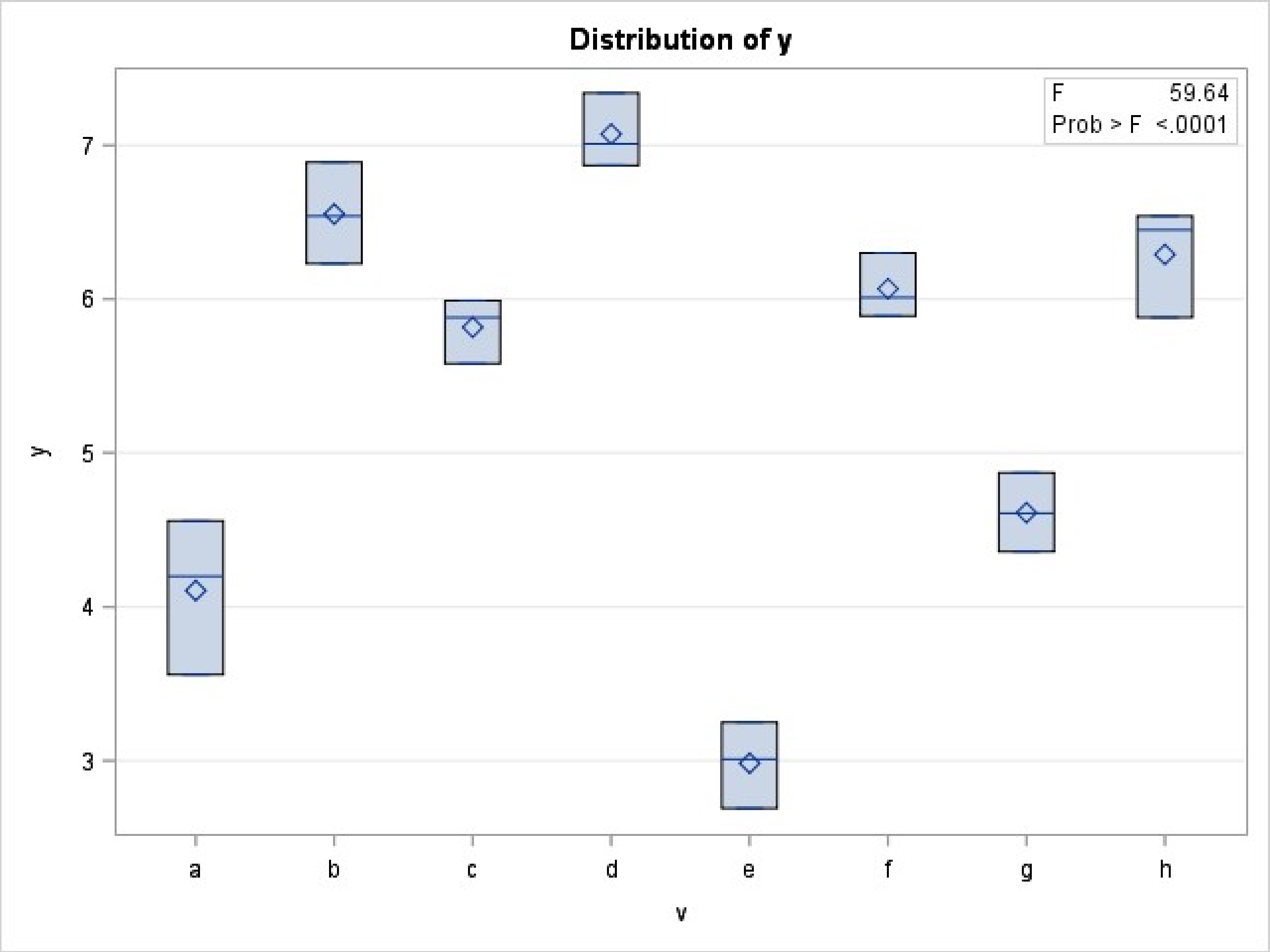

onion day

The ANOVA Procedure

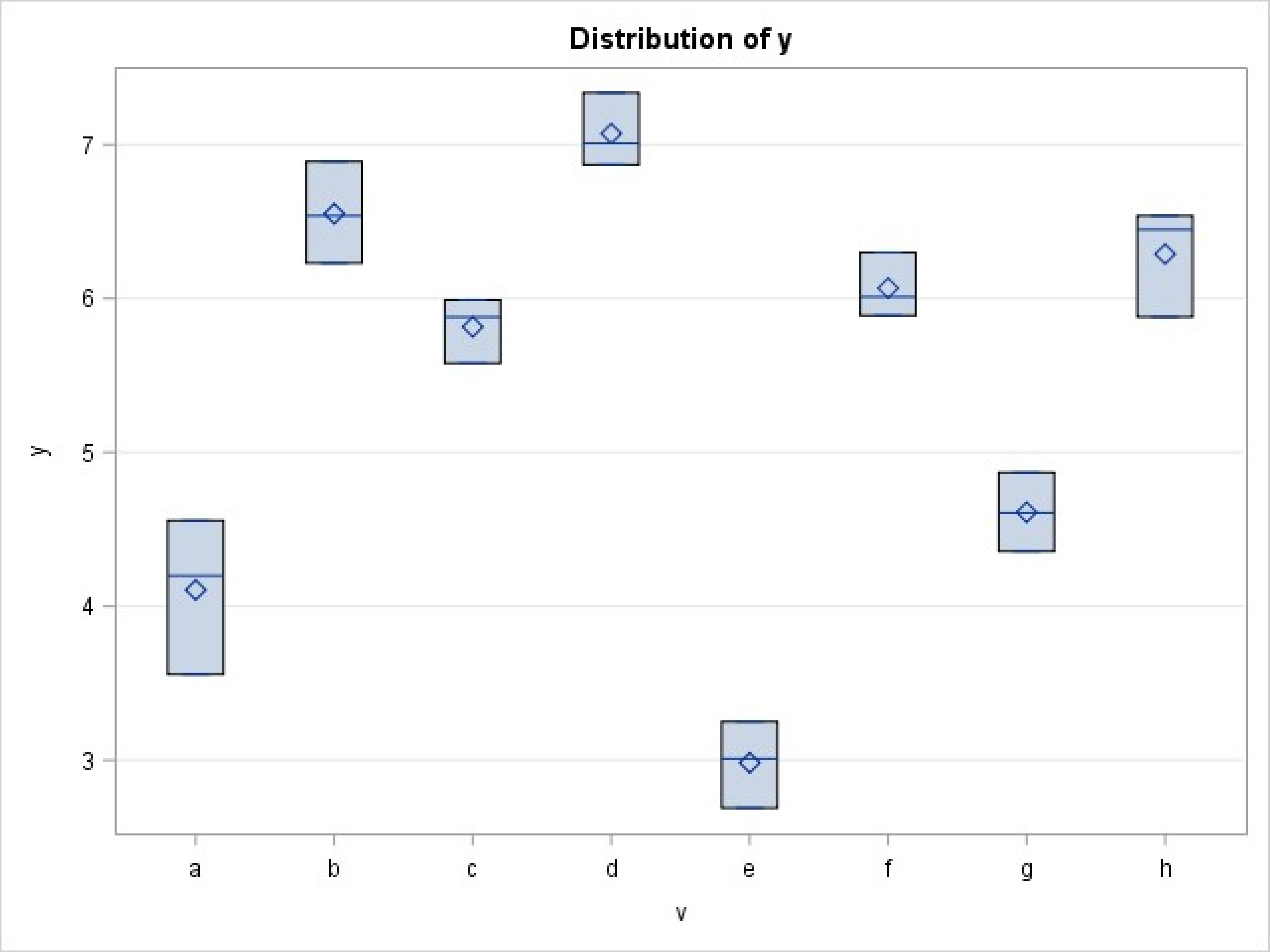

onion day

The ANOVA Procedure

Duncan's Multiple Range Test for y

Note: This test controls the Type I comparisonwise error rate, not the experimentwise error rate.

**Alpha**	0.05
**Error Degrees of Freedom**	16
**Error Mean Square**	0.098167

**Number of Means**	**2**	**3**	**4**	**5**	**6**	**7**	**8**
**Critical Range**	.5423	.5687	.5852	.5965	.6047	.6107	.6154

**Means with the same letter are not significantly different.**				
**Duncan Grouping**		**Mean**	**N**	**v**
	A	7.0733	6	d
	A			
B	A	6.5533	6	b
B				
B	C	6.2900	6	h
B	C			
B	C	6.0667	6	f
	C			
	C	5.8167	6	c
	D	4.6133	6	g
	D			
	D	4.1067	6	a
	E	2.9833	6	e

V describes the treatments.

a; Control b; Si c; GA3 d; Si+GA3 e; Heat f; Si+Heat g; GA3+Heat h; Si+GA3+Heat

Figure 2B

onion day

The ANOVA Procedure

Class Level Information		
Class	Levels	Values
v	8	a b c d e f g h

Number of Observations Read	24
Number of Observations Used	24

onion day

The ANOVA Procedure

Dependent Variable: y

Source	DF	Sum of Squares	Mean Square	F Value	Pr > F
Model	7	1091.395362	155.913623	100.63	<.0001
Error	16	24.791133	1.549446		
Corrected Total	23	1116.186496			

R-Square	Coeff Var	Root MSE	y Mean
0.977789	5.597080	1.244767	22.23958

Source	DF	Anova SS	Mean Square	F Value	Pr > F
v	7	1091.395362	155.913623	100.63	<.0001

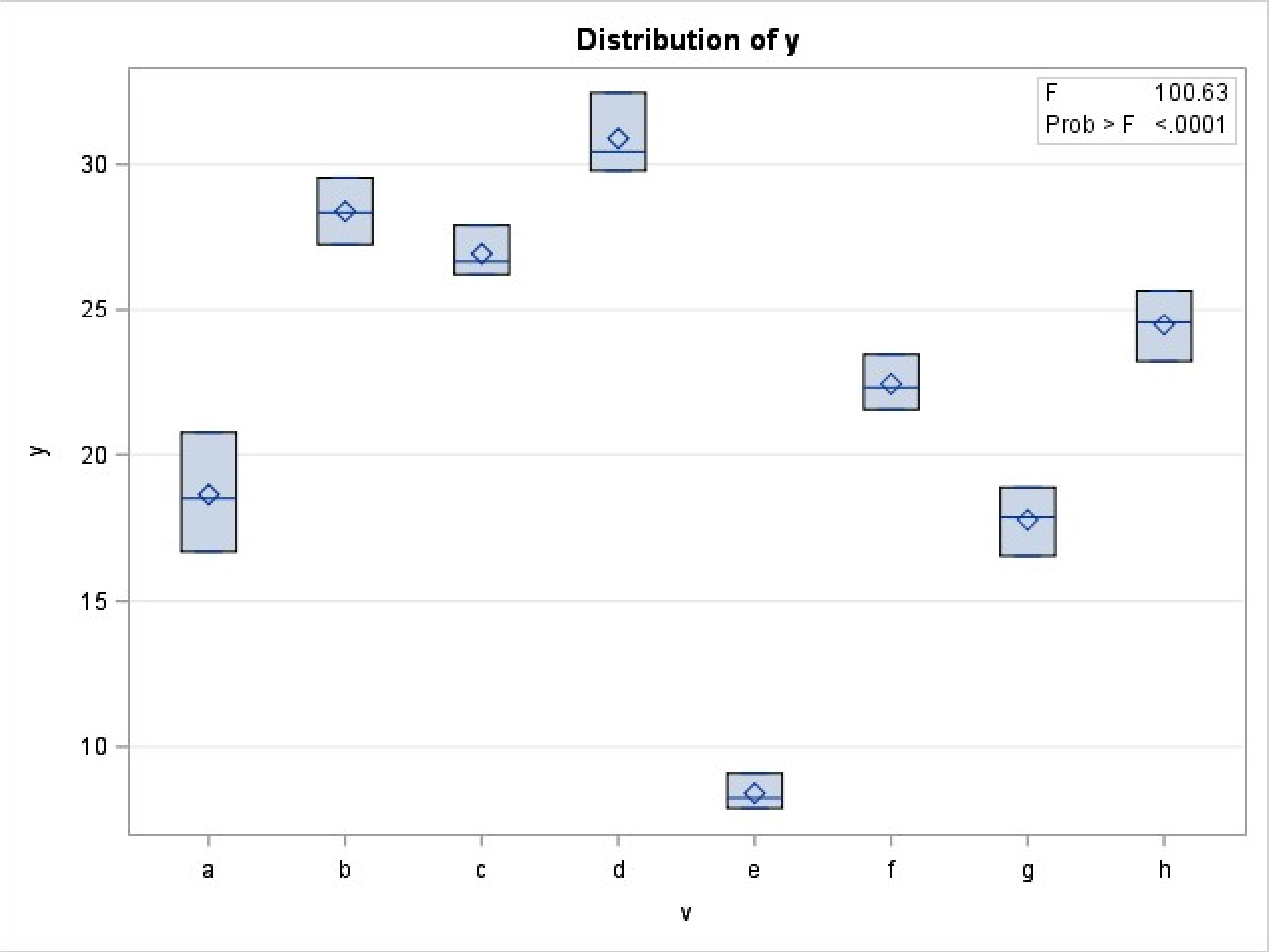

onion day

The ANOVA Procedure

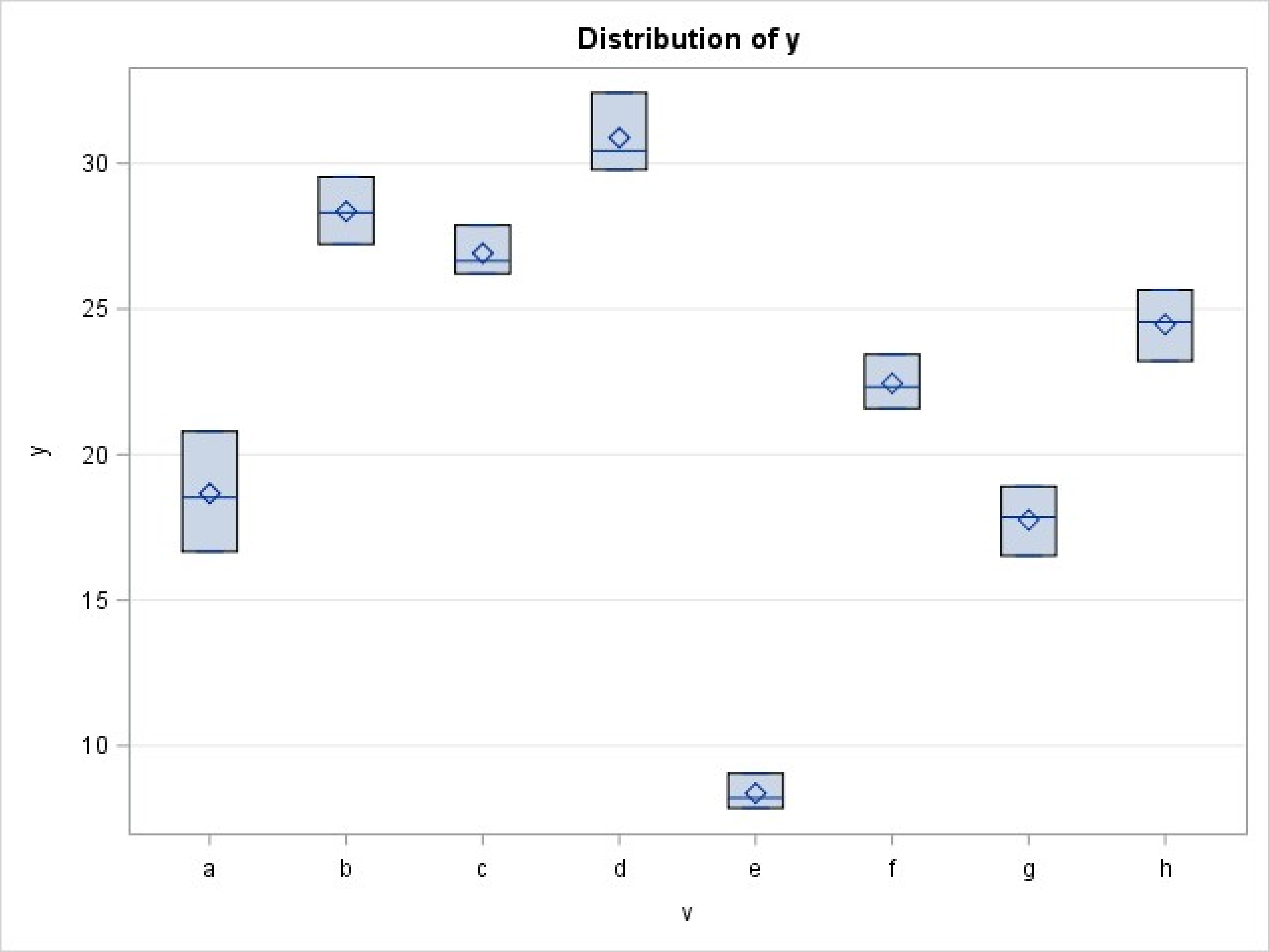

onion day

The ANOVA Procedure

Duncan's Multiple Range Test for y

Note: This test controls the Type I comparisonwise error rate, not the experimentwise error rate.

**Alpha**	0.05
**Error Degrees of Freedom**	16
**Error Mean Square**	1.549446

**Number of Means**	**2**	**3**	**4**	**5**	**6**	**7**	**8**
**Critical Range**	2.154	2.259	2.325	2.370	2.402	2.426	2.445

**Means with the same letter are not significantly different.**			
**Duncan Grouping**	**Mean**	**N**	**v**
A	30.883	6	d
B	28.363	6	b
B			
B	26.917	6	c
C	24.477	6	h
C			
C	22.447	6	f
D	18.673	6	a
D			
D	17.773	6	g
E	8.383	6	e

V describes the treatments.

a; Control b; Si c; GA3 d; Si+GA3 e; Heat f; Si+Heat g; GA3+Heat h; Si+GA3+Heat

Figure 2C

onion day

The ANOVA Procedure

Class Level Information		
Class	Levels	Values
v	8	a b c d e f g h

Number of Observations Read	24
Number of Observations Used	24

onion day

The ANOVA Procedure

Dependent Variable: y

Source	DF	Sum of Squares	Mean Square	F Value	Pr > F
Model	7	61762.45440	8823.20777	333.00	<.0001
Error	16	423.93460	26.49591		
Corrected Total	23	62186.38900			

R-Square	Coeff Var	Root MSE	y Mean
0.993183	2.888974	5.147418	178.1746

Source	DF	Anova SS	Mean Square	F Value	Pr > F
v	7	61762.45440	8823.20777	333.00	<.0001

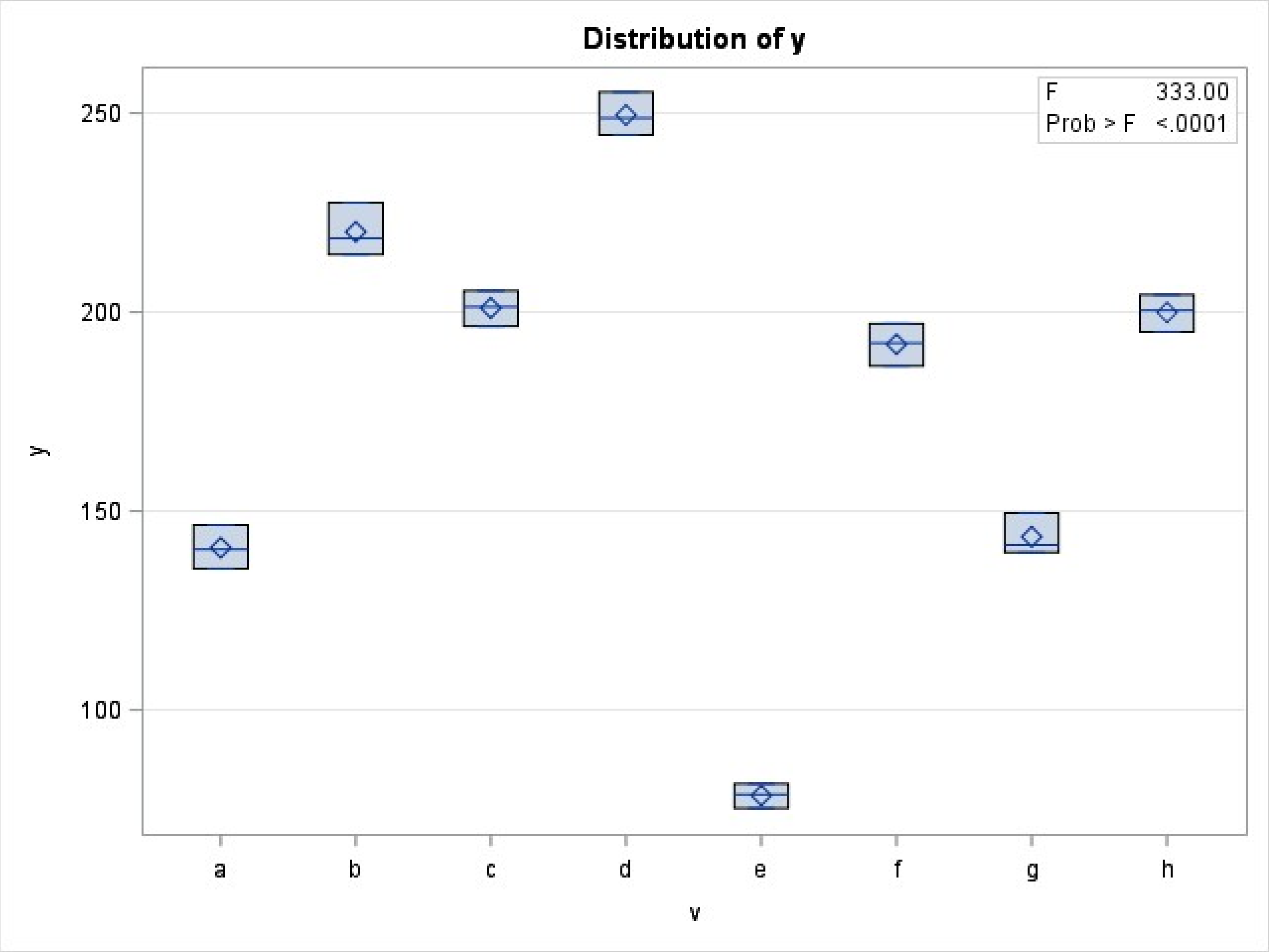

onion day

The ANOVA Procedure

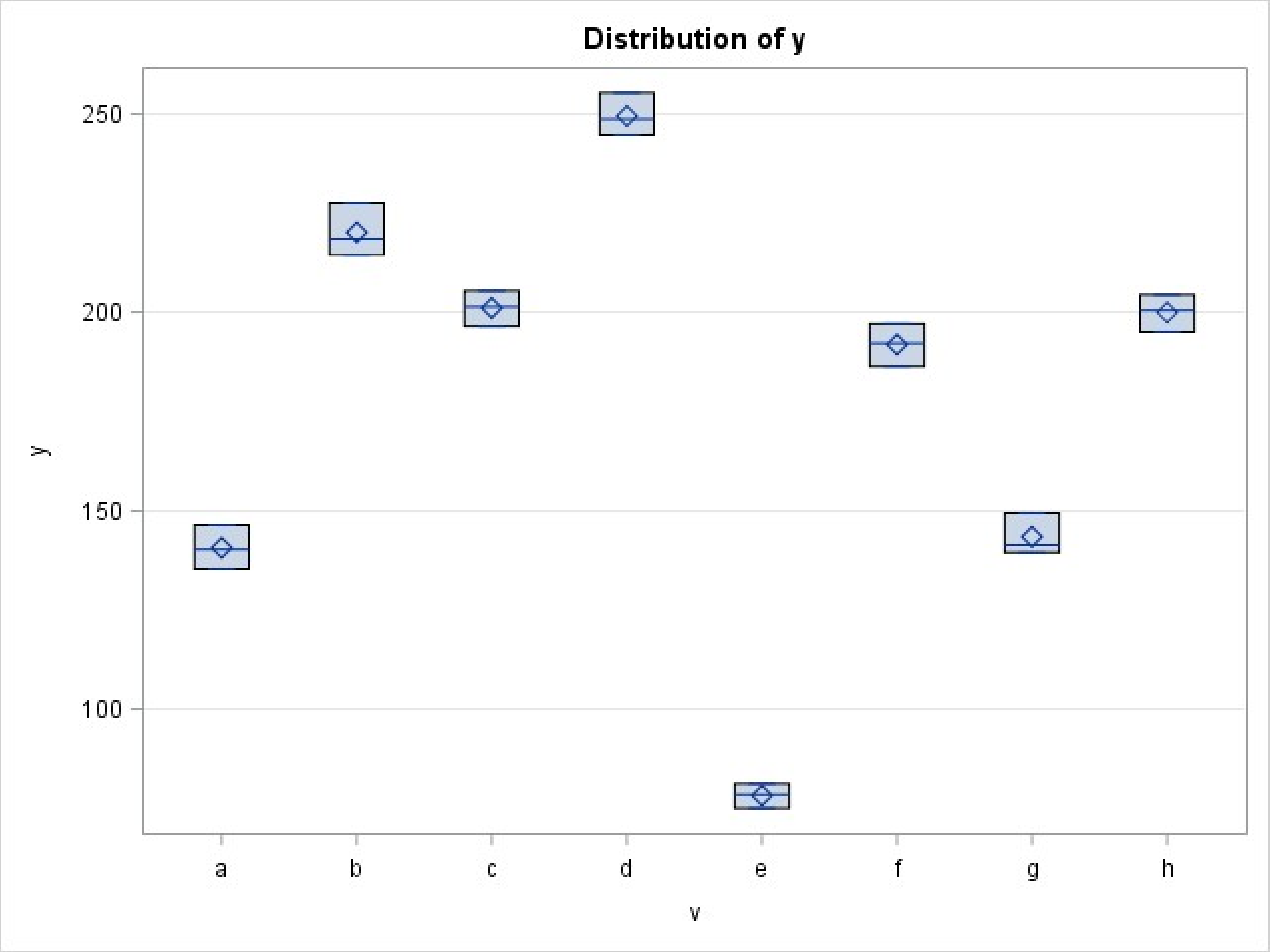

onion day

The ANOVA Procedure

Duncan's Multiple Range Test for y

Note: This test controls the Type I comparisonwise error rate, not the experimentwise error rate.

**Alpha**	0.05
**Error Degrees of Freedom**	16
**Error Mean Square**	26.49591

Number of Means	2	3	4	5	6	7	8
**Critical Range**	8.91	9.34	9.61	9.80	9.93	10.03	10.11

Means with the same letter are not significantly different.			
**Duncan Grouping**	**Mean**	**N**	**v**
A	249.540	6	d
B	220.170	6	b
C	201.030	6	c
C			
C	199.930	6	h
C			
C	191.917	6	f
D	143.573	6	g
D			
D	140.837	6	a
E	78.400	6	e

V describes the treatments.

a; Control b; Si c; GA3 d; Si+GA3 e; Heat f; Si+Heat g; GA3+Heat h; Si+GA3+Heat

Figure 2D

onion day

The ANOVA Procedure

Class Level Information		
Class	Levels	Values
v	8	a b c d e f g h

Number of Observations Read	24
Number of Observations Used	24

onion day

The ANOVA Procedure

Dependent Variable: y

Source	DF	Sum of Squares	Mean Square	F Value	Pr > F
Model	7	5429.403029	775.629004	119.38	<.0001
Error	16	103.951067	6.496942		
Corrected Total	23	5533.354096			

R-Square	Coeff Var	Root MSE	y Mean
0.981214	3.020736	2.548910	84.38042

Source	DF	Anova SS	Mean Square	F Value	Pr > F
v	7	5429.403029	775.629004	119.38	<.0001

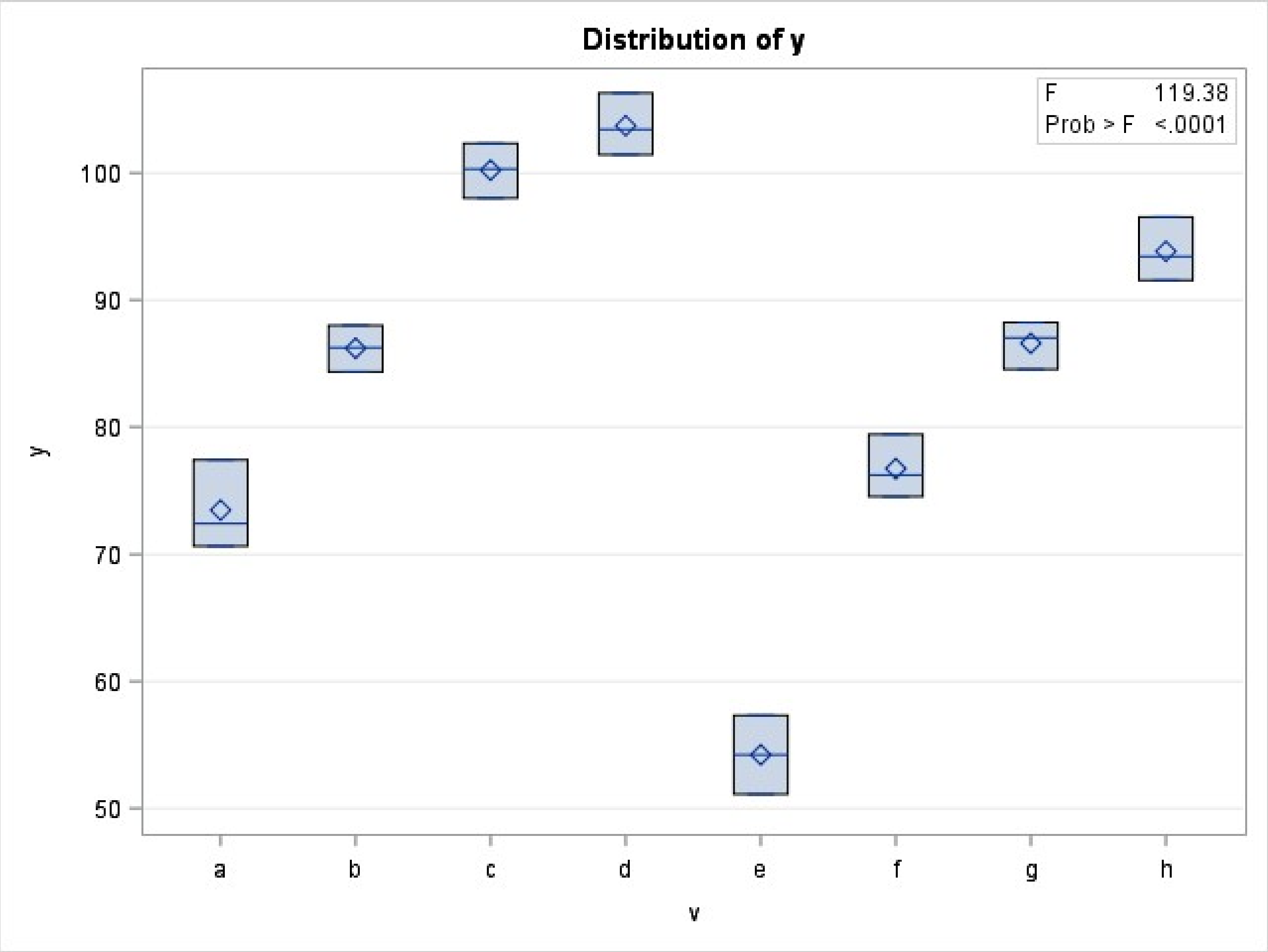

onion day

The ANOVA Procedure

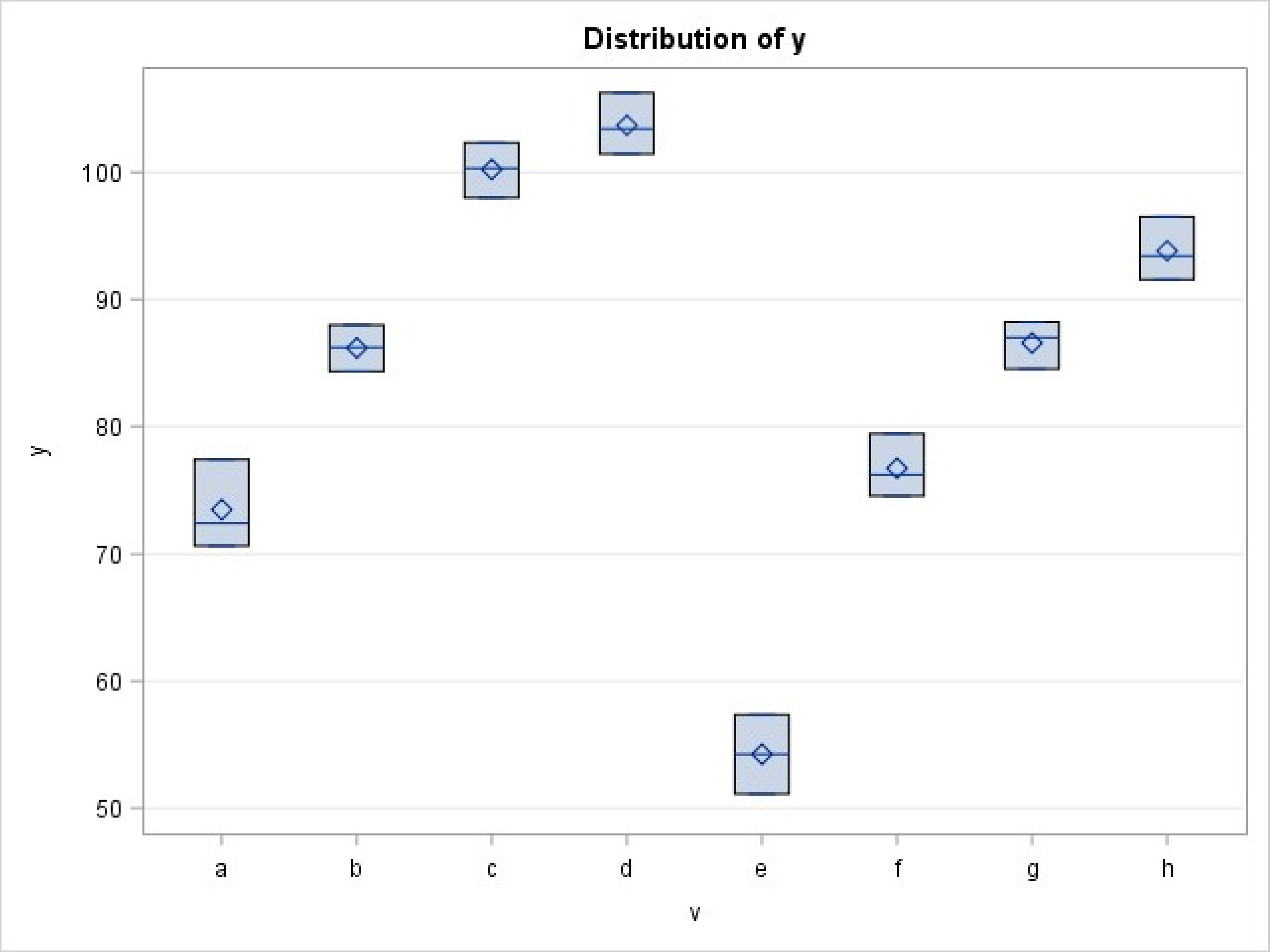

onion day

The ANOVA Procedure

Duncan's Multiple Range Test for y

Note: This test controls the Type I comparisonwise error rate, not the experimentwise error rate.

**Alpha**	0.05
**Error Degrees of Freedom**	16
**Error Mean Square**	6.496942

**Number of Means**	**2**	**3**	**4**	**5**	**6**	**7**	**8**
**Critical Range**	4.412	4.626	4.761	4.853	4.919	4.969	5.006

**Means with the same letter are not significantly different.**			
**Duncan Grouping**	**Mean**	**N**	**v**
A	103.730	6	d
A			
A	100.227	6	c
B	93.840	6	h
C	86.597	6	g
C			
C	86.200	6	b
D	76.733	6	f
D			
D	73.497	6	a
E	54.220	6	e

V describes the treatments.

a; Control b; Si c; GA3 d; Si+GA3 e; Heat f; Si+Heat g; GA3+Heat h; Si+GA3+Heat

onion day

The ANOVA Procedure

Class Level Information		
Class	Levels	Values
v	8	a b c d e f g h

Number of Observations Read	24
Number of Observations Used	24

Figure 3A

onion day

The ANOVA Procedure

Dependent Variable: y

Source	DF	Sum of Squares	Mean Square	F Value	Pr > F
Model	7	10936.27856	1562.32551	166.51	<.0001
Error	16	150.12280	9.38267		
Corrected Total	23	11086.40136			

R-Square	Coeff Var	Root MSE	y Mean
0.986459	5.721572	3.063115	53.53625

Source	DF	Anova SS	Mean Square	F Value	Pr > F
v	7	10936.27856	1562.32551	166.51	<.0001

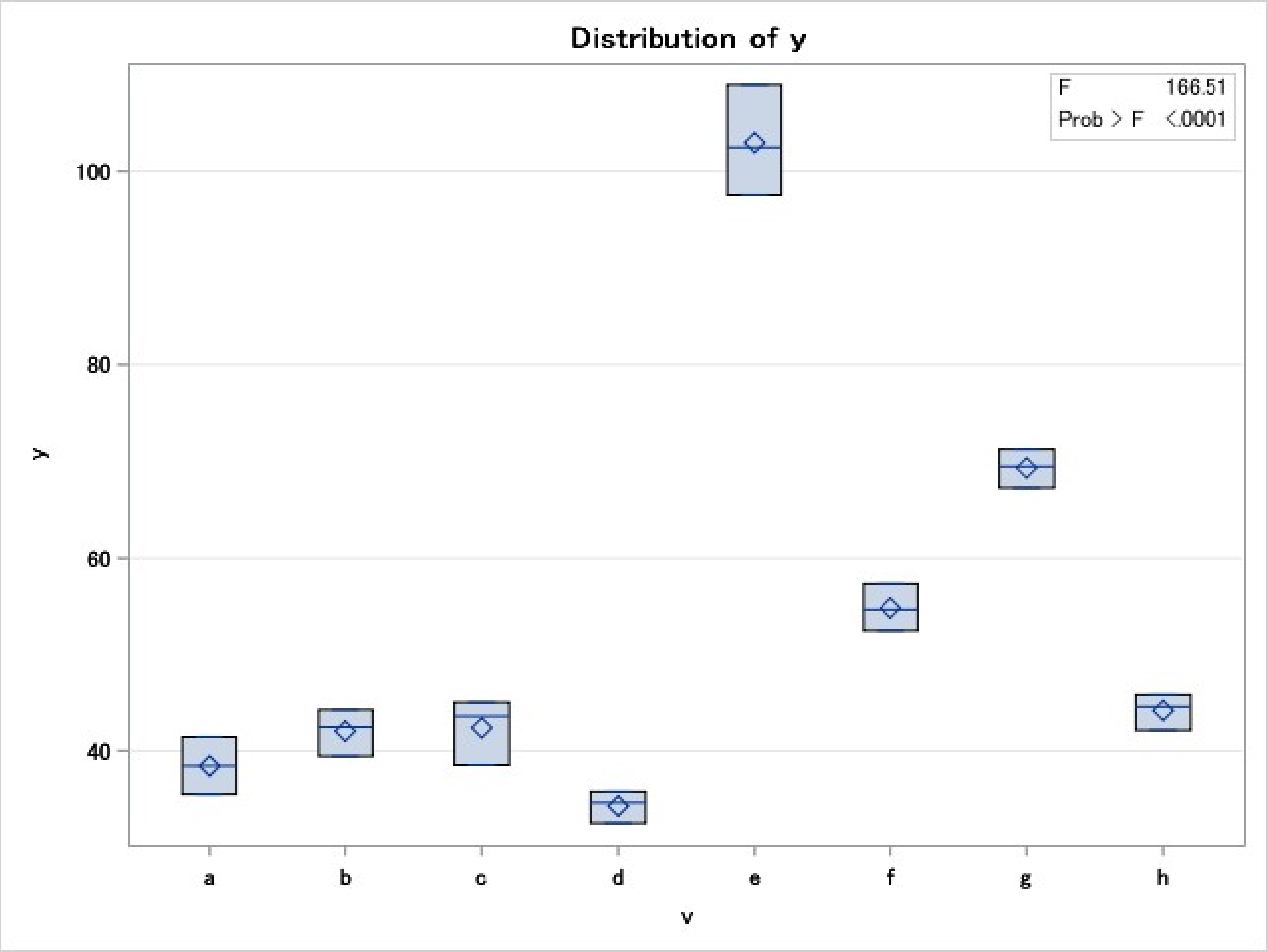

onion day

The ANOVA Procedure

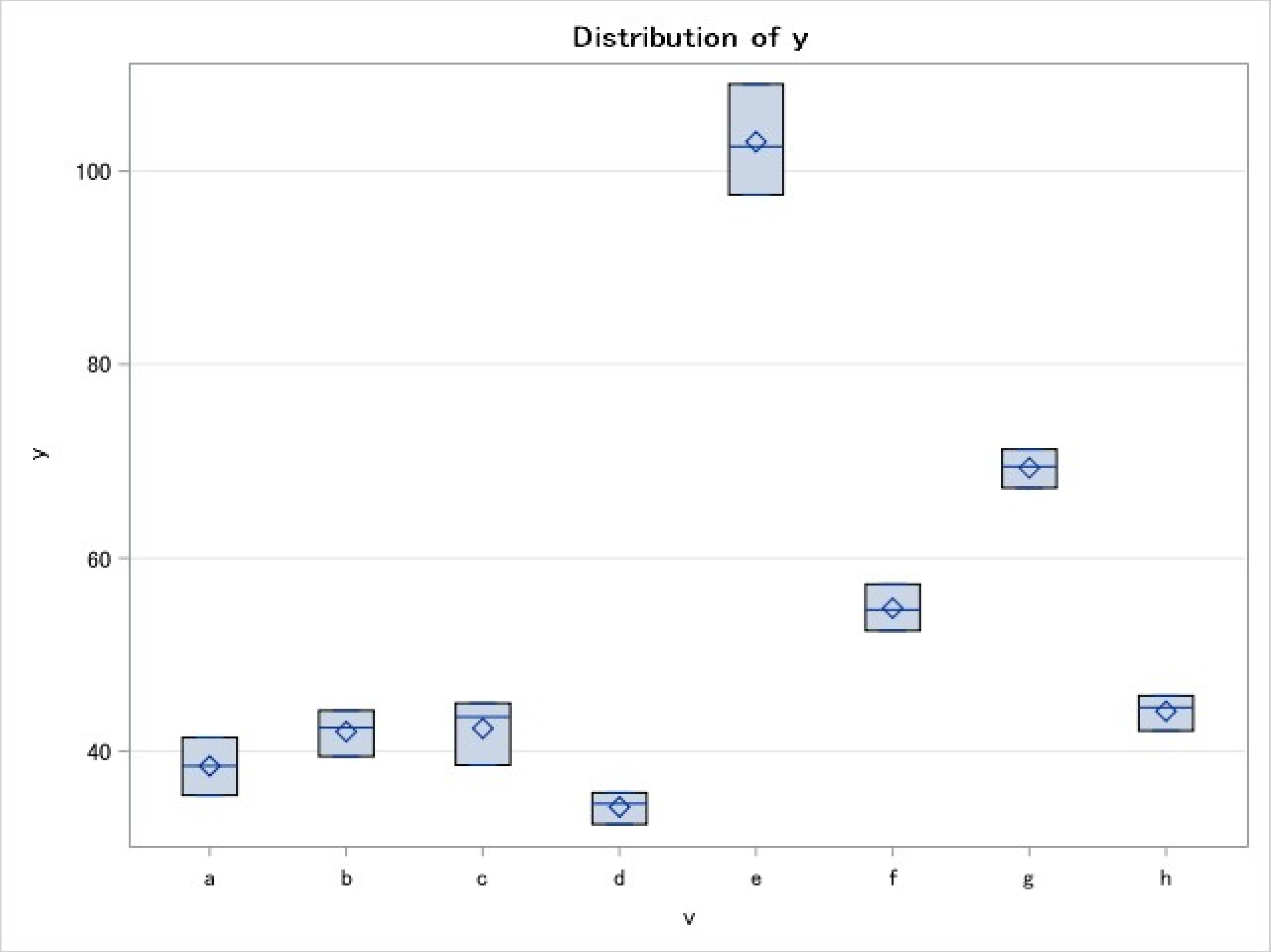

onion day

The ANOVA Procedure

Duncan's Multiple Range Test for y

Note: This test controls the Type I comparisonwise error rate, not the experimentwise error rate.

Alpha	0.05
Error Degrees of Freedom	16
Error Mean Square	9.382675

Number of Means	2	3	4	5	6	7	8
Critical Range	5.302	5.560	5.721	5.831	5.911	5.971	6.016

Means with the same letter are not significantly different.

Duncan Grouping		Mean	N	v
	A	103.027	4	e
	B	69.290	4	g
	C	54.780	4	f
	D	44.140	4	h
	D			
	D	42.360	4	c
	D			
	D	42.027	4	b
	D			
E	D	38.437	4	a
E				
E		34.230	4	d

V describes the treatments.

a; Control b; Si c; GA3 d; Si+GA3 e; Heat f; Si+Heat g; GA3+Heat h; Si+GA3+Heat

Figure 3B

onion day

The ANOVA Procedure

Class Level Information		
Class	Levels	Values
v	8	a b c d e f g h

Number of Observations Read	24
Number of Observations Used	24

onion day

The ANOVA Procedure

Dependent Variable: y

Source	DF	Sum of Squares	Mean Square	F Value	Pr > F
Model	7	1005.654200	143.664886	164.29	<.0001
Error	16	13.991600	0.874475		
Corrected Total	23	1019.645800			

R-Square	Coeff Var	Root MSE	y Mean
0.986278	8.642640	0.935134	10.82000

Source	DF	Anova SS	Mean Square	F Value	Pr > F
v	7	1005.654200	143.664886	164.29	<.0001

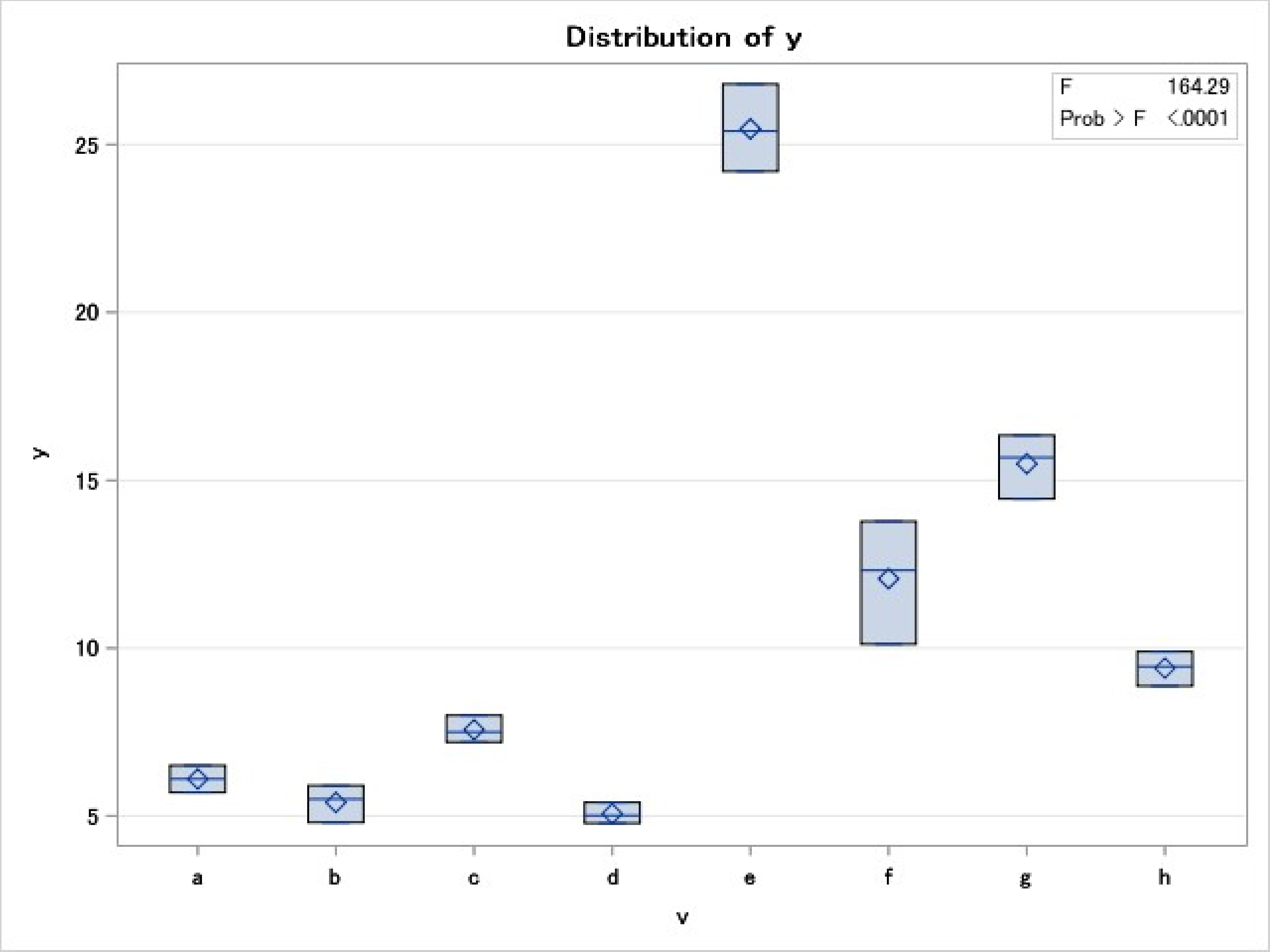

onion day

The ANOVA Procedure

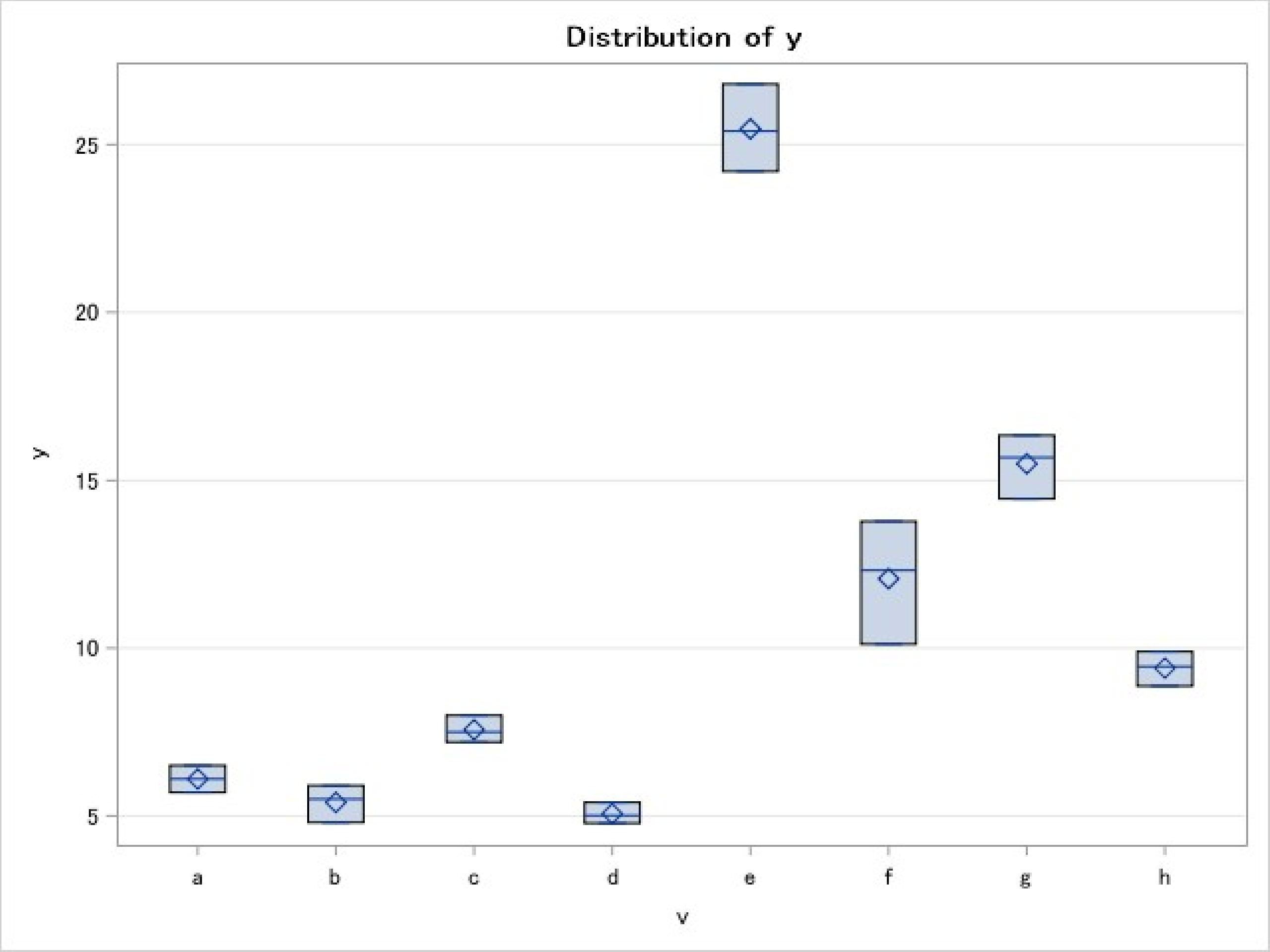

onion day

The ANOVA Procedure

Duncan's Multiple Range Test for y

Note: This test controls the Type I comparisonwise error rate, not the experimentwise error rate.

Alpha	0.05
Error Degrees of Freedom	16
Error Mean Square	0.874475

Number of Means	2	3	4	5	6	7	8
Critical Range	1.619	1.697	1.747	1.780	1.805	1.823	1.837

Means with the same letter are not significantly different.

Duncan Grouping		Mean	N	v
	A	25.4667	4	e
	B	15.4833	4	g
	C	12.0733	4	f
	D	9.4067	4	h
	E	7.5667	4	c
	E			
F	E	6.1000	4	a
F				
F		5.4000	4	b
F				
F		5.0633	4	d

V describes the treatments.

a; Control b; Si c; GA3 d; Si+GA3 e; Heat f; Si+Heat g; GA3+Heat h; Si+GA3+Heat

Figure 3C

onion day

The ANOVA Procedure

Class Level Information		
Class	Levels	Values
v	8	a b c d e f g h

Number of Observations Read	24
Number of Observations Used	24

onion day

The ANOVA Procedure

Dependent Variable: y

Source	DF	Sum of Squares	Mean Square	F Value	Pr > F
Model	7	11254.83210	1607.83316	464.52	<.0001
Error	16	55.37987	3.46124		
Corrected Total	23	11310.21196			

R-Square	Coeff Var	Root MSE	y Mean
0.995104	9.393203	1.860441	19.80625

Source	DF	Anova SS	Mean Square	F Value	Pr > F
v	7	11254.83210	1607.83316	464.52	<.0001

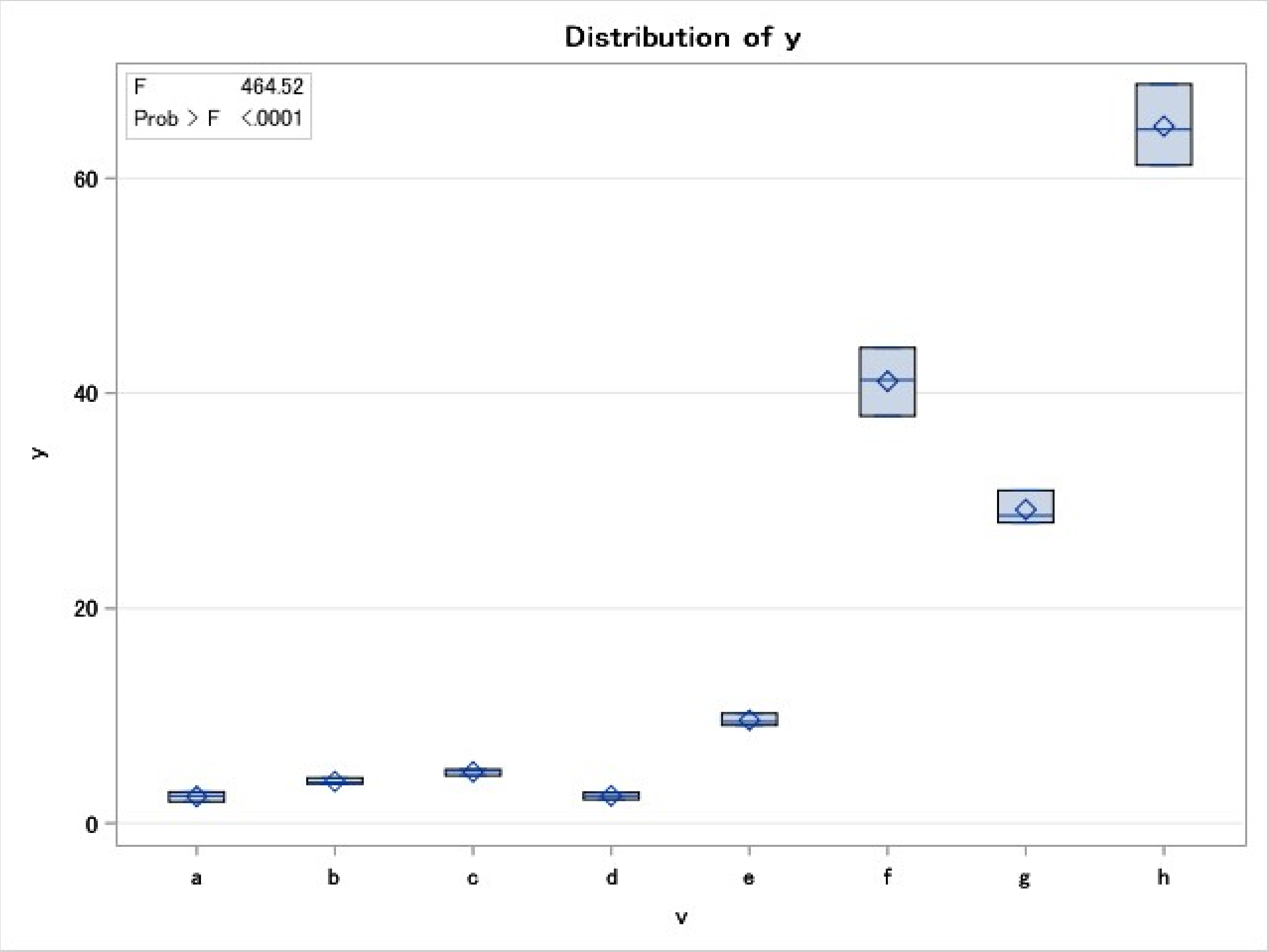

onion day

The ANOVA Procedure

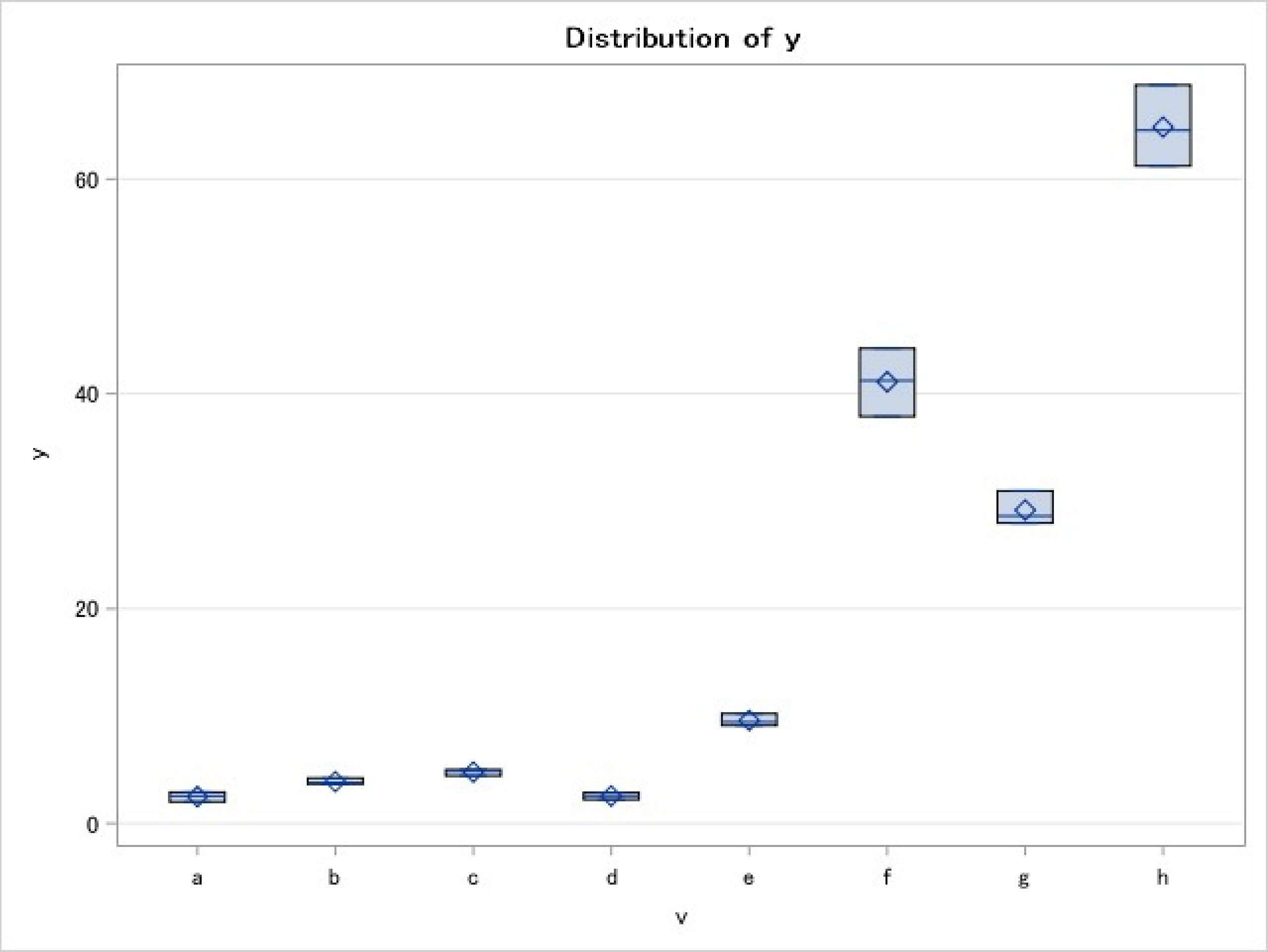

onion day

The ANOVA Procedure

Duncan's Multiple Range Test for y

Note: This test controls the Type I comparisonwise error rate, not the experimentwise error rate.

Alpha	0.05
Error Degrees of Freedom	16
Error Mean Square	3.461242

Number of Means	2	3	4	5	6	7	8
Critical Range	3.220	3.377	3.475	3.542	3.590	3.627	3.654

Means with the same letter are not significantly different.			
Duncan Grouping	Mean	N	v
A	64.843	4	h
B	41.107	4	f
C	29.203	4	g
D	9.593	4	e
E	4.773	4	c
E			
E	3.893	4	b
E			
E	2.547	4	d
E			
E	2.490	4	a

V describes the treatments.

a; Control b; Si c; GA3 d; Si+GA3 e; Heat f; Si+Heat g; GA3+Heat h; Si+GA3+Heat

Figure 3D

onion day

The ANOVA Procedure

Class Level Information		
Class	Levels	Values
v	8	a b c d e f g h

Number of Observations Read	24
Number of Observations Used	24

onion day

The ANOVA Procedure

Dependent Variable: y

Source	DF	Sum of Squares	Mean Square	F Value	Pr > F
Model	7	22672.02218	3238.86031	438.29	<.0001
Error	16	118.23540	7.38971		
Corrected Total	23	22790.25758			

R-Square	Coeff Var	Root MSE	y Mean
0.994812	5.638767	2.718403	48.20917

Source	DF	Anova SS	Mean Square	F Value	Pr > F
v	7	22672.02218	3238.86031	438.29	<.0001

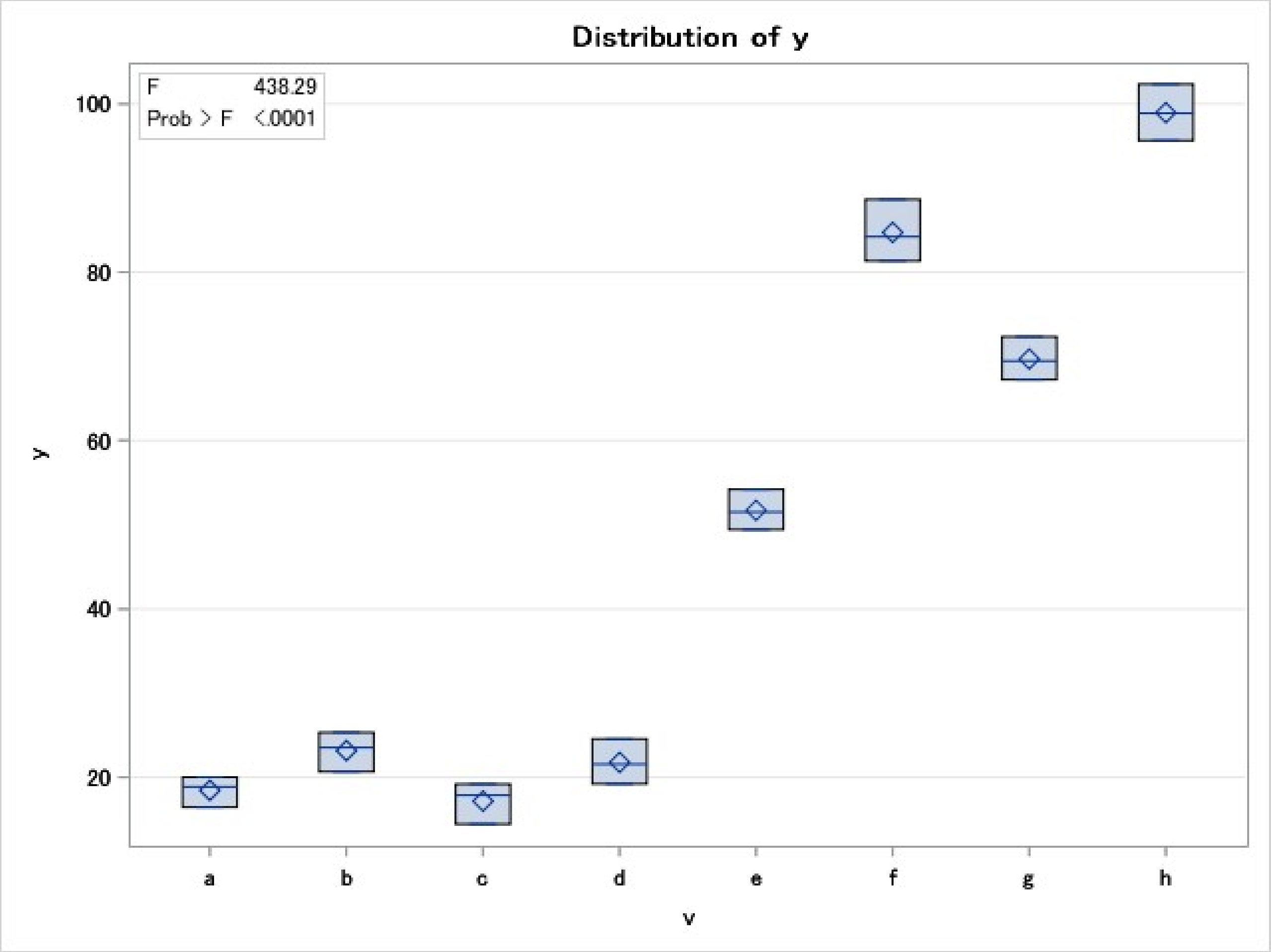

onion day

The ANOVA Procedure

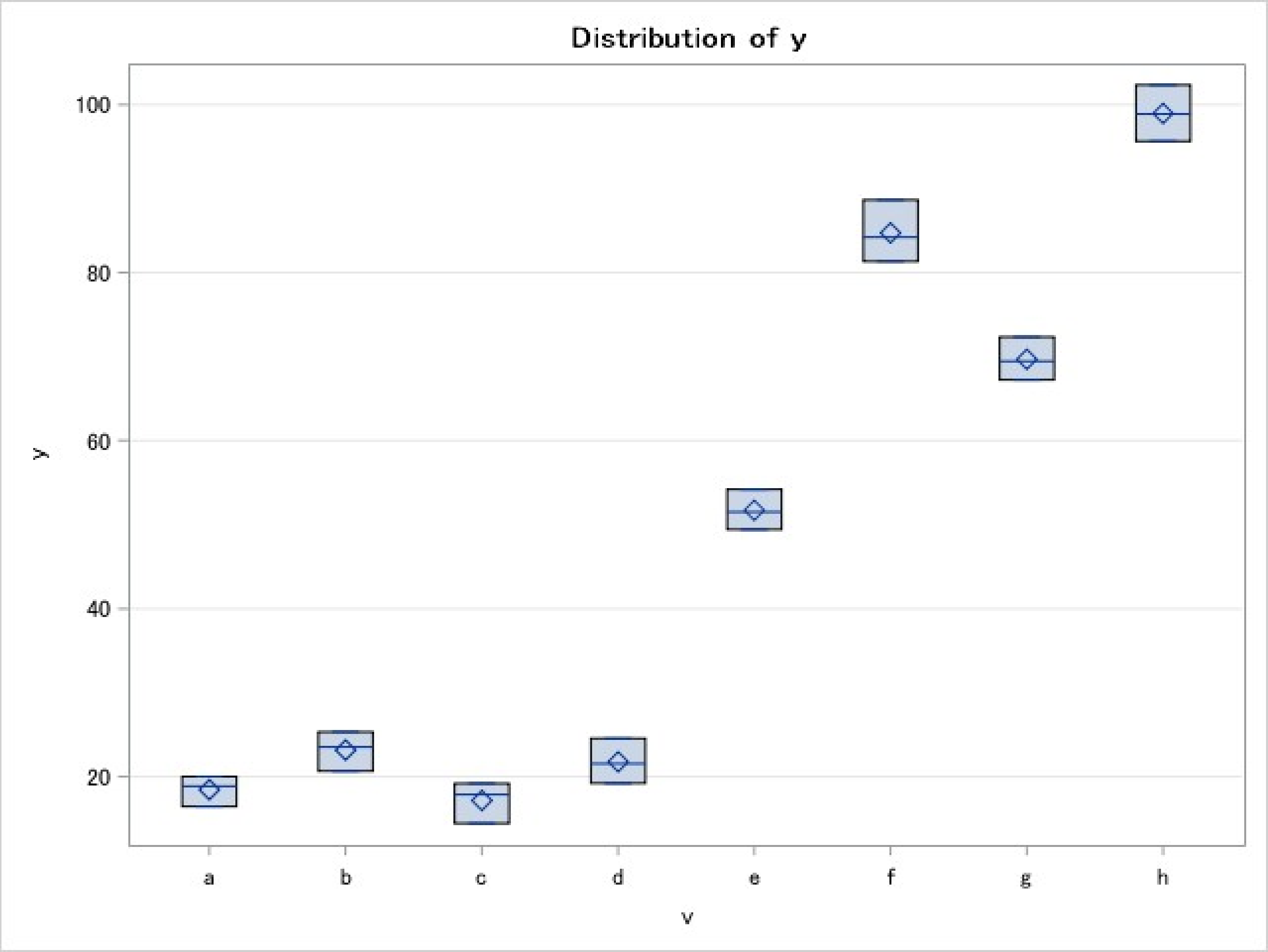

onion day

The ANOVA Procedure

Duncan's Multiple Range Test for y

Note: This test controls the Type I comparisonwise error rate, not the experimentwise error rate.

Alpha	0.05
Error Degrees of Freedom	16
Error Mean Square	7.389713

Number of Means	2	3	4	5	6	7	8
Critical Range	4.705	4.934	5.077	5.175	5.246	5.299	5.339

Means with the same letter are not significantly different.

Duncan Grouping		Mean	N	v
	A	98.963	4	h
	B	84.737	4	f
	C	69.663	4	g
	D	51.737	4	e
	E	23.180	4	b
	E			
F	E	21.777	4	d
F	E			
F	E	18.447	4	a
F				
F		17.170	4	c

V describes the treatments.

a; Control b; Si c; GA3 d; Si+GA3 e; Heat f; Si+Heat g; GA3+Heat h; Si+GA3+Heat

Figure 3E

onion day

The ANOVA Procedure

Class Level Information		
Class	Levels	Values
v	8	a b c d e f g h

Number of Observations Read	24
Number of Observations Used	24

onion day

The ANOVA Procedure

Dependent Variable: y

Source	DF	Sum of Squares	Mean Square	F Value	Pr > F
Model	7	18744.57593	2677.79656	289.45	<.0001
Error	16	148.02087	9.25130		
Corrected Total	23	18892.59680			

R-Square	Coeff Var	Root MSE	y Mean
0.992165	4.659664	3.041596	65.27500

Source	DF	Anova SS	Mean Square	F Value	Pr > F
v	7	18744.57593	2677.79656	289.45	<.0001

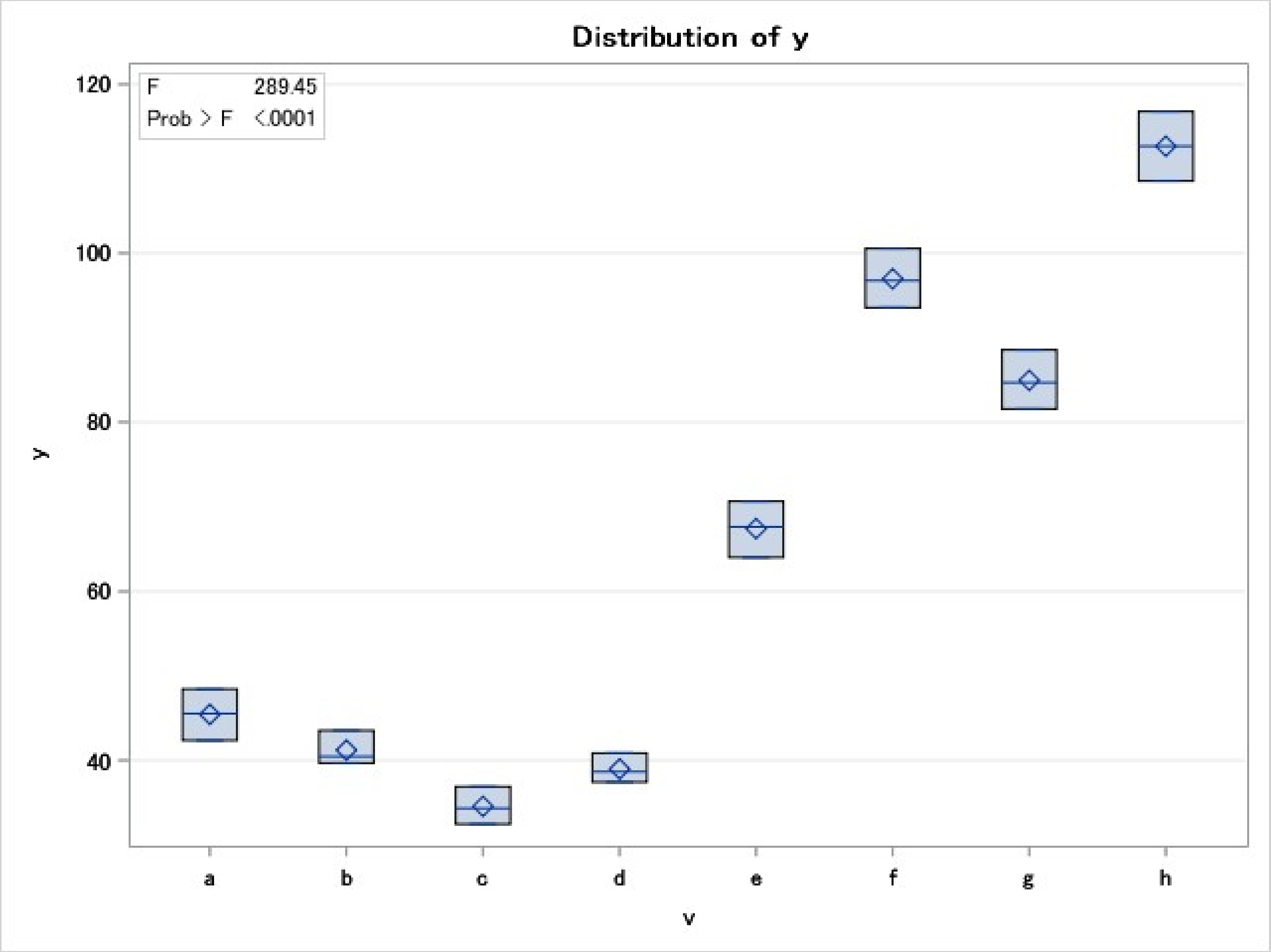

onion day

The ANOVA Procedure

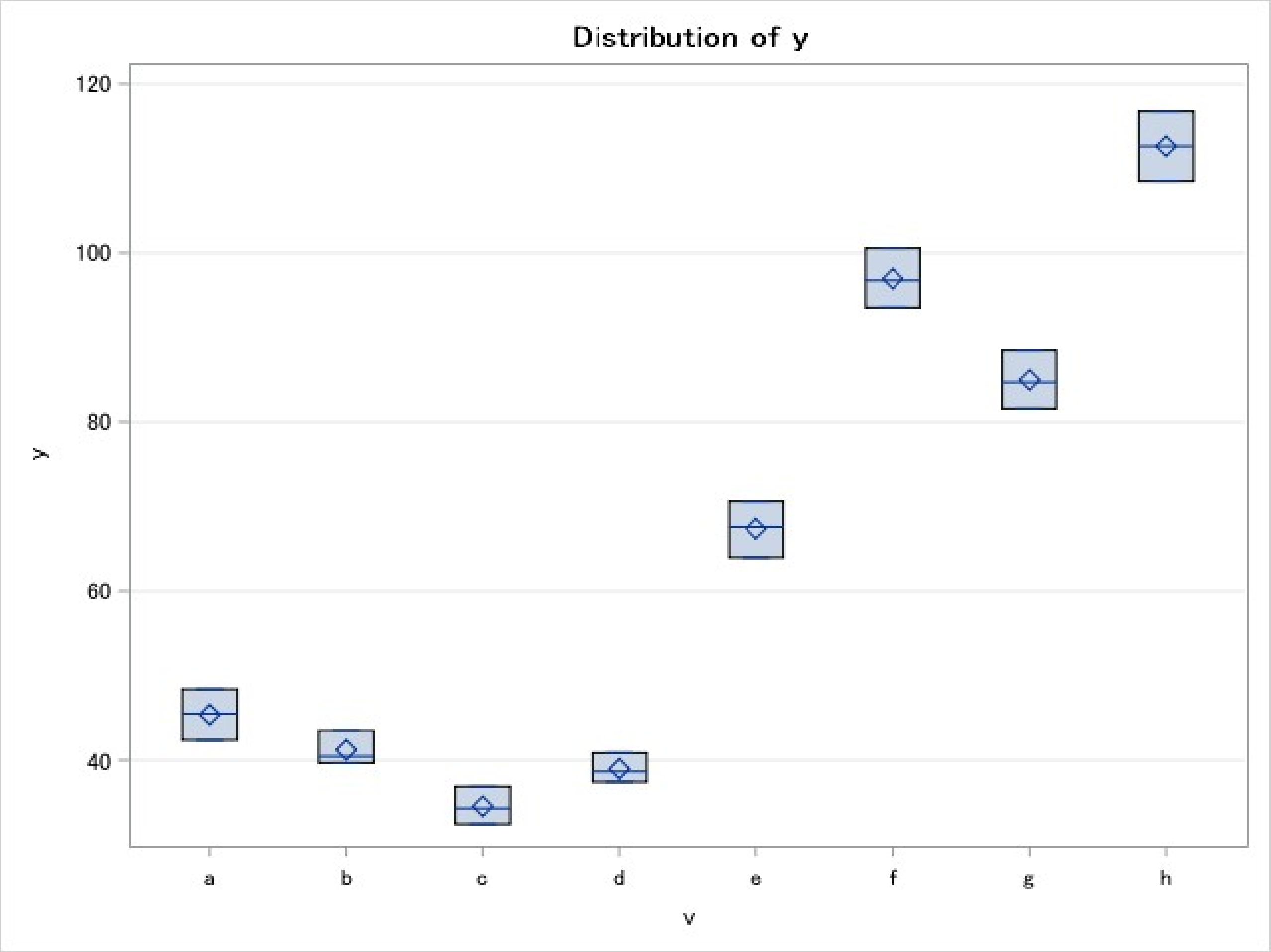

onion day

The ANOVA Procedure

Duncan's Multiple Range Test for y

Note: This test controls the Type I comparisonwise error rate, not the experimentwise error rate.

Alpha	0.05
Error Degrees of Freedom	16
Error Mean Square	9.251304

Number of Means	2	3	4	5	6	7	8
Critical Range	5.264	5.521	5.681	5.791	5.870	5.929	5.974

Means with the same letter are not significantly different.

Duncan Grouping		Mean	N	v
	A	112.670	4	h
	B	96.957	4	f
	C	84.930	4	g
	D	67.447	4	e
	E	45.440	4	a
	E			
F	E	41.220	4	b
F				
F	G	38.983	4	d
	G			
	G	34.553	4	c

V describes the treatments.

a; Control b; Si c; GA3 d; Si+GA3 e; Heat f; Si+Heat g; GA3+Heat h; Si+GA3+Heat

Figure 3F

onion day

The ANOVA Procedure

Class Level Information		
Class	Levels	Values
v	8	a b c d e f g h

Number of Observations Read	24
Number of Observations Used	24

onion day

The ANOVA Procedure

Dependent Variable: y

Source	DF	Sum of Squares	Mean Square	F Value	Pr > F
Model	7	413.1727167	59.0246738	161.34	<.0001
Error	16	5.8532667	0.3658292		
Corrected Total	23	419.0259833			

R-Square	Coeff Var	Root MSE	y Mean
0.986031	7.268961	0.604838	8.320833

Source	DF	Anova SS	Mean Square	F Value	Pr > F
v	7	413.1727167	59.0246738	161.34	<.0001

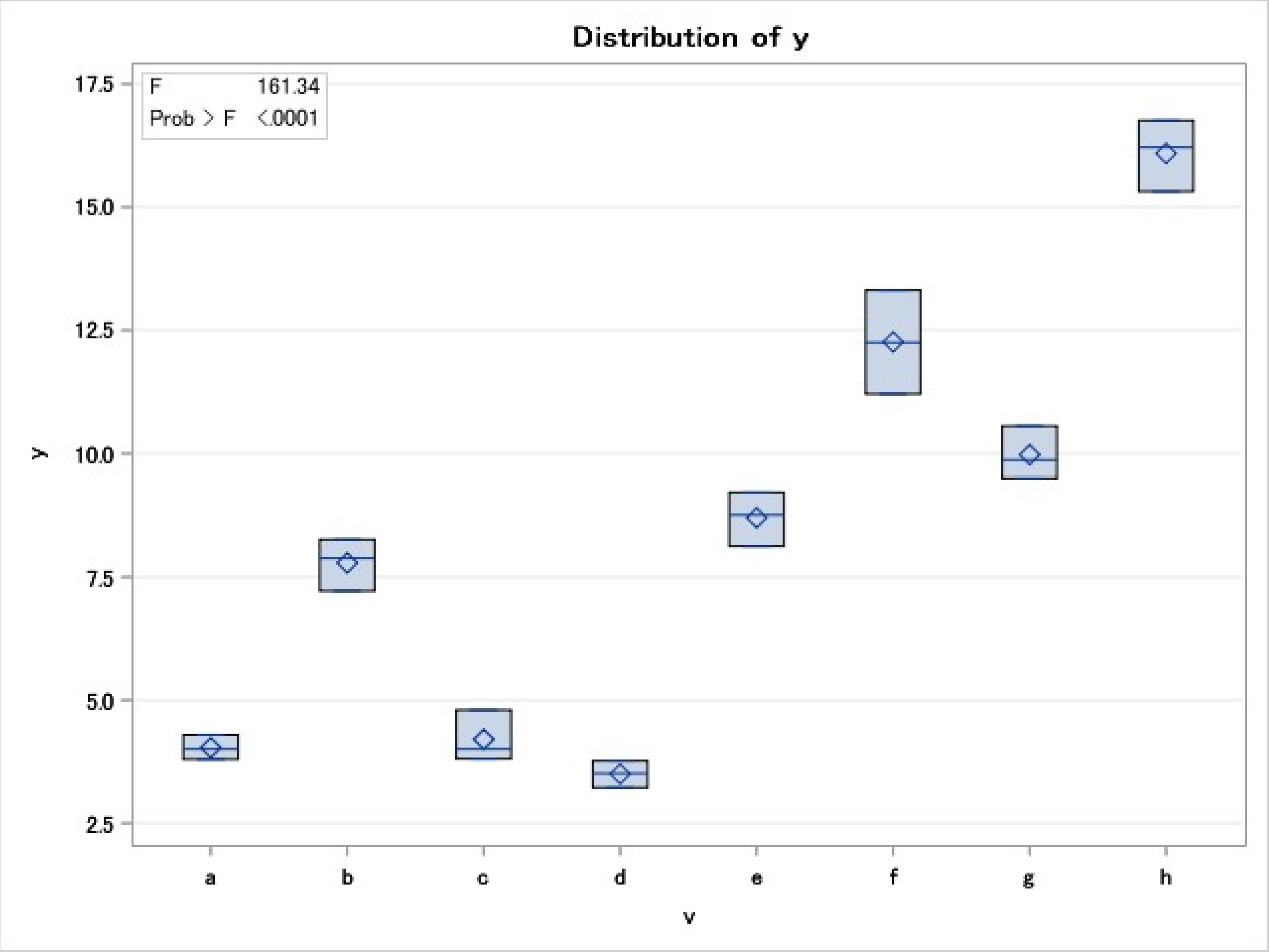

onion day

The ANOVA Procedure

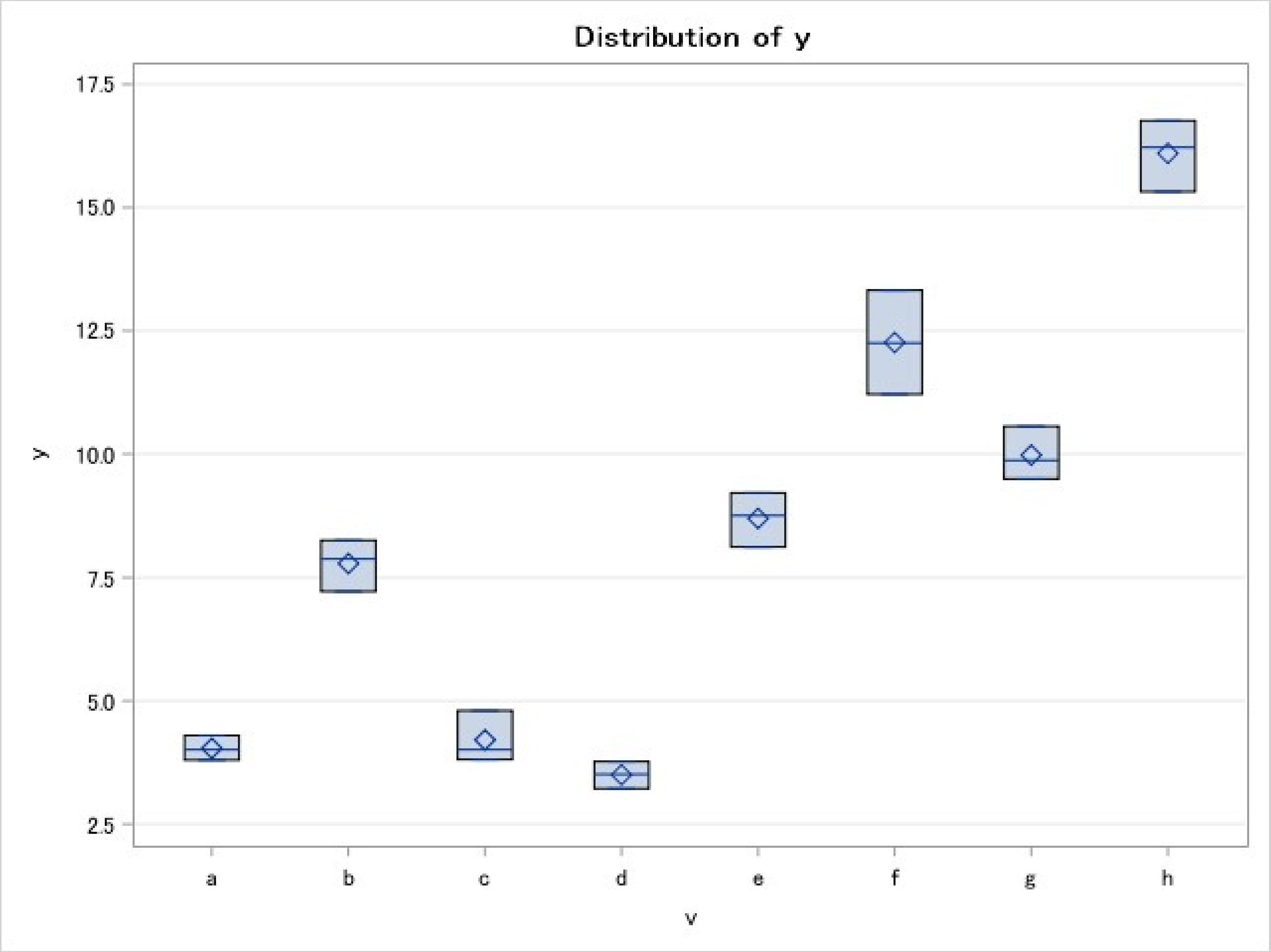

onion day

The ANOVA Procedure

Duncan's Multiple Range Test for y

Note: This test controls the Type I comparisonwise error rate, not the experimentwise error rate.

Alpha	0.05
Error Degrees of Freedom	16
Error Mean Square	0.365829

Number of Means	2	3	4	5	6	7	8
Critical Range	1.047	1.098	1.130	1.151	1.167	1.179	1.188

Means with the same letter are not significantly different.			
Duncan Grouping	Mean	N	v
A	16.1000	4	h
B	12.2600	4	f
C	9.9767	4	g
D	8.7000	4	e
D			
D	7.7867	4	b
E	4.2067	4	c
E			
E	4.0367	4	a
E			
E	3.5000	4	d

V describes the treatments.

a; Control b; Si c; GA3 d; Si+GA3 e; Heat f; Si+Heat g; GA3+Heat h; Si+GA3+Heat

onion day

The ANOVA Procedure

Class Level Information		
Class	Levels	Values
v	8	a b c d e f g h

Number of Observations Read	24
Number of Observations Used	24

Figure 4A

onion day

The ANOVA Procedure

Dependent Variable: y

Source	DF	Sum of Squares	Mean Square	F Value	Pr > F
Model	7	37650.68403	5378.66915	214.51	<.0001
Error	16	401.18670	25.07417		
Corrected Total	23	38051.87073			

R-Square	Coeff Var	Root MSE	y Mean
0.989457	5.904166	5.007411	84.81150

Source	DF	Anova SS	Mean Square	F Value	Pr > F
v	7	37650.68403	5378.66915	214.51	<.0001

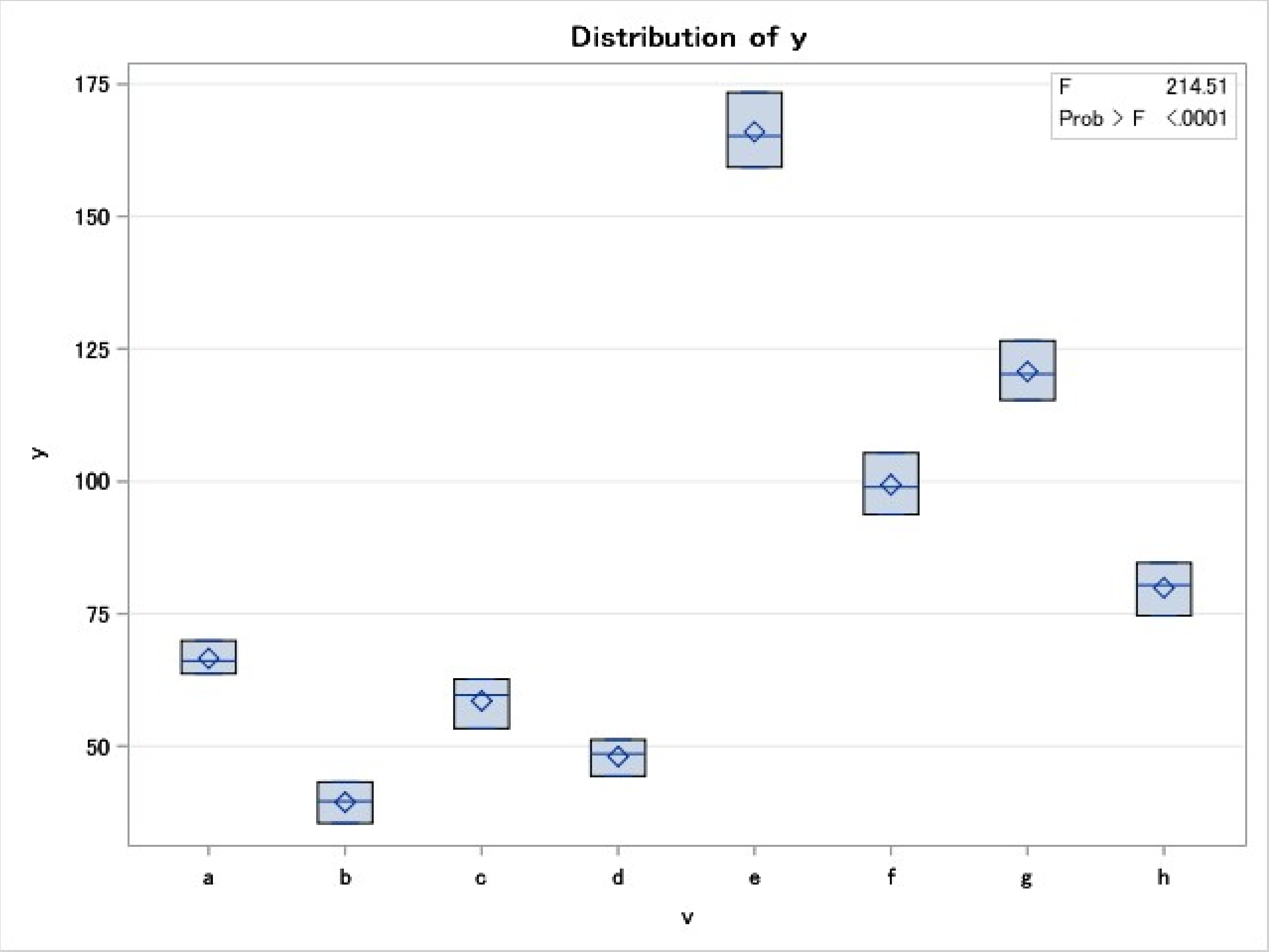

onion day

The ANOVA Procedure

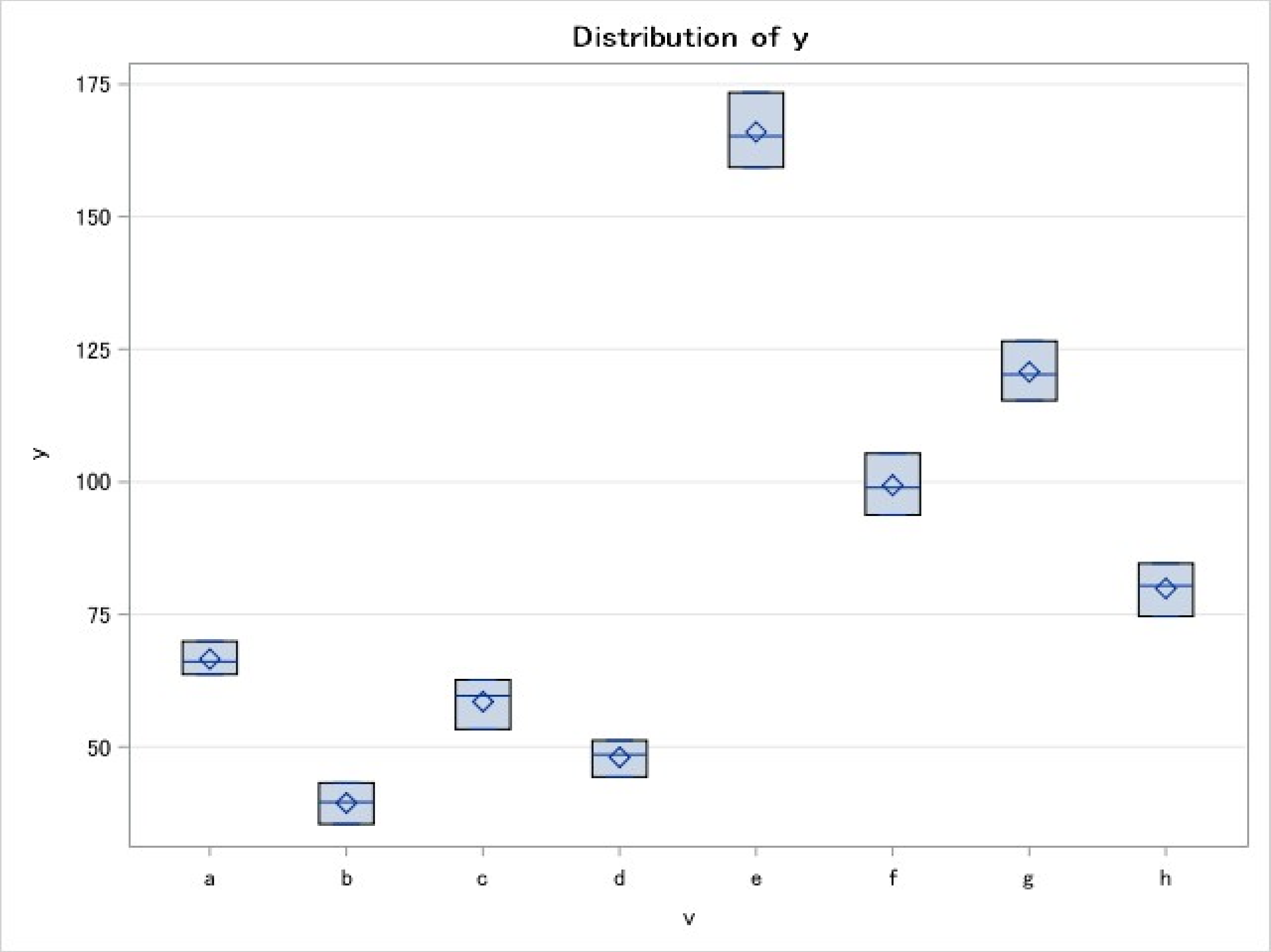

onion day

The ANOVA Procedure

Duncan's Multiple Range Test for y

Note: This test controls the Type I comparisonwise error rate, not the experimentwise error rate.

Alpha	0.05
Error Degrees of Freedom	16
Error Mean Square	25.07417

Number of Means	2	3	4	5	6	7	8
Critical Range	8.667	9.089	9.352	9.533	9.664	9.761	9.835

Means with the same letter are not significantly different.			
Duncan Grouping	Mean	N	v
A	165.990	4	e
B	120.687	4	g
C	99.359	4	f
D	79.907	4	h
E	66.570	4	a
E			
E	58.548	4	c
F	48.027	4	d
F			
F	39.404	4	b

V describes the treatments.

a; Control b; Si c; GA3 d; Si+GA3 e; Heat f; Si+Heat g; GA3+Heat h; Si+GA3+Heat

Figure 4B

onion day

The ANOVA Procedure

Class Level Information		
Class	Levels	Values
v	8	a b c d e f g h

Number of Observations Read	24
Number of Observations Used	24

onion day

The ANOVA Procedure

Dependent Variable: y

Source	DF	Sum of Squares	Mean Square	F Value	Pr > F
Model	7	29.52029583	4.21718512	29.33	<.0001
Error	16	2.30020000	0.14376250		
Corrected Total	23	31.82049583			

R-Square	Coeff Var	Root MSE	y Mean
0.927713	11.31681	0.379160	3.350417

Source	DF	Anova SS	Mean Square	F Value	Pr > F
v	7	29.52029583	4.21718512	29.33	<.0001

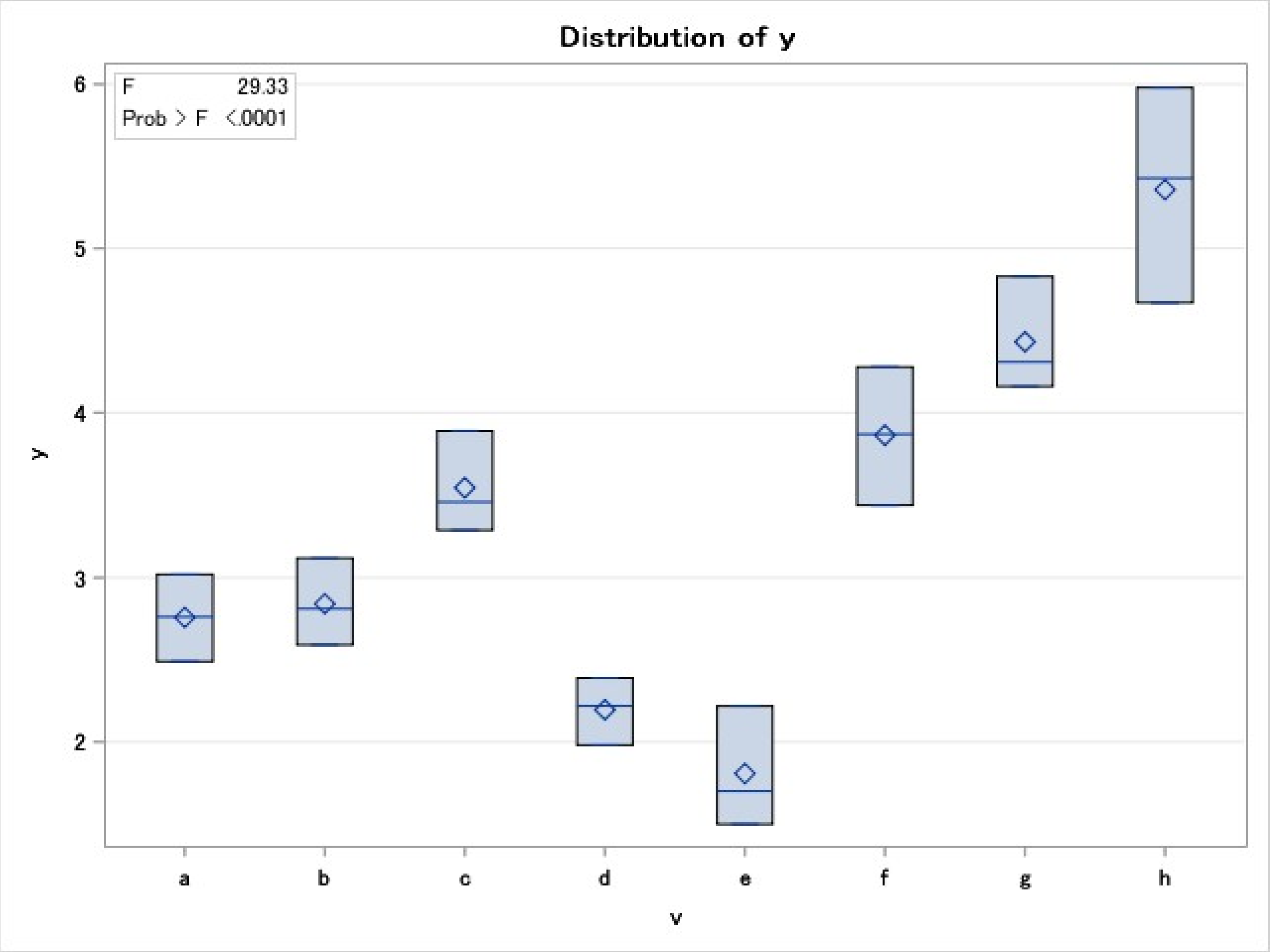

onion day

The ANOVA Procedure

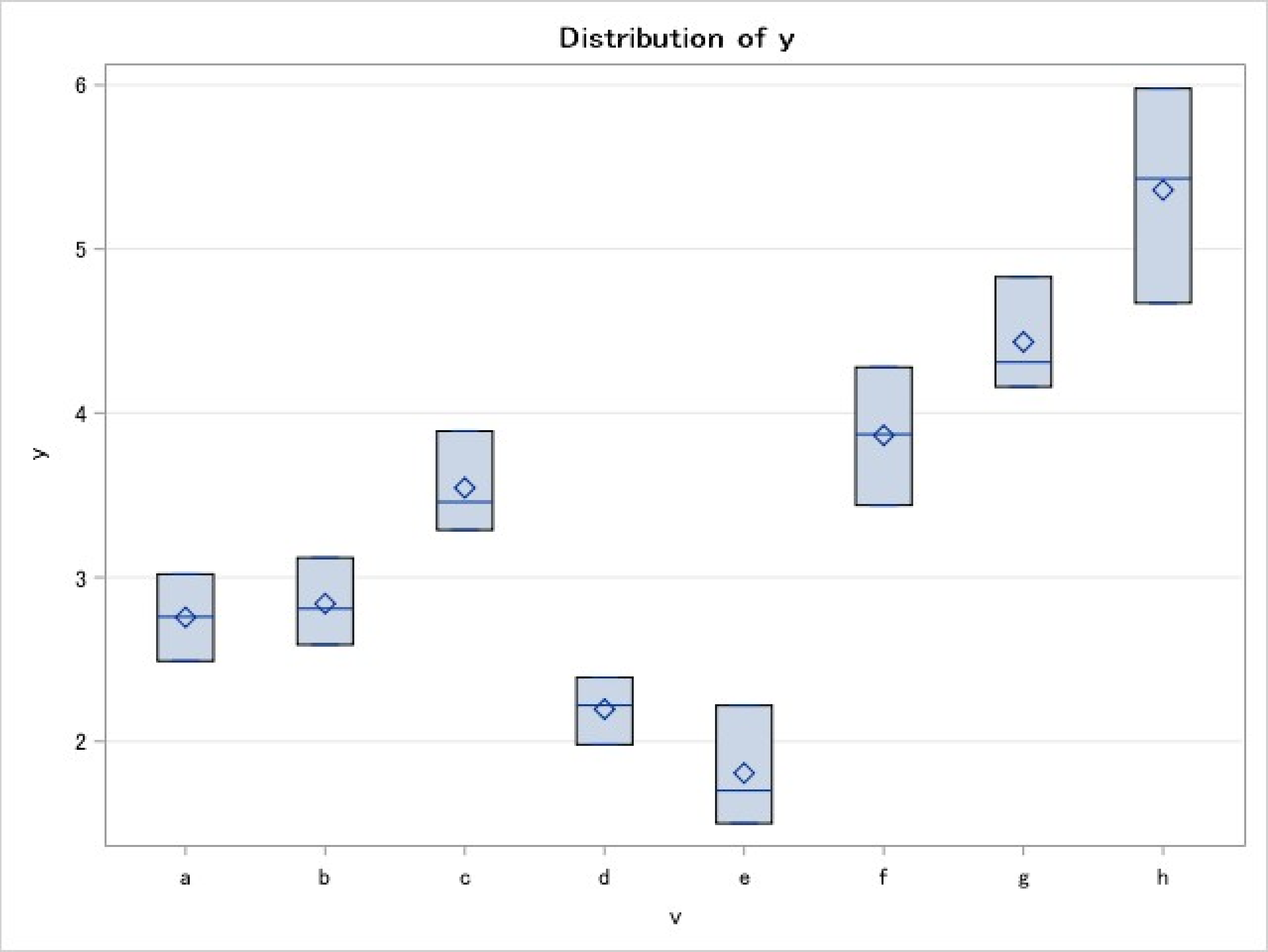

onion day

The ANOVA Procedure

Duncan's Multiple Range Test for y

Note: This test controls the Type I comparisonwise error rate, not the experimentwise error rate.

Alpha	0.05
Error Degrees of Freedom	16
Error Mean Square	0.143762

Number of Means	2	3	4	5	6	7	8
Critical Range	.6563	.6882	.7082	.7218	.7317	.7391	.7447

Means with the same letter are not significantly different.

Duncan Grouping		Mean	N	v
	A	5.3600	4	h
	B	4.4333	4	g
	B			
C	B	3.8633	4	f
C				
C		3.5467	4	c
	D	2.8400	4	b
	D			
	D	2.7567	4	a
	D			
E	D	2.1967	4	d
E				
E		1.8067	4	e

V describes the treatments.

a; Control b; Si c; GA3 d; Si+GA3 e; Heat f; Si+Heat g; GA3+Heat h; Si+GA3+Heat

onion day

The ANOVA Procedure

Class Level Information		
Class	Levels	Values
v	8	a b c d e f g h

Number of Observations Read	24
Number of Observations Used	24

Figure 5A

onion day

The ANOVA Procedure

Dependent Variable: y

Source	DF	Sum of Squares	Mean Square	F Value	Pr > F
Model	7	23.20420000	3.31488571	172.46	<.0001
Error	16	0.30753333	0.01922083		
Corrected Total	23	23.51173333			

R-Square	Coeff Var	Root MSE	y Mean
0.986920	6.835130	0.138639	2.028333

Source	DF	Anova SS	Mean Square	F Value	Pr > F
v	7	23.20420000	3.31488571	172.46	<.0001

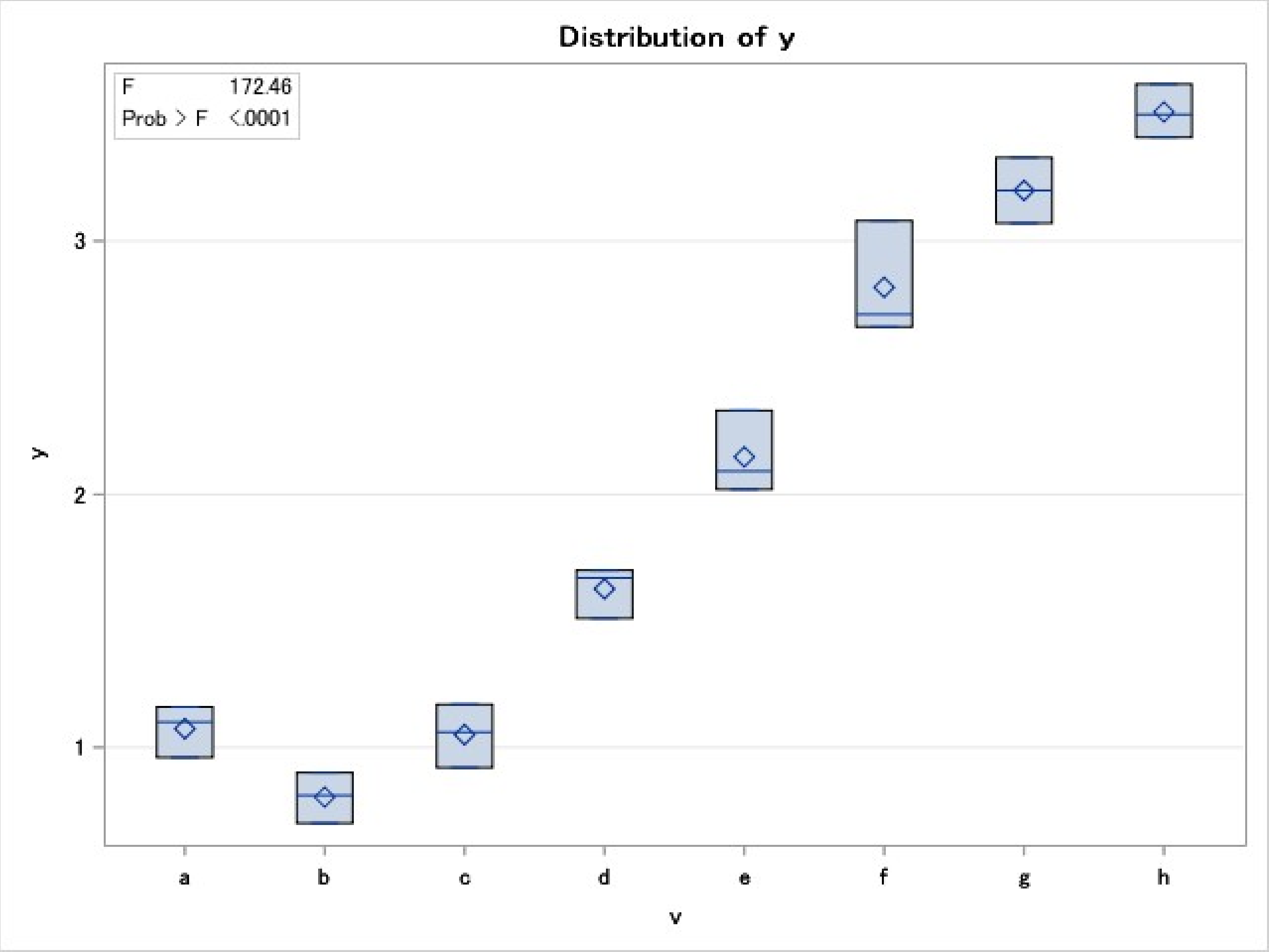

onion day

The ANOVA Procedure

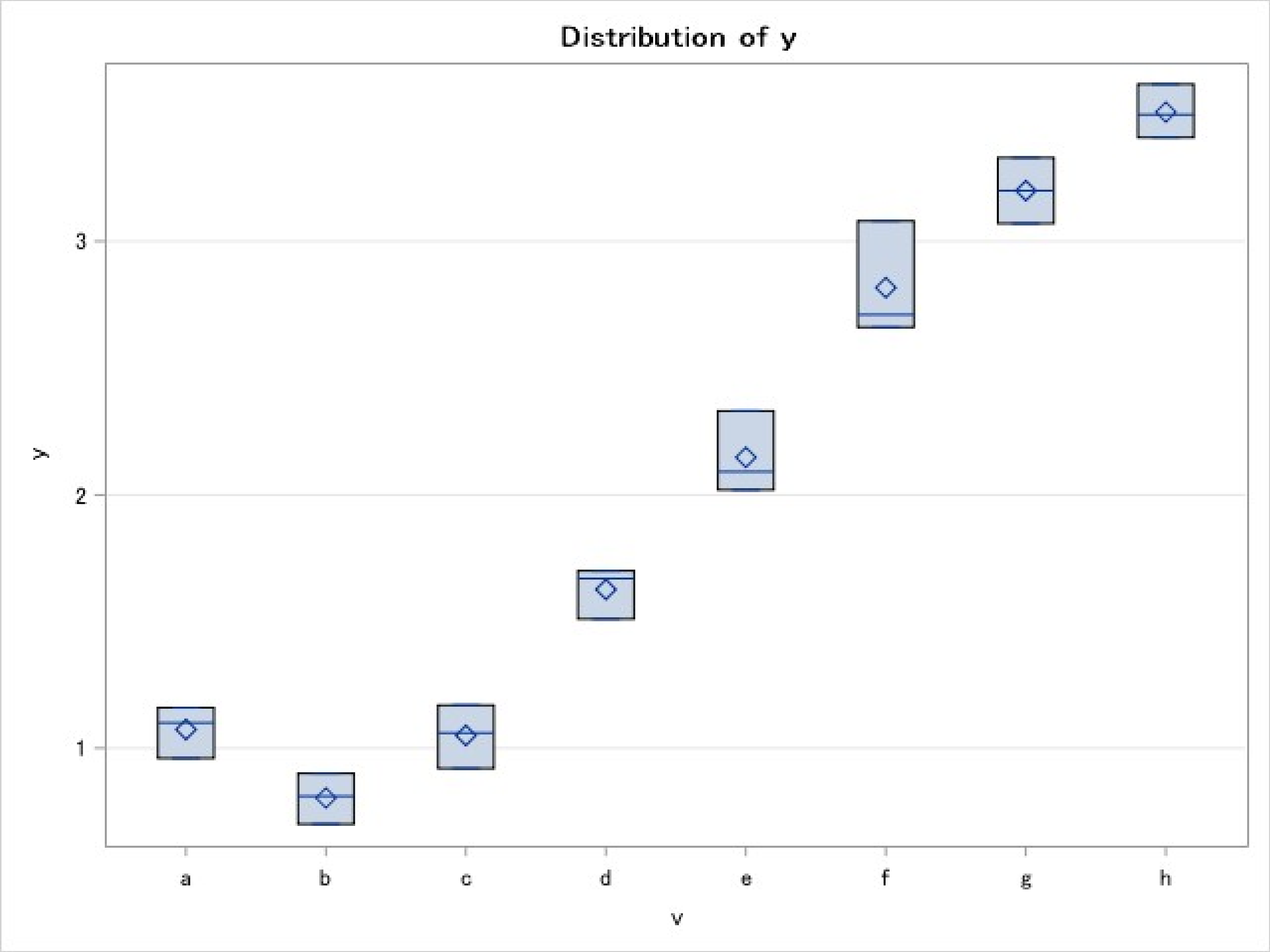

onion day

The ANOVA Procedure

Duncan's Multiple Range Test for y

Note: This test controls the Type I comparisonwise error rate, not the experimentwise error rate.

Alpha	0.05
Error Degrees of Freedom	16
Error Mean Square	0.019221

Number of Means	2	3	4	5	6	7	8
Critical Range	.2400	.2516	.2589	.2639	.2676	.2702	.2723

Means with the same letter are not significantly different.			
Duncan Grouping	Mean	N	v
A	3.5100	4	h
B	3.2000	4	g
C	2.8167	4	f
D	2.1467	4	e
E	1.6267	4	d
F	1.0733	4	a
F			
F	1.0500	4	c
G	0.8033	4	b

V describes the treatments.

a; Control b; Si c; GA3 d; Si+GA3 e; Heat f; Si+Heat g; GA3+Heat h; Si+GA3+Heat

Figure 5B

onion day

The ANOVA Procedure

Class Level Information		
Class	Levels	Values
v	8	a b c d e f g h

Number of Observations Read	24
Number of Observations Used	24

onion day

The ANOVA Procedure

Dependent Variable: y

Source	DF	Sum of Squares	Mean Square	F Value	Pr > F
Model	7	41.55040000	5.93577143	140.45	<.0001
Error	16	0.67620000	0.04226250		
Corrected Total	23	42.22660000			

R-Square	Coeff Var	Root MSE	y Mean
0.983986	7.656553	0.205578	2.685000

Source	DF	Anova SS	Mean Square	F Value	Pr > F
v	7	41.55040000	5.93577143	140.45	<.0001

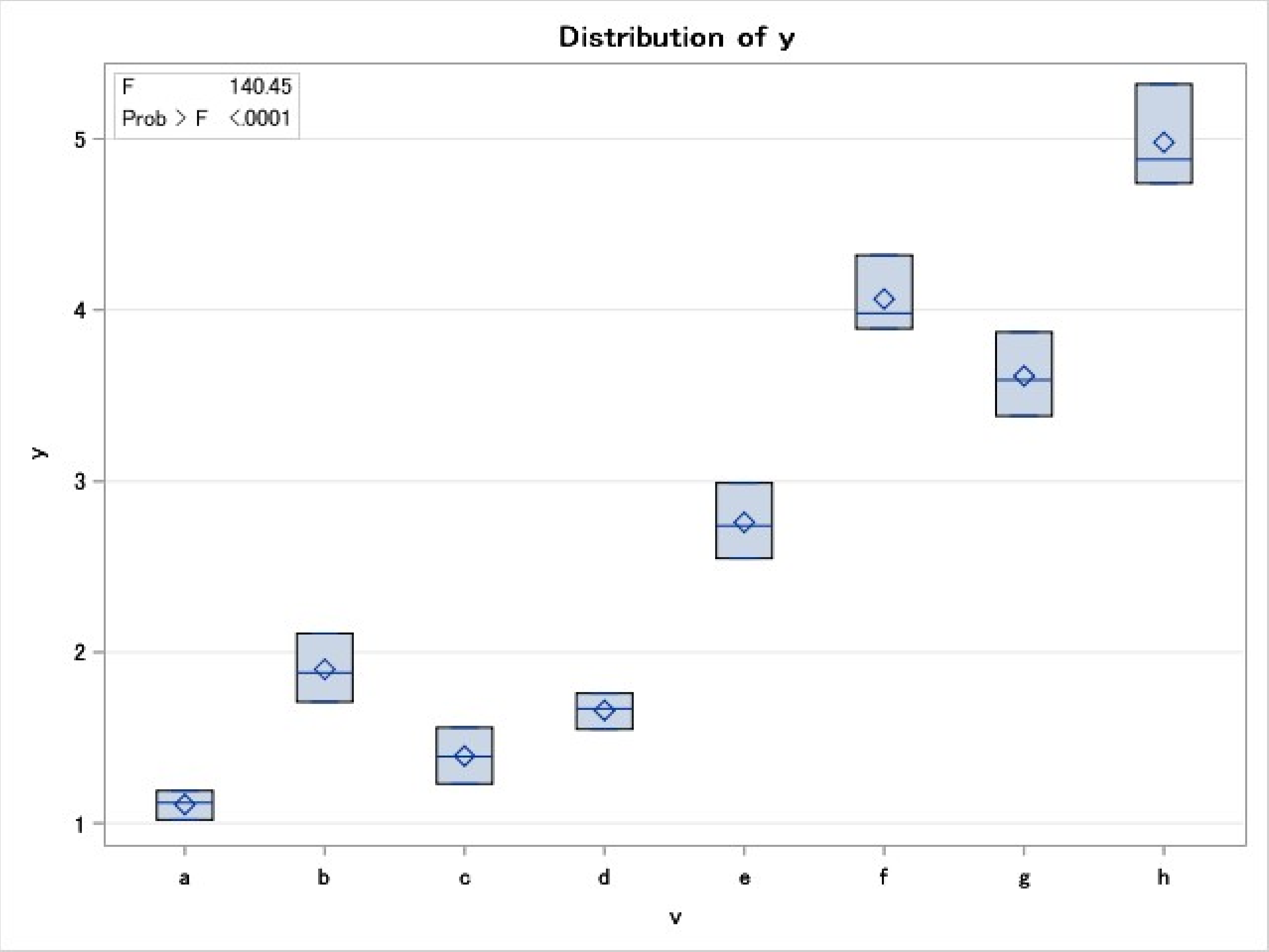

onion day

The ANOVA Procedure

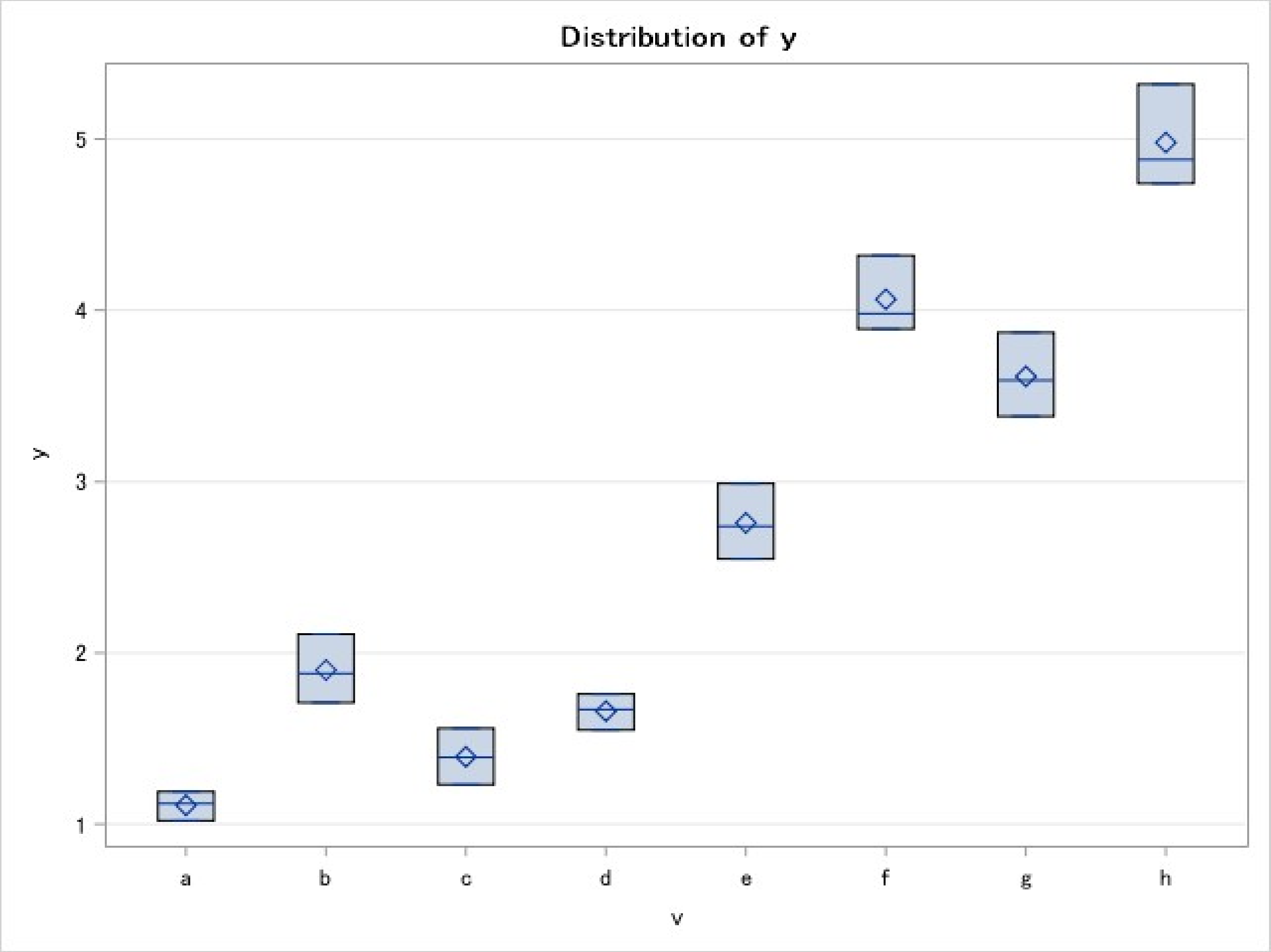

onion day

The ANOVA Procedure

Duncan's Multiple Range Test for y

Note: This test controls the Type I comparisonwise error rate, not the experimentwise error rate.

Alpha	0.05
Error Degrees of Freedom	16
Error Mean Square	0.042262

Number of Means	2	3	4	5	6	7	8
Critical Range	.3558	.3731	.3840	.3914	.3967	.4007	.4038

Means with the same letter are not significantly different.

Duncan Grouping		Mean	N	v
	A	4.9800	4	h
	B	4.0633	4	f
	C	3.6133	4	g
	D	2.7600	4	e
	E	1.9000	4	b
	E			
F	E	1.6600	4	d
F				
F	G	1.3933	4	c
	G			
	G	1.1100	4	a

V describes the treatments.

a; Control b; Si c; GA3 d; Si+GA3 e; Heat f; Si+Heat g; GA3+Heat h; Si+GA3+Heat

Figure 5C

onion day

The ANOVA Procedure

Class Level Information		
Class	Levels	Values
v	8	a b c d e f g h

Number of Observations Read	24
Number of Observations Used	24

onion day

The ANOVA Procedure

Dependent Variable: y

Source	DF	Sum of Squares	Mean Square	F Value	Pr > F
Model	7	44.46162917	6.35166131	93.34	<.0001
Error	16	1.08873333	0.06804583		
Corrected Total	23	45.55036250			

R-Square	Coeff Var	Root MSE	y Mean
0.976098	15.31069	0.260856	1.703750

Source	DF	Anova SS	Mean Square	F Value	Pr > F
v	7	44.46162917	6.35166131	93.34	<.0001

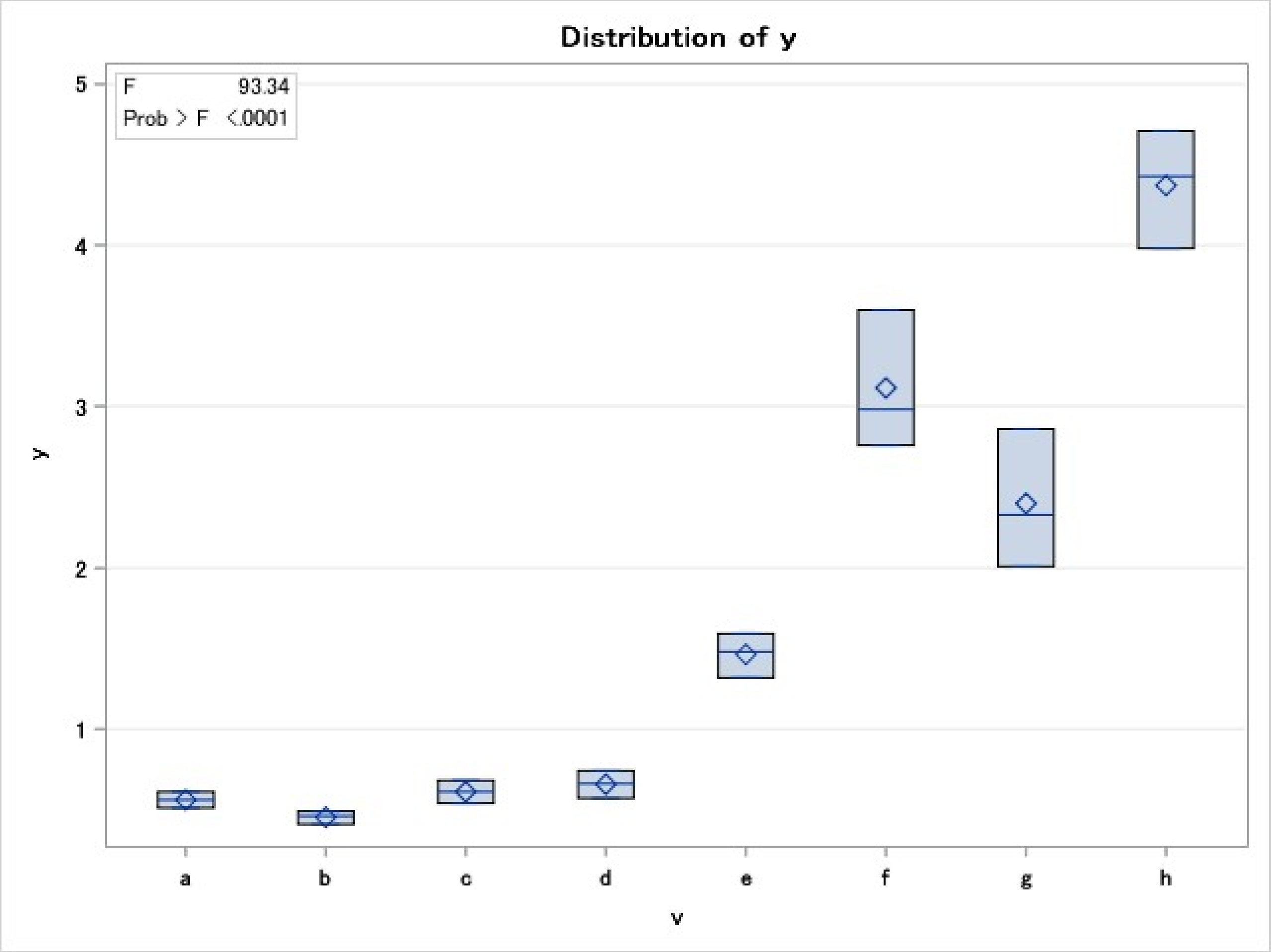

onion day

The ANOVA Procedure

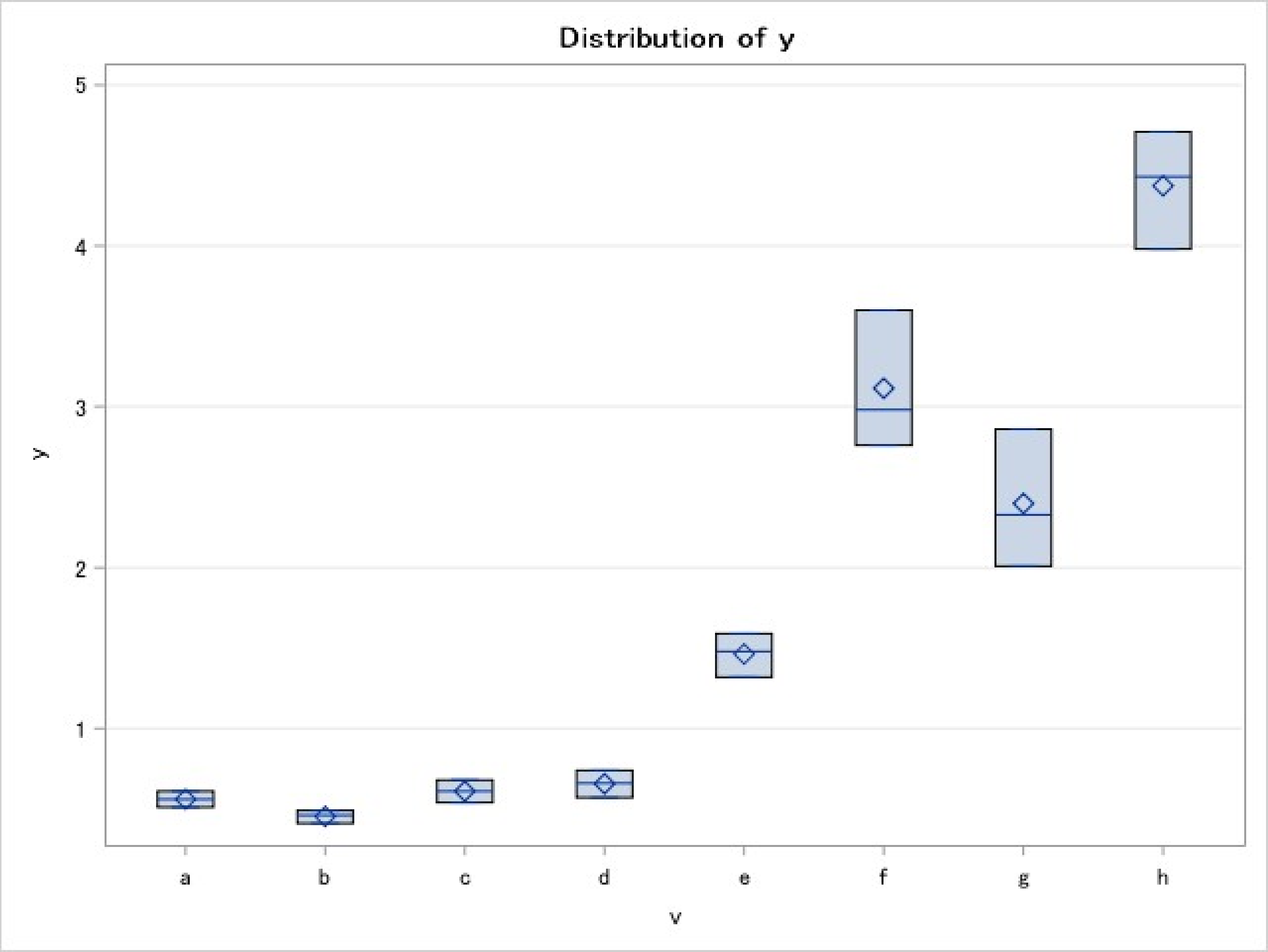

onion day

The ANOVA Procedure

Duncan's Multiple Range Test for y

Note: This test controls the Type I comparisonwise error rate, not the experimentwise error rate.

Alpha	0.05
Error Degrees of Freedom	16
Error Mean Square	0.068046

Number of Means	2	3	4	5	6	7	8
Critical Range	.4515	.4735	.4872	.4966	.5034	.5085	.5123

Means with the same letter are not significantly different.			
Duncan Grouping	Mean	N	v
A	4.3733	4	h
B	3.1133	4	f
C	2.4000	4	g
D	1.4633	4	e
E	0.6567	4	d
E			
E	0.6100	4	c
E			
E	0.5600	4	a
E			
E	0.4533	4	b

V describes the treatments.

a; Control b; Si c; GA3 d; Si+GA3 e; Heat f; Si+Heat g; GA3+Heat h; Si+GA3+Heat

Figure 5D

onion day

The ANOVA Procedure

Class Level Information		
Class	Levels	Values
v	8	a b c d e f g h

Number of Observations Read	24
Number of Observations Used	24

onion day

The ANOVA Procedure

Dependent Variable: y

Source	DF	Sum of Squares	Mean Square	F Value	Pr > F
Model	7	35.97300000	5.13900000	170.19	<.0001
Error	16	0.48313333	0.03019583		
Corrected Total	23	36.45613333			

R-Square	Coeff Var	Root MSE	y Mean
0.986748	7.728813	0.173769	2.248333

Source	DF	Anova SS	Mean Square	F Value	Pr > F
v	7	35.97300000	5.13900000	170.19	<.0001

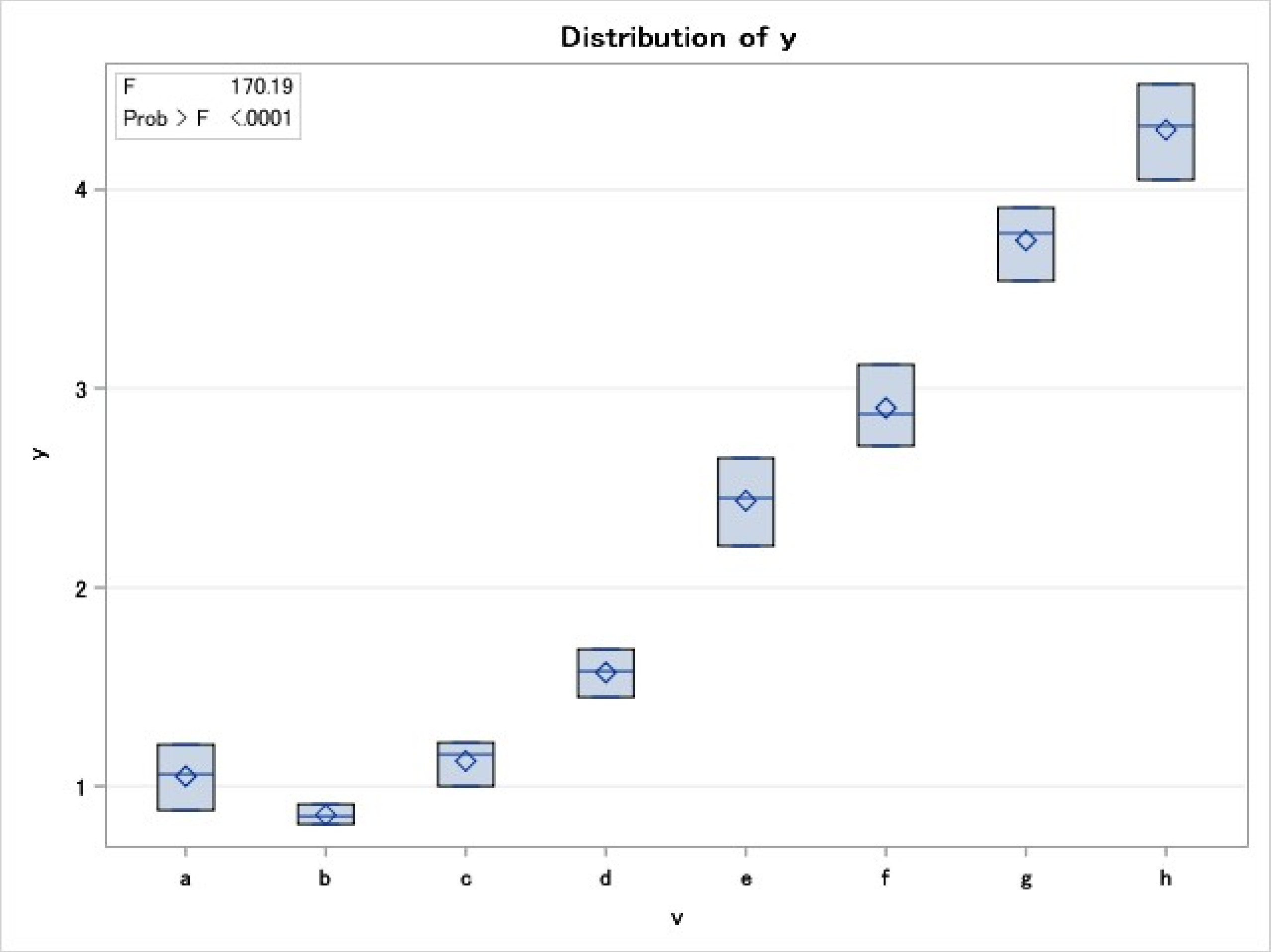

onion day

The ANOVA Procedure

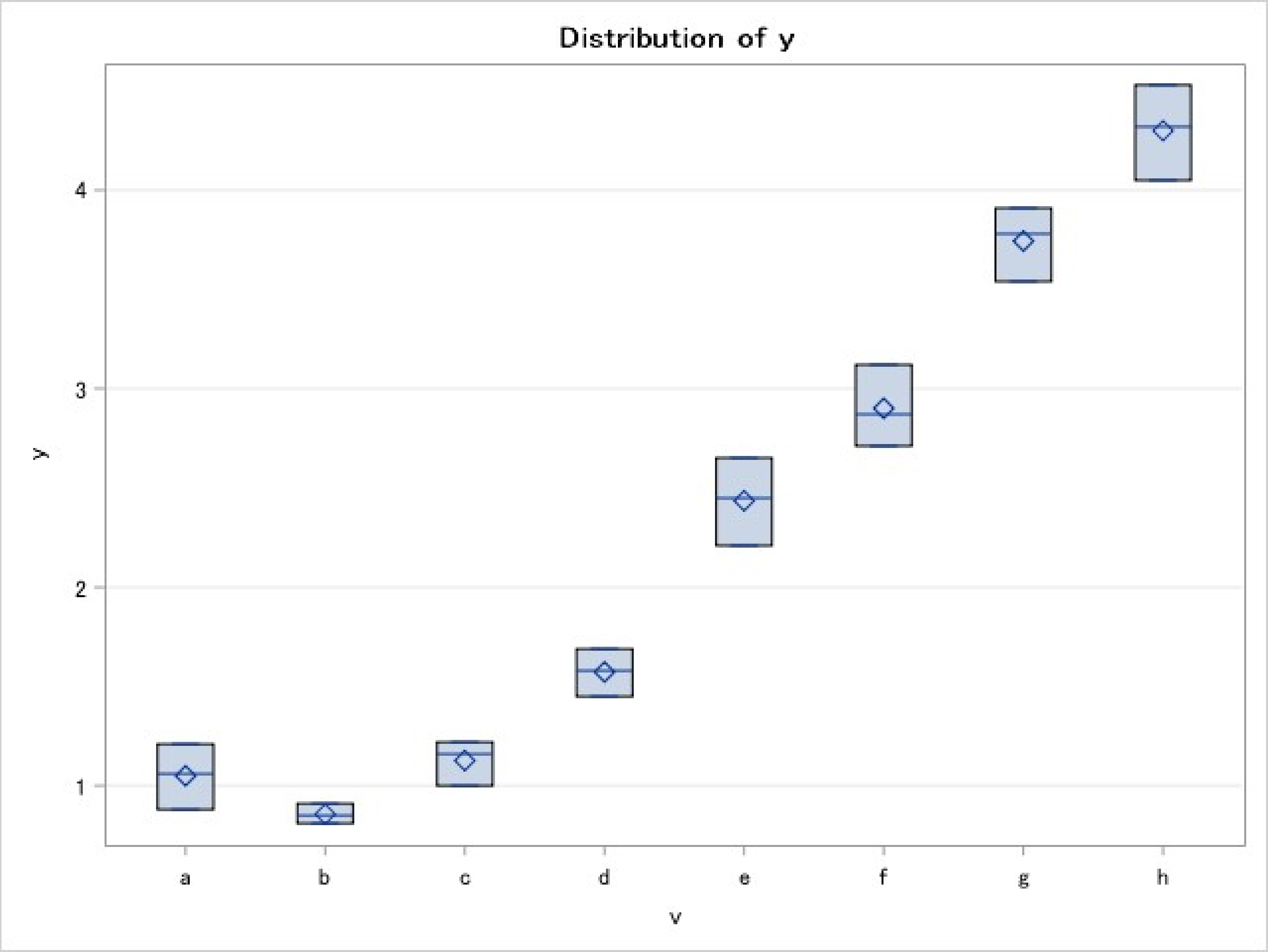

onion day

The ANOVA Procedure

Duncan's Multiple Range Test for y

Note: This test controls the Type I comparisonwise error rate, not the experimentwise error rate.

Alpha	0.05
Error Degrees of Freedom	16
Error Mean Square	0.030196

Number of Means	2	3	4	5	6	7	8
Critical Range	.3008	.3154	.3245	.3308	.3353	.3387	.3413

Means with the same letter are not significantly different.			
Duncan Grouping	Mean	N	v
A	4.3000	4	h
B	3.7433	4	g
C	2.9000	4	f
D	2.4367	4	e
E	1.5733	4	d
F	1.1267	4	c
F			
F	1.0500	4	a
F			
F	0.8567	4	b

V describes the treatments.

a; Control b; Si c; GA3 d; Si+GA3 e; Heat f; Si+Heat g; GA3+Heat h; Si+GA3+Heat

onion day

The ANOVA Procedure

Class Level Information		
Class	Levels	Values
v	8	a b c d e f g h

Number of Observations Read	24
Number of Observations Used	24

Figure 6A

onion day

The ANOVA Procedure

Dependent Variable: y

Source	DF	Sum of Squares	Mean Square	F Value	Pr > F
Model	7	396.4985292	56.6426470	144.83	<.0001
Error	16	6.2576667	0.3911042		
Corrected Total	23	402.7561958			

R-Square	Coeff Var	Root MSE	y Mean
0.984463	9.933945	0.625383	6.295417

Source	DF	Anova SS	Mean Square	F Value	Pr > F
v	7	396.4985292	56.6426470	144.83	<.0001

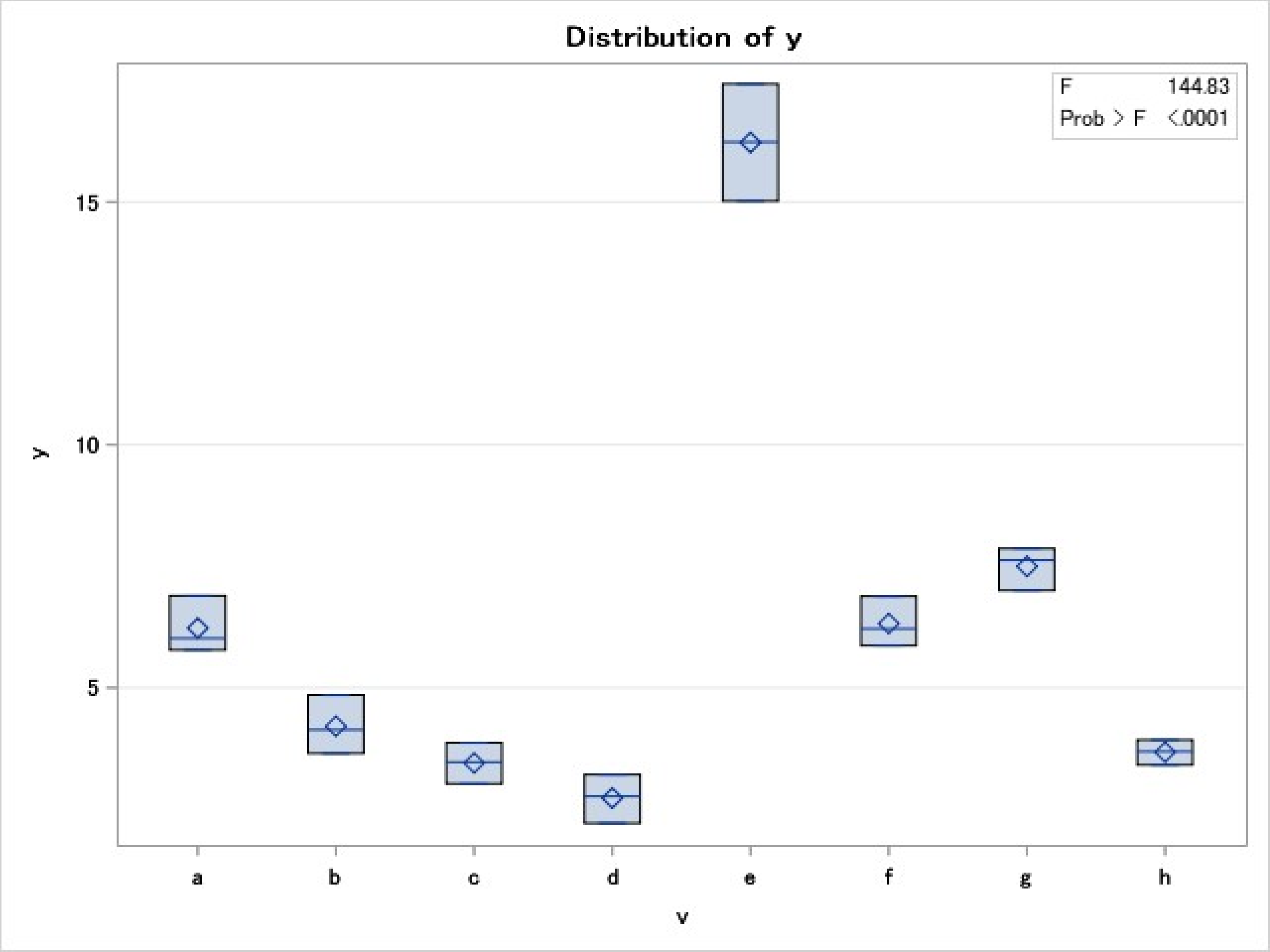

onion day

The ANOVA Procedure

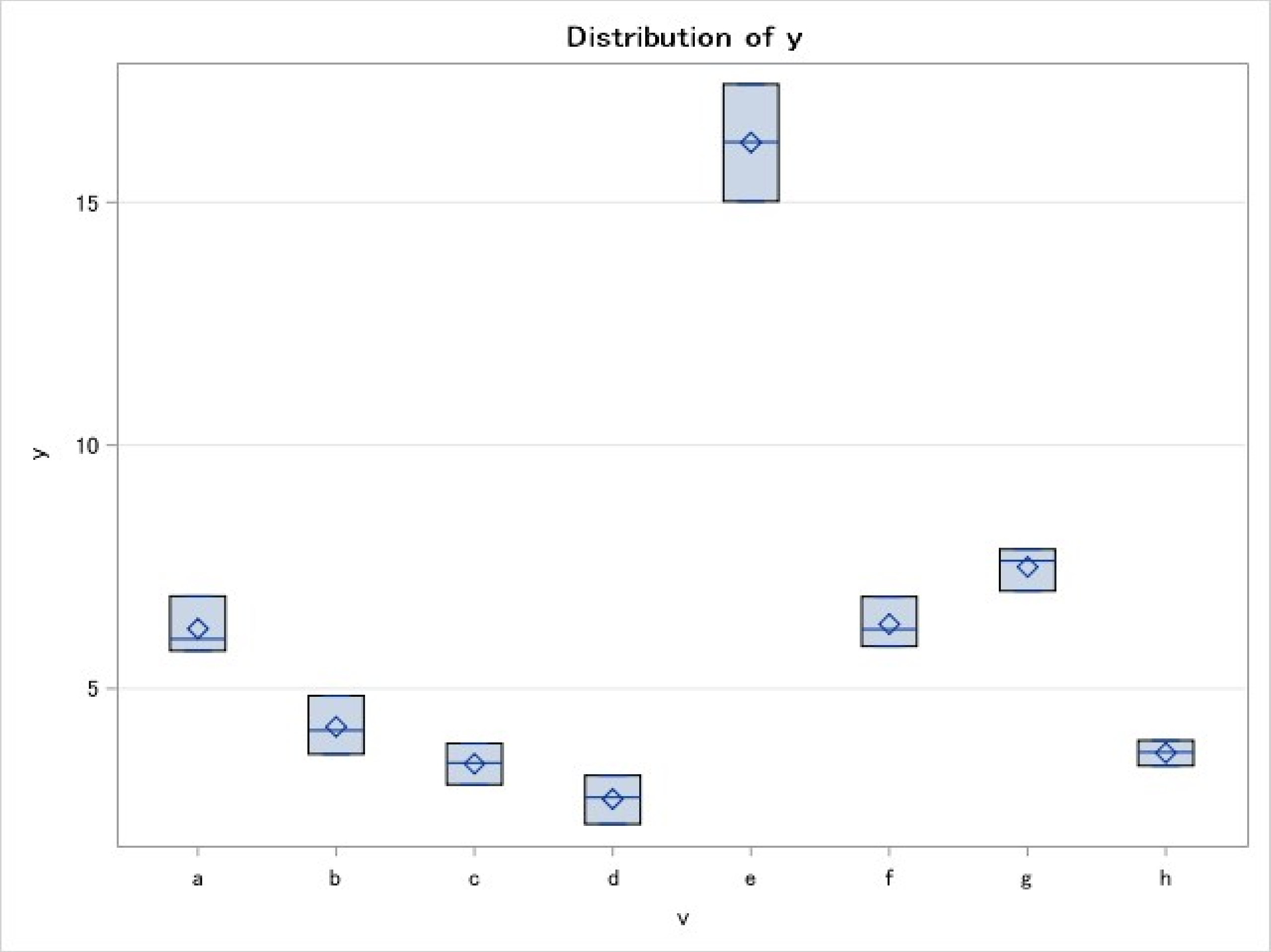

onion day

The ANOVA Procedure

Duncan's Multiple Range Test for y

Note: This test controls the Type I comparisonwise error rate, not the experimentwise error rate.

Alpha	0.05
Error Degrees of Freedom	16
Error Mean Square	0.391104

Number of Means	2	3	4	5	6	7	8
Critical Range	1.082	1.135	1.168	1.191	1.207	1.219	1.228

Means with the same letter are not significantly different.

Duncan Grouping		Mean	N	v
	A	16.2300	4	e
	B	7.5033	4	g
	C	6.3267	4	f
	C			
	C	6.2333	4	a
	D	4.2133	4	b
	D			
E	D	3.6767	4	h
E	D			
E	D	3.4533	4	c
E				
E		2.7267	4	d

V describes the treatments.

a; Control b; Si c; GA3 d; Si+GA3 e; Heat f; Si+Heat g; GA3+Heat h; Si+GA3+Heat

Figure 6B

onion day

The ANOVA Procedure

Class Level Information		
Class	Levels	Values
v	8	a b c d e f g h

Number of Observations Read	24
Number of Observations Used	24

onion day

The ANOVA Procedure

Dependent Variable: y

Source	DF	Sum of Squares	Mean Square	F Value	Pr > F
Model	7	34.54156250	4.93450893	88.83	<.0001
Error	16	0.88880000	0.05555000		
Corrected Total	23	35.43036250			

R-Square	Coeff Var	Root MSE	y Mean
0.974914	9.887382	0.235690	2.383750

Source	DF	Anova SS	Mean Square	F Value	Pr > F
v	7	34.54156250	4.93450893	88.83	<.0001

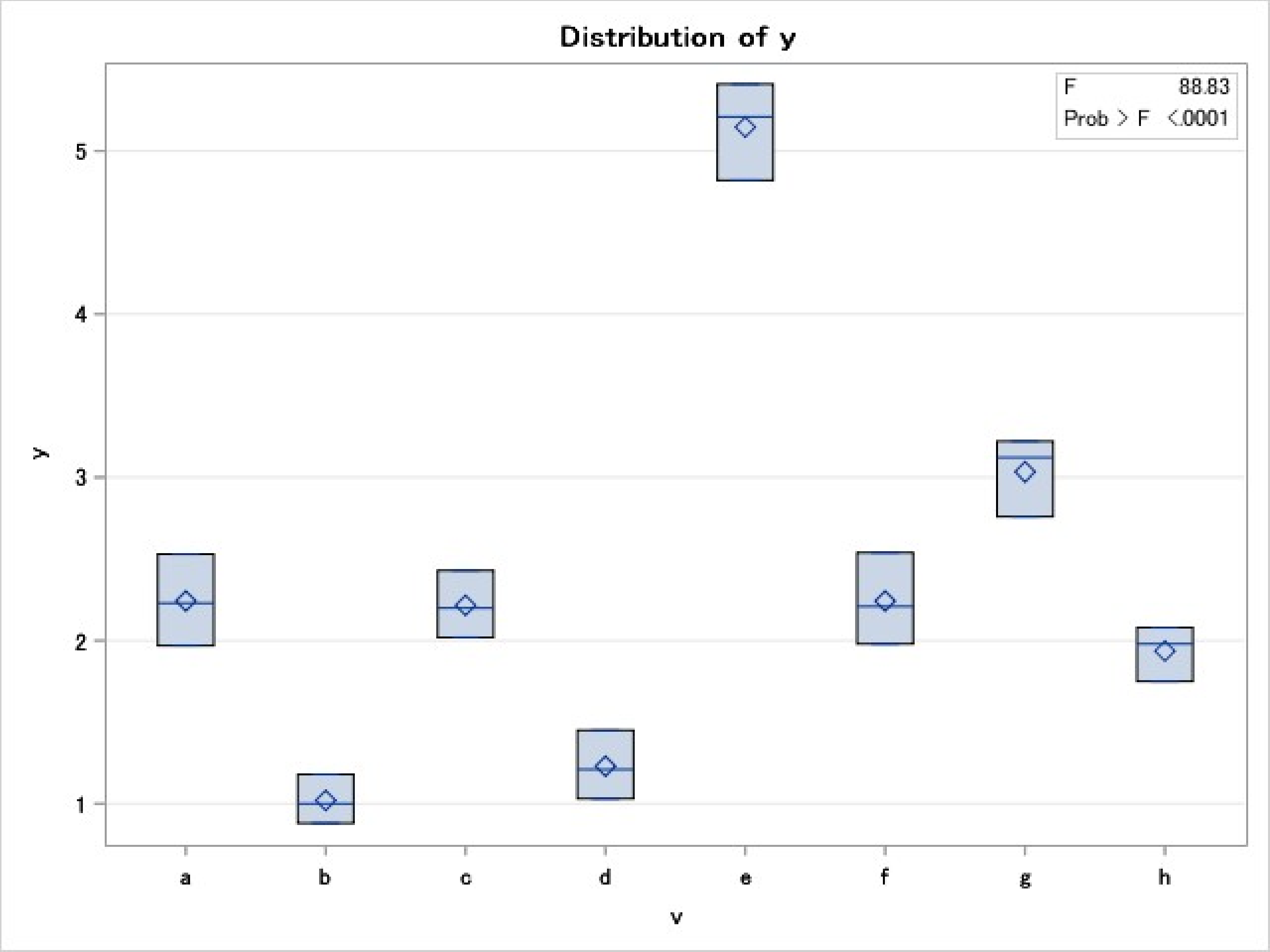

onion day

The ANOVA Procedure

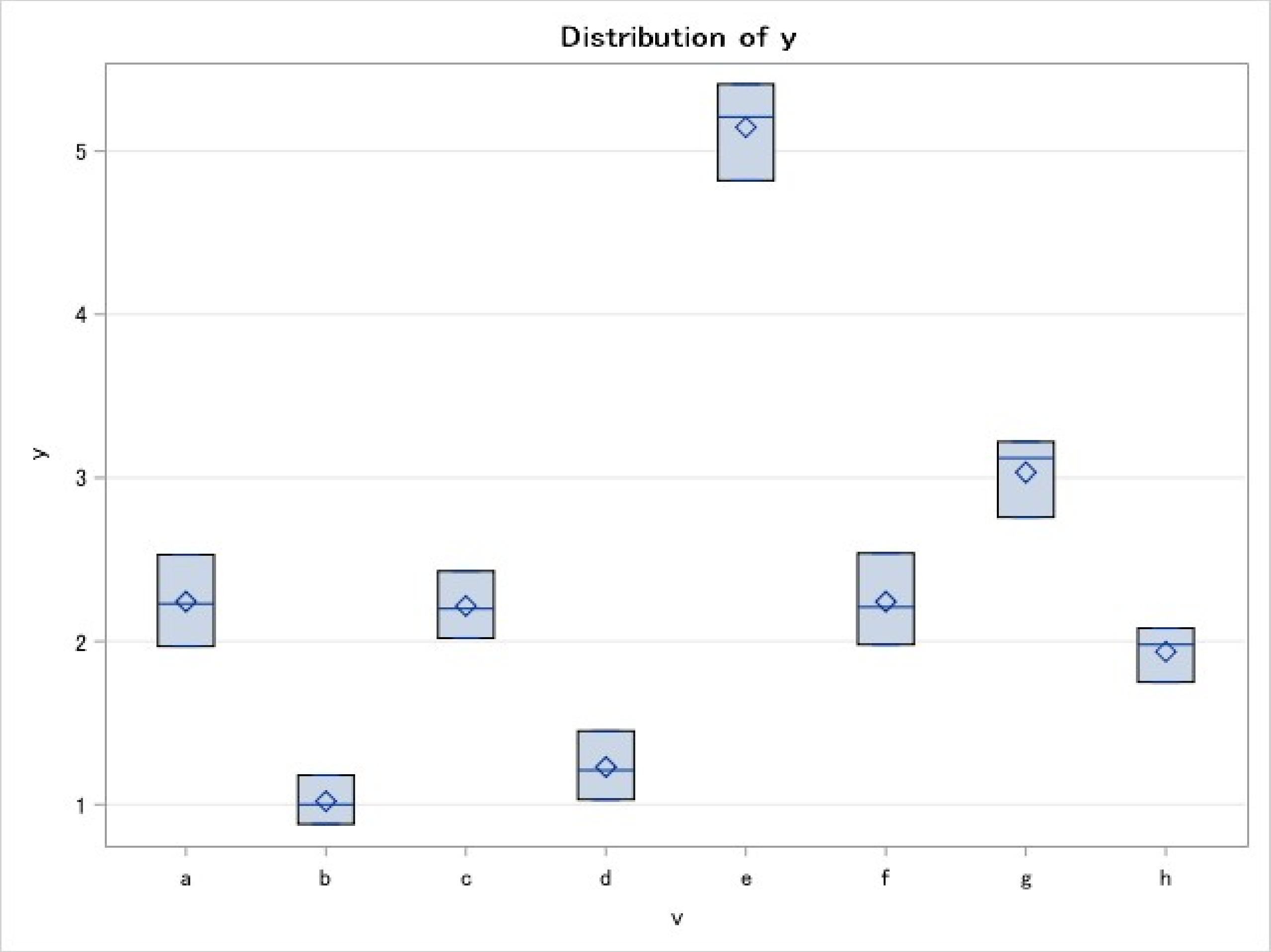

onion day

The ANOVA Procedure

Duncan's Multiple Range Test for y

Note: This test controls the Type I comparisonwise error rate, not the experimentwise error rate.

Alpha	0.05
Error Degrees of Freedom	16
Error Mean Square	0.05555

Number of Means	2	3	4	5	6	7	8
Critical Range	.4079	.4278	.4402	.4487	.4548	.4594	.4629

Means with the same letter are not significantly different.			
Duncan Grouping	Mean	N	v
A	5.1467	4	e
B	3.0333	4	g
C	2.2433	4	a
C			
C	2.2433	4	f
C			
C	2.2167	4	c
C			
C	1.9367	4	h
D	1.2300	4	d
D			
D	1.0200	4	b

V describes the treatments.

a; Control b; Si c; GA3 d; Si+GA3 e; Heat f; Si+Heat g; GA3+Heat h; Si+GA3+Heat

Figure 6C

onion day

The ANOVA Procedure

Class Level Information		
Class	Levels	Values
v	8	a b c d e f g h

Number of Observations Read	24
Number of Observations Used	24

onion day

The ANOVA Procedure

Dependent Variable: y

Source	DF	Sum of Squares	Mean Square	F Value	Pr > F
Model	7	2.67322917	0.38188988	10.00	<.0001
Error	16	0.61106667	0.03819167		
Corrected Total	23	3.28429583			

R-Square	Coeff Var	Root MSE	y Mean
0.813943	32.19111	0.195427	0.607083

Source	DF	Anova SS	Mean Square	F Value	Pr > F
v	7	2.67322917	0.38188988	10.00	<.0001

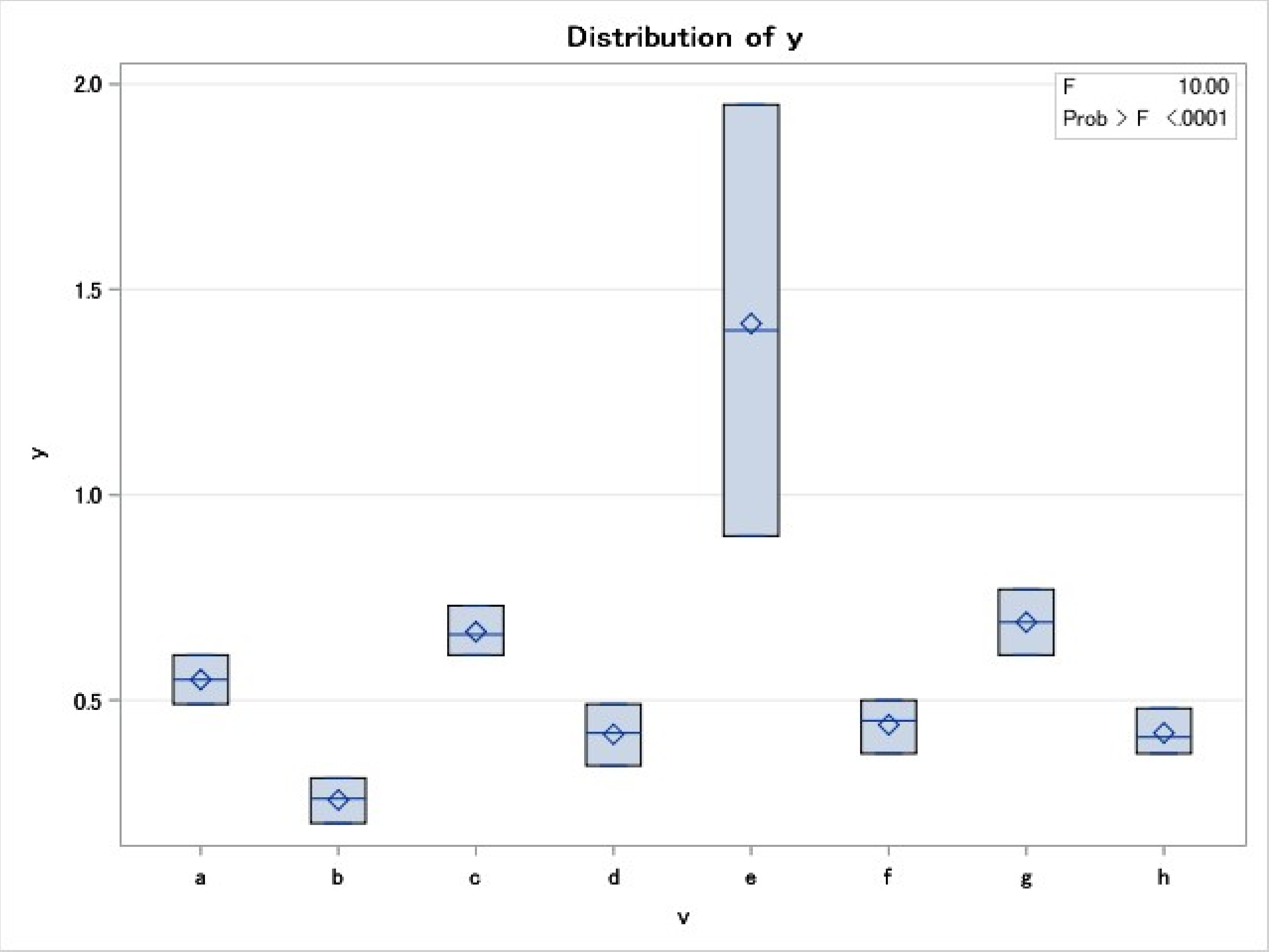

onion day

The ANOVA Procedure

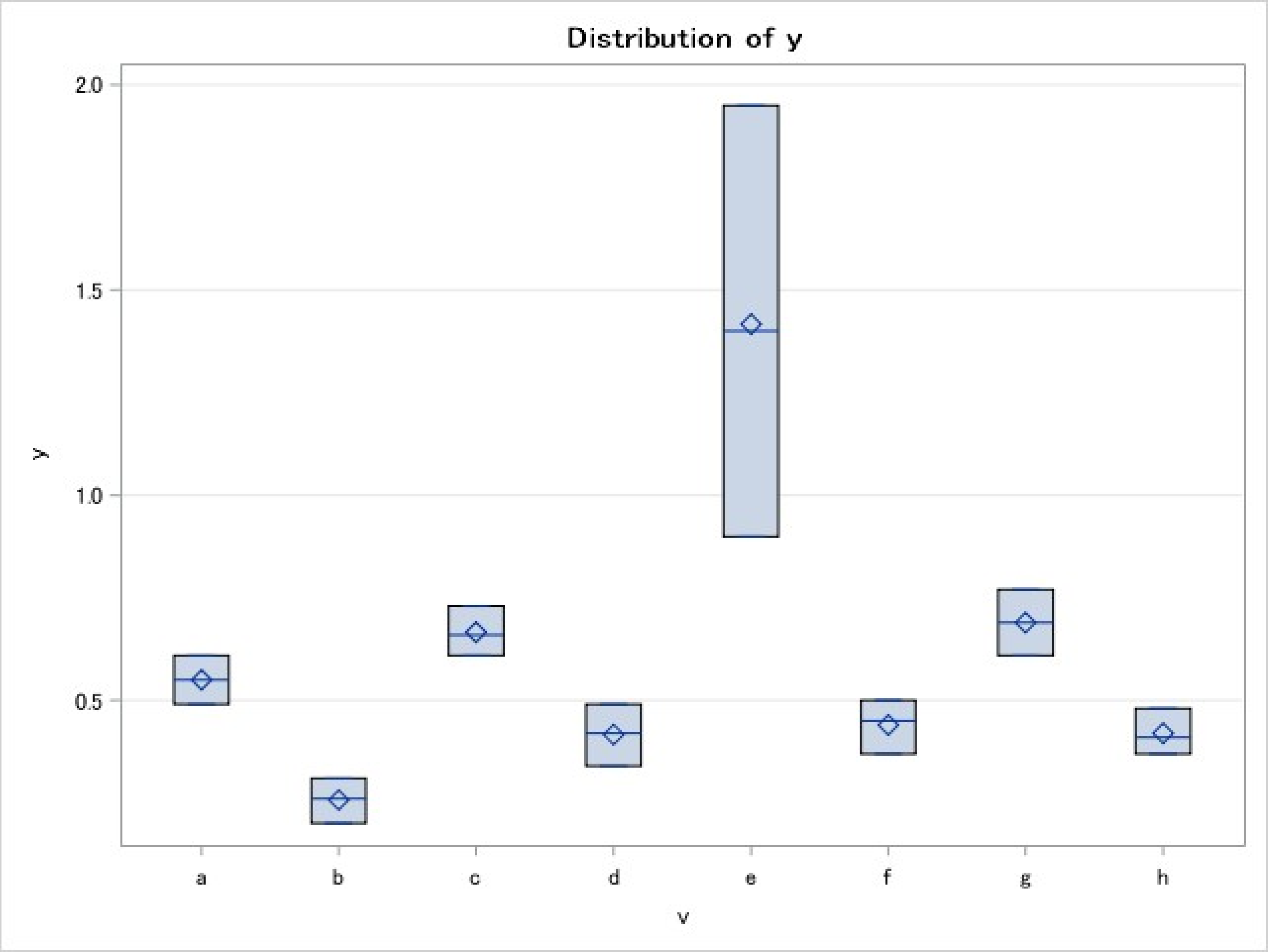

onion day

The ANOVA Procedure

Duncan's Multiple Range Test for y

Note: This test controls the Type I comparisonwise error rate, not the experimentwise error rate.

Alpha	0.05
Error Degrees of Freedom	16
Error Mean Square	0.038192

Number of Means	2	3	4	5	6	7	8
Critical Range	.3382	.3547	.3650	.3720	.3771	.3809	.3838

Means with the same letter are not significantly different.

Duncan Grouping		Mean	N	v
	A	1.4167	4	e
	B	0.6900	4	g
	B			
	B	0.6667	4	c
	B			
C	B	0.5500	4	a
C	B			
C	B	0.4400	4	f
C	B			
C	B	0.4200	4	h
C	B			
C	B	0.4167	4	d
C				
C		0.2567	4	b

V describes the treatments.

a; Control b; Si c; GA3 d; Si+GA3 e; Heat f; Si+Heat g; GA3+Heat h; Si+GA3+Heat

Figure 6D

onion day

The ANOVA Procedure

Class Level Information		
Class	Levels	Values
v	8	a b c d e f g h

Number of Observations Read	24
Number of Observations Used	24

onion day

The ANOVA Procedure

Dependent Variable: y

Source	DF	Sum of Squares	Mean Square	F Value	Pr > F
Model	7	45.07779583	6.43968512	91.16	<.0001
Error	16	1.13026667	0.07064167		
Corrected Total	23	46.20806250			

R-Square	Coeff Var	Root MSE	y Mean
0.975540	8.768165	0.265785	3.031250

Source	DF	Anova SS	Mean Square	F Value	Pr > F
v	7	45.07779583	6.43968512	91.16	<.0001

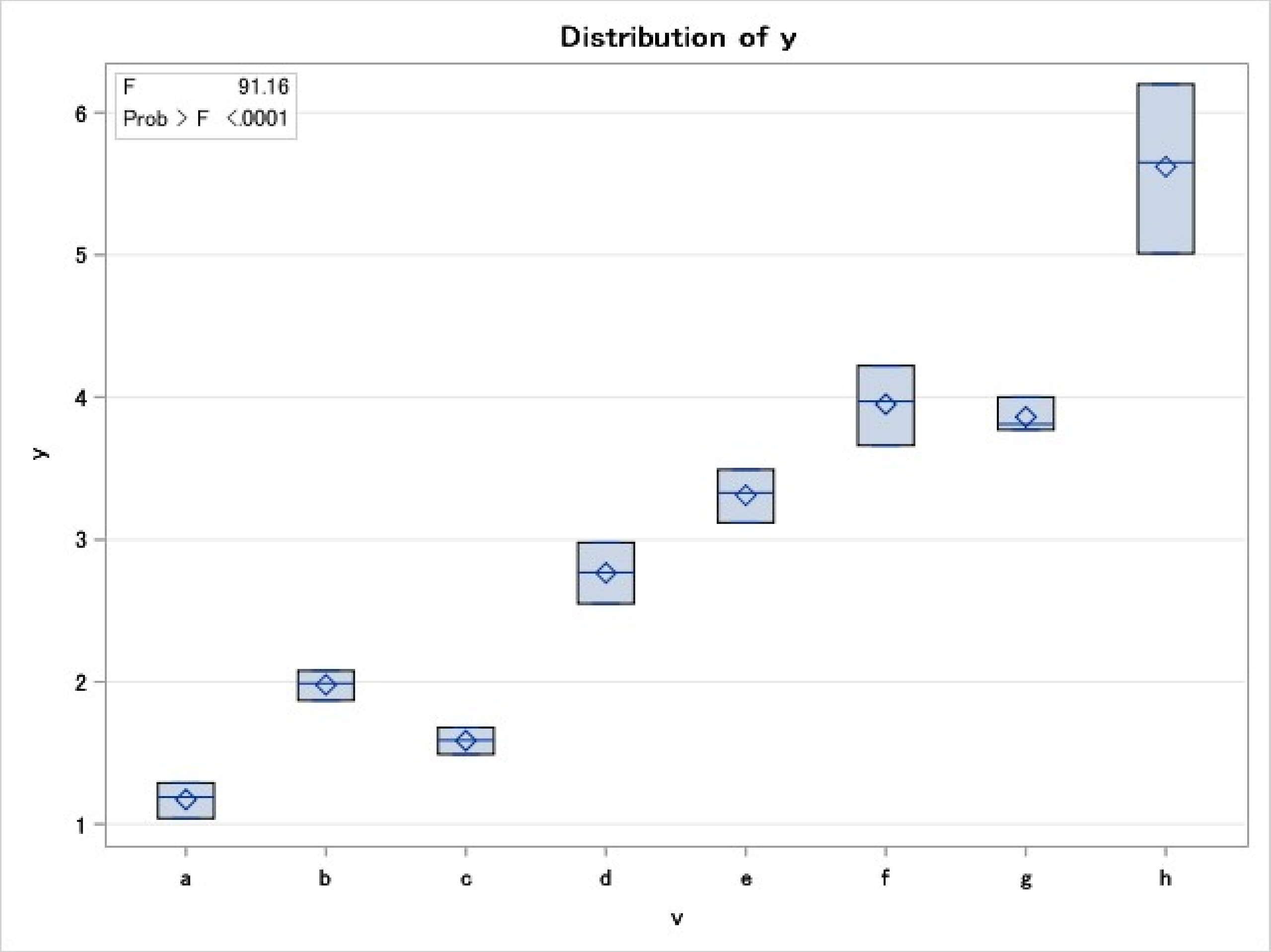

onion day

The ANOVA Procedure

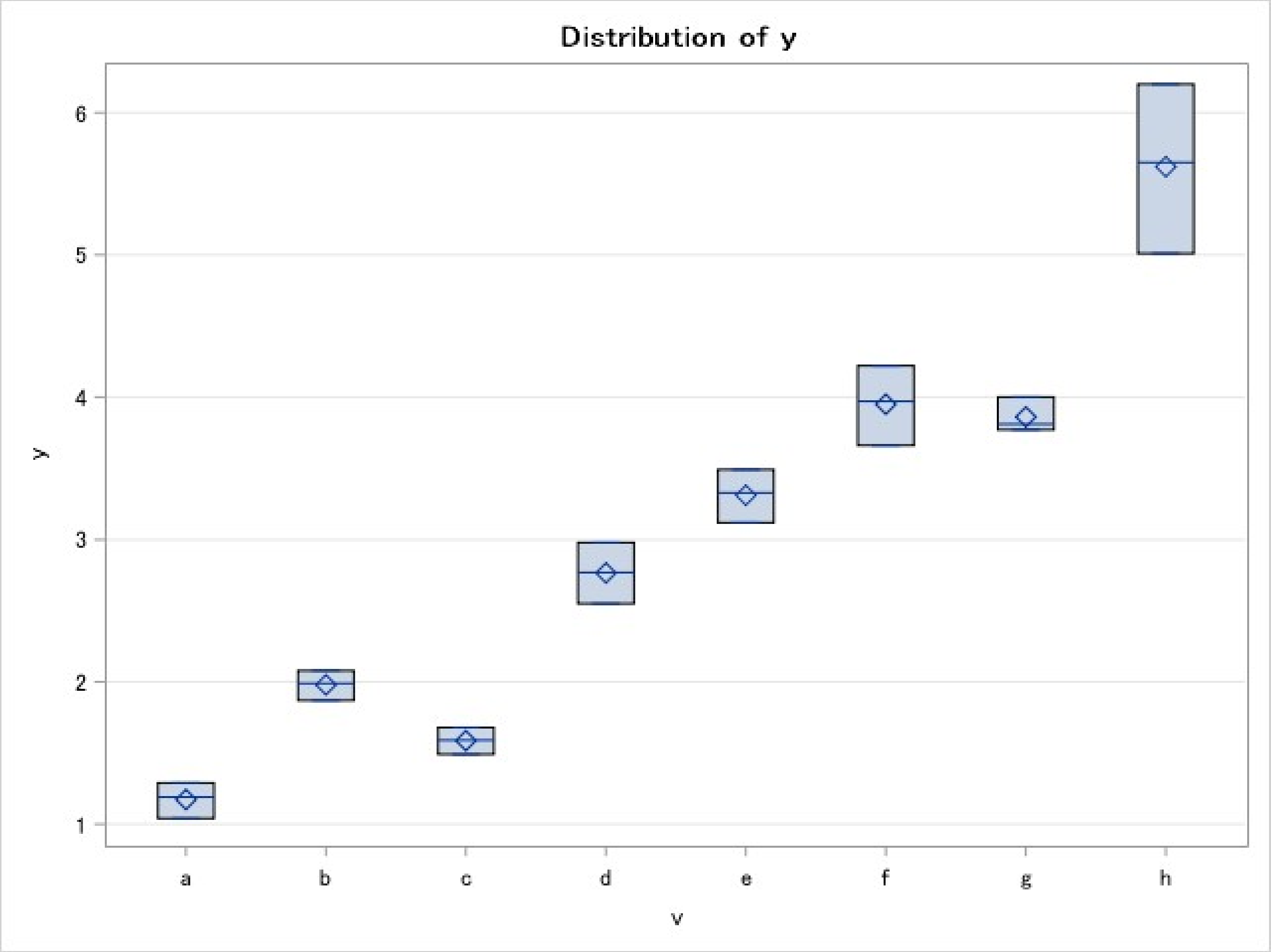

onion day

The ANOVA Procedure

Duncan's Multiple Range Test for y

Note: This test controls the Type I comparisonwise error rate, not the experimentwise error rate.

Alpha	0.05
Error Degrees of Freedom	16
Error Mean Square	0.070642

Number of Means	2	3	4	5	6	7	8
Critical Range	.4600	.4824	.4964	.5060	.5129	.5181	.5220

Means with the same letter are not significantly different.

Duncan Grouping		Mean	N	v
	A	5.6200	4	h
	B	3.9500	4	f
	B			
	B	3.8600	4	g
	C	3.3133	4	e
	D	2.7667	4	d
	E	1.9800	4	b
	E			
F	E	1.5867	4	c
F				
F		1.1733	4	a

V describes the treatments.

a; Control b; Si c; GA3 d; Si+GA3 e; Heat f; Si+Heat g; GA3+Heat h; Si+GA3+Heat

Figure 6E

onion day

The ANOVA Procedure

Class Level Information		
Class	Levels	Values
v	8	a b c d e f g h

Number of Observations Read	24
Number of Observations Used	24

onion day

The ANOVA Procedure

Dependent Variable: y

Source	DF	Sum of Squares	Mean Square	F Value	Pr > F
Model	7	88.58800000	12.65542857	144.88	<.0001
Error	16	1.39760000	0.08735000		
Corrected Total	23	89.98560000			

R-Square	Coeff Var	Root MSE	y Mean
0.984469	12.06328	0.295550	2.450000

Source	DF	Anova SS	Mean Square	F Value	Pr > F
v	7	88.58800000	12.65542857	144.88	<.0001

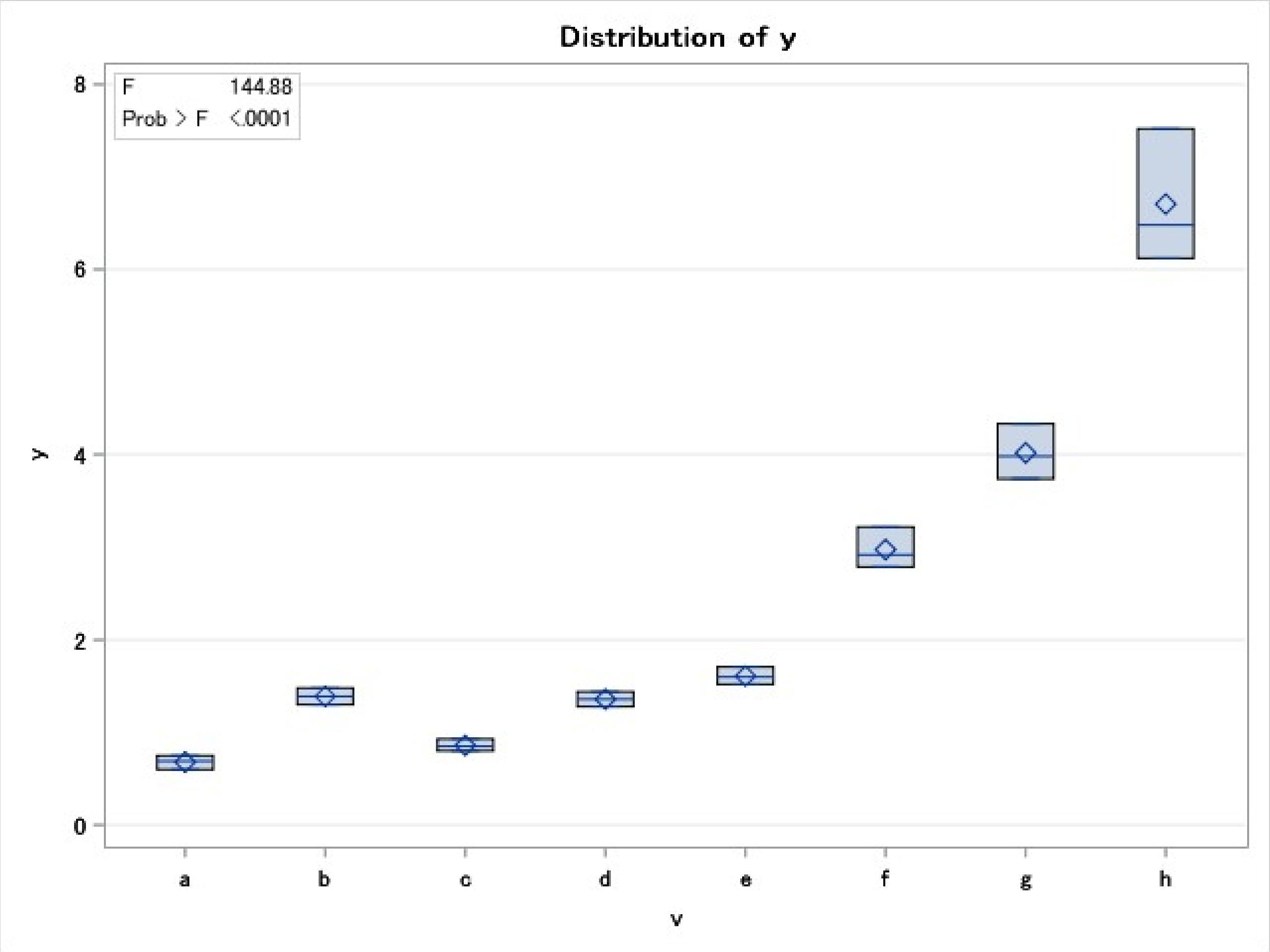

onion day

The ANOVA Procedure

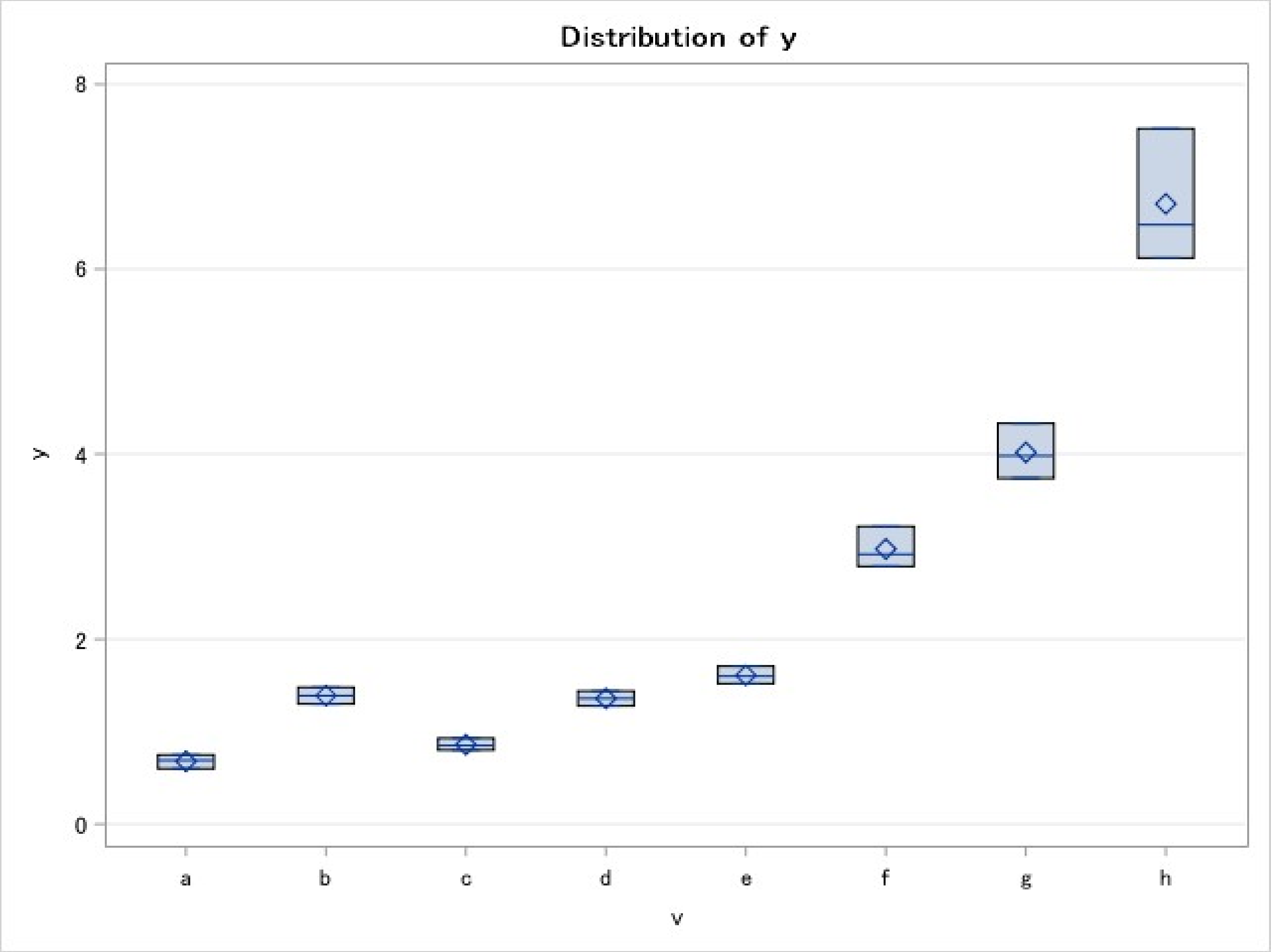

onion day

The ANOVA Procedure

Duncan's Multiple Range Test for y

Note: This test controls the Type I comparisonwise error rate, not the experimentwise error rate.

Alpha	0.05
Error Degrees of Freedom	16
Error Mean Square	0.08735

Number of Means	2	3	4	5	6	7	8
Critical Range	.5115	.5364	.5520	.5627	.5704	.5761	.5805

Means with the same letter are not significantly different.

Duncan Grouping		Mean	N	v
	A	6.7067	4	h
	B	4.0167	4	g
	C	2.9767	4	f
	D	1.6100	4	e
	D			
E	D	1.3900	4	b
E	D			
E	D	1.3600	4	d
E				
E	F	0.8600	4	c
	F			
	F	0.6800	4	a

V describes the treatments.

a; Control b; Si c; GA3 d; Si+GA3 e; Heat f; Si+Heat g; GA3+Heat h; Si+GA3+Heat

Figure 6F

onion day

The ANOVA Procedure

Class Level Information		
Class	Levels	Values
v	8	a b c d e f g h

Number of Observations Read	24
Number of Observations Used	24

onion day

The ANOVA Procedure

Dependent Variable: y

Source	DF	Sum of Squares	Mean Square	F Value	Pr > F
Model	7	41.66886667	5.95269524	99.37	<.0001
Error	16	0.95846667	0.05990417		
Corrected Total	23	42.62733333			

R-Square	Coeff Var	Root MSE	y Mean
0.977515	12.21730	0.244753	2.003333

Source	DF	Anova SS	Mean Square	F Value	Pr > F
v	7	41.66886667	5.95269524	99.37	<.0001

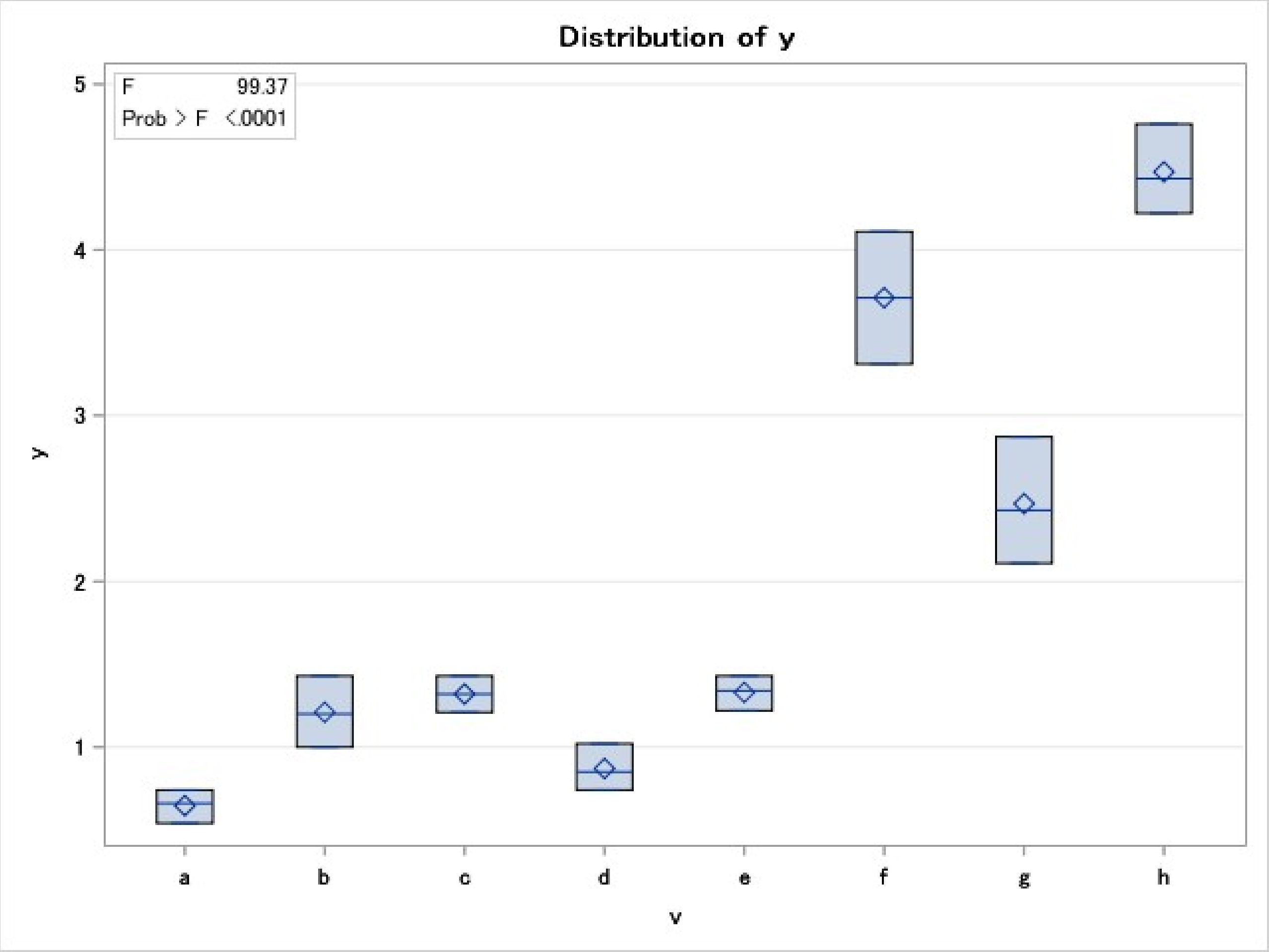

onion day

The ANOVA Procedure

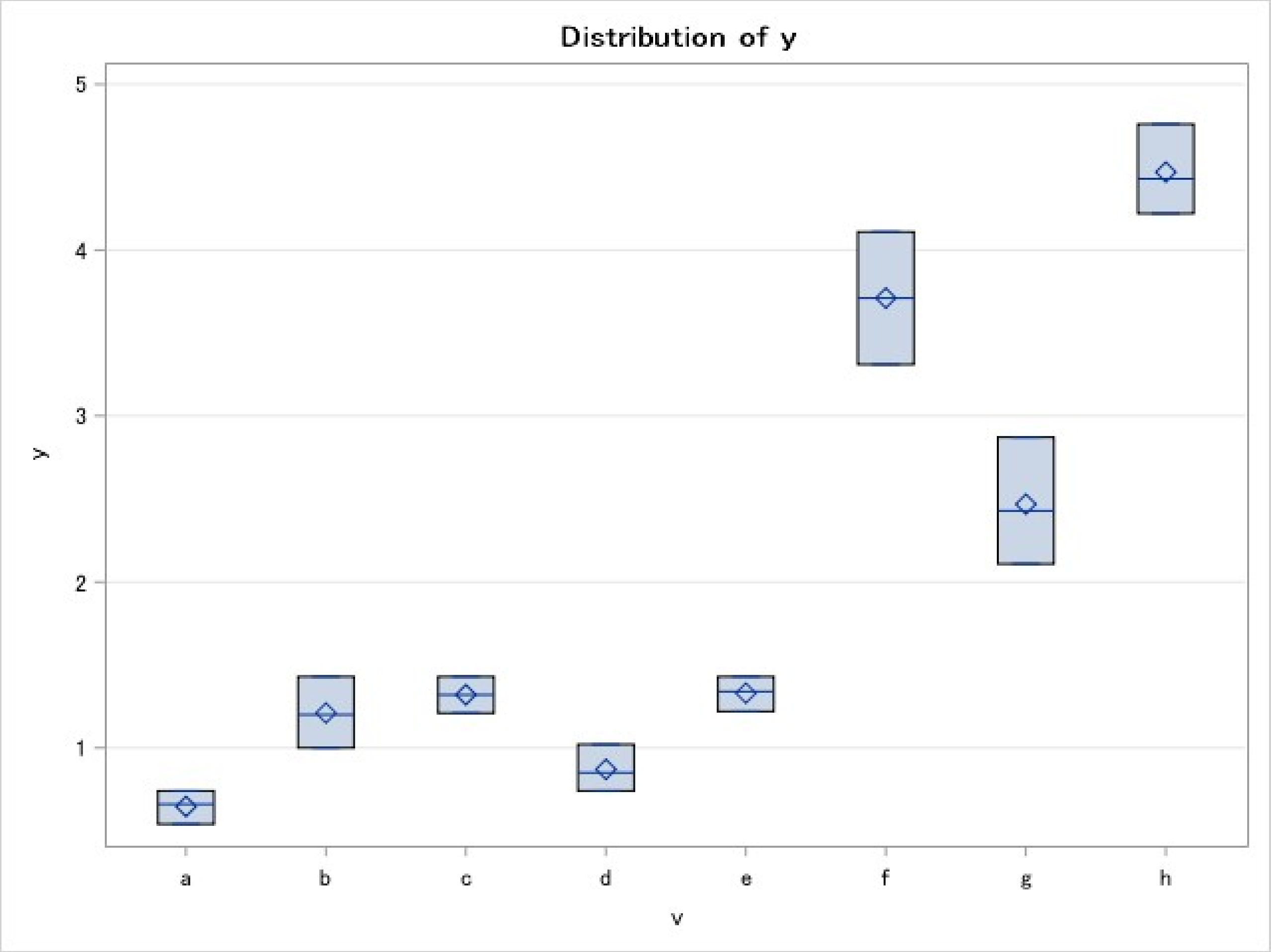

onion day

The ANOVA Procedure

Duncan's Multiple Range Test for y

Note: This test controls the Type I comparisonwise error rate, not the experimentwise error rate.

Alpha	0.05
Error Degrees of Freedom	16
Error Mean Square	0.059904

Number of Means	2	3	4	5	6	7	8
Critical Range	.4236	.4442	.4571	.4660	.4723	.4771	.4807

Means with the same letter are not significantly different.				
Duncan Grouping		Mean	N	v
	A	4.4700	4	h
	B	3.7100	4	f
	C	2.4700	4	g
	D	1.3300	4	e
	D			
	D	1.3200	4	c
	D			
E	D	1.2100	4	b
E				
E	F	0.8700	4	d
	F			
	F	0.6467	4	a

V describes the treatments.

a; Control b; Si c; GA3 d; Si+GA3 e; Heat f; Si+Heat g; GA3+Heat h; Si+GA3+Heat
